# A resonance-assisted intra­molecular hydrogen bond in compounds containing 2-hy­droxy-3,5-di­nitro­benzoic acid and its various deprotonated forms: redetermination of several related structures

**DOI:** 10.1107/S2056989018011544

**Published:** 2018-08-24

**Authors:** Jan Fábry

**Affiliations:** a Institute of Physics, Czech Academy of Sciences, Na Slovance 2, 182 21 Praha 8, Czech Republic

**Keywords:** crystal structure, resonance-assisted hydrogen bonds, refinement constraints, 2-hy­droxy-3,5-di­nitro­benzoic acid, 2-hy­droxy-3,5-di­nitro­benzoate, 2-carb­oxy-4,6-di­nitro­phenolate, 3,5-di­nitro-2-oxidobenzoate

## Abstract

A large number of structural determinations of compounds containing 2-hy­droxy-3,5-di­nitro­benzoic acid (**I**) and its various deprotonated forms, 2-hy­droxy-3,5-di­nitro­benzoate (**II**) or 2-carb­oxy-4,6-di­nitro­phenolate (**III**), are biased. The reason for the bias follows from incorrectly applied constraints or restraints on the *bridging hydrogen*, which is involved in the intra­molecular hydrogen bond between the neighbouring carb­oxy­lic/carboxyl­ate and oxo/hy­droxy groups. This hydrogen bond belongs to the category of resonance-assisted hydrogen bonds. The present article suggests corrections for the following structure determinations that have been published in *Acta Crystallographica*: DUJZAK, JEVNAA, LUDFUL, NUQVEB, QIQJAD, SAFGUD, SEDKET, TIYZIM, TUJPEV, VABZIJ, WADXOR, YAXPOE [refcodes are taken from the Cambridge Structural Database [CSD; Groom *et al.* (2016 ▸). *Acta Cryst*. B**72**, 171–179]. The structural features of the title mol­ecules in all the retrieved structures, together with structures that contain 3,5-di­nitro-2-oxidobenzoate (**IV**), are discussed. Attention is paid to the localization of the above-mentioned *bridging hydrogen*, which can be situated closer to the O atom of the carboxyl­ate/carb­oxy­lic group or that of the hy­droxy/oxo group. In some cases, it is disordered between the two O atoms. The position of the *bridging hydrogen* seems to be dependent on the p*K*_*a*_(base) although with exceptions. A stronger basicity enhances the probability of the presence of a phenolate (**III**). The present article examines the problem of the refinement of such a *bridging hydrogen* as well as that of the hydrogen atoms involved in the hy­droxy and primary and secondary amine groups. It appears that the best model, in many cases, is obtained by fixing the hydrogen-atom position found in the difference electron-density map while refining its isotropic displacement parameter.

## Abstract 2

A large number of structural determinations of compounds containing 2-hy­droxy-3,5-di­nitro­benzoic acid and its various deprotonated forms, 2-hy­droxy-3,5-di­nitro­benzoate or 2-carb­oxy-4,6-di­nitro­phenolate, are biased. The reason for the bias follows from incorrectly applied constraints or restraints on the *bridging hydrogen*, which is involved in the intra­molecular hydrogen bond between the neighbouring carb­oxy­lic/carboxyl­ate and oxo/hy­droxy groups. The present article examines the problem of the location and refinement of such a *bridging hydrogen* in a number of reported compounds. The analysis of the intra­molecular hydrogen bonding is also discussed.

## Chemical context   

2-Hy­droxy-3,5-di­nitro­benzoic acid (**I**; alternatively 3,5-di­nitro­salicylic acid, DNSA), 2-hy­droxy-3,5-di­nitro­benzoate (**II**; alternatively 3,5-di­nitro­salicylate), 2-carb­oxy-4,6-di­nitro­phenolate (**III**) and 3,5-di­nitro-2-oxidobenzoate (**IV**), are mol­ecules that have inter­esting structural and chemical features. Such mol­ecules have been studied because of the proton transfer from the carb­oxy­lic group, which is dependent on its environment (*e.g.* Smith *et al.*, 2007 ▸). Thus, three deprotonated forms of mol­ecule **I** have been observed. The last one, **IV**, is deprived of all of the hydrogen atoms while the others differ in the localization of the hydrogen atom involved in the intra­molecular hydrogen bond between the O atoms of the carboxyl­ate/carb­oxy­lic and the hy­droxy/oxo groups. In the different structures, this hydrogen atom may be closer to either oxygen atom, depending on the properties of each particular structure. In some cases, this hydrogen atom may even be disordered. In the following, it will be referred to as a *bridging hydrogen*.
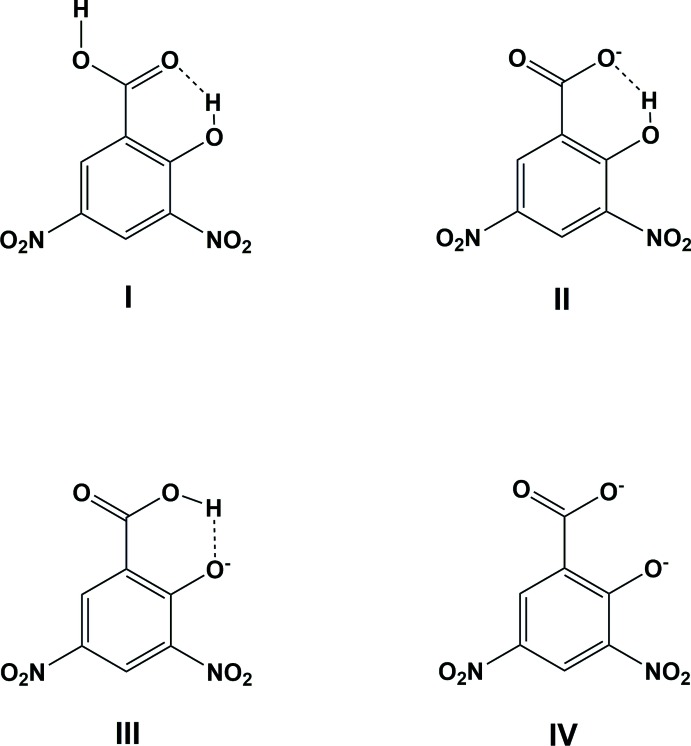


**Figure 1:**
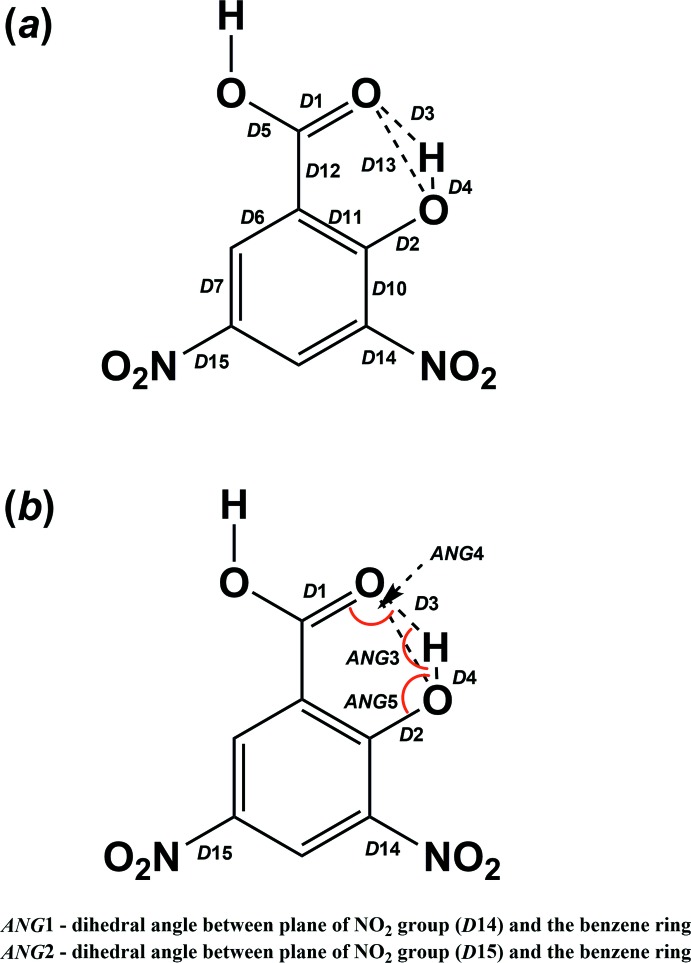
Definition of bonds and various angles in **I**–**IV**.

Such a *bridging hydrogen* is a part of a resonance-assisted moiety (Gilli & Gilli, 2009 ▸) composed of six atoms with the pertinent bonds being *D*1, *D*2, *D*3, *D*4, *D*11 and *D*12, as shown in Fig. 1 ▸ *a*. However, the delocalized bonds can be further extended within the mol­ecule, especially to the C=O/C—OH bond (*D*1/*D*5 in Fig. 1 ▸ *a*). Resonance-assisted hydrogen bonds tend to be stronger and therefore the *bridging hydrogen* should be displaced towards the hydrogen-bond centre. On the other hand, O⋯H⋯O hydrogen bonds with a *bridging hydrogen* that is situated about its centre are usually observed for strong intra­molecular hydrogen bonds with the O⋯O distances being shorter than 2.5 Å (Gilli & Gilli, 2009 ▸), while the O⋯H⋯O angles tend to be close to 180° (Jeffrey, 1995 ▸). The O_carboxyl­ate/carboxyl­icgroup_⋯O_hy­droxy/oxo group_ distance can be as short as 2.41 Å in some 2-hy­droxy-3,5-di­nitro­benzoates (**II**) or 2-carb­oxy-4,6-di­nitro­phenolates (**III**); however, the O⋯H⋯O angle, which is *ca* 160°, situates it in a category of its own.

The above-mentioned features of the intra­molecular O⋯H⋯O hydrogen bond in the mol­ecules considered herein have been ignored on many occasions by incorrectly applied constraints or severe restraints on the O—H distances, 0.82 or 0.84 Å, together with angle constraints/restraints equal to 109° as proposed by *SHELXL* (Sheldrick, 2008 ▸, 2015 ▸).

A robust indication whether the *bridging hydrogen* has been positioned correctly follows from the bond distances C=O/C—O of the involved carboxyl­ate/carb­oxy­lic and hydrox­yl/oxo groups, although there are a few exceptions in which the *bridging hydrogen* is attached to the oxygen forming a slightly shorter C—O distance. These exceptions will be mentioned briefly below. Thus, it seems that a considerable number of the structures containing the mol­ecules **I**–**IV** could have been determined more correctly with a more realistic description of the pertinent hydrogen bond in these mol­ecular fragments.

**Figure 2:**
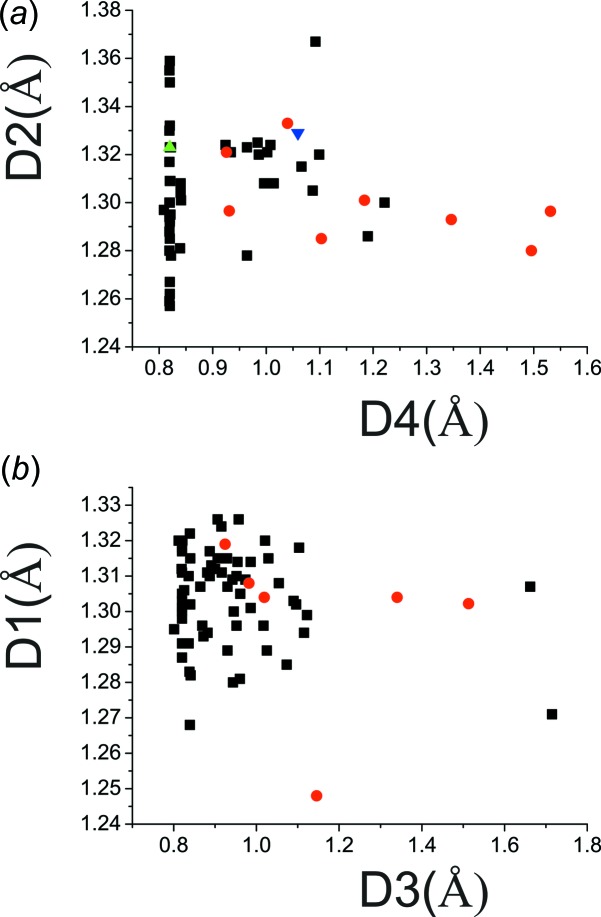
The dependence of bond distances: (*a*) *D*2 on *D*4 for structures that were originally determined as 2-hy­droxy-3,5-di­nitro­benzoate (**II**), or as containing 2-hy­droxy-3,5-di­nitro­benzoic acid (**I**); (*b*) *D*1 on *D*3 for the structures that were determined as 2-carb­oxy-4,6-di­nitro­phenolate (**III**). Colour code for symbols: black squares are the data retrieved from the CSD; red circles are the corrected title structures; green and blue triangles are the original and the corrected structure of LUDFUL, which contains a mol­ecule of 2-hy­droxy-3,5-di­nitro­benzoic acid (**I**).

A search of the Cambridge Structural Database (CSD, Version 3.58, last update May 2017; Groom *et al.*, 2016 ▸) indicated that 27 structures out of 53 reported as 2-hy­droxy-3,5-di­nitro­benzoates (**II**) seem to be suspect; 21 structures out of 70 reported as 2-carb­oxy-4,6-di­nitro­phenolates (**III**) seem to be suspect, and nine structures out of 15 that contain a mol­ecule of 2-hy­droxy-3,5-di­nitro­benzoic acid (**I**) also appear to be suspect. Figs. 2 ▸ *a* and 2 ▸ *b* illustrate this situation for 2-hy­droxy-3,5-di­nitro­benzoates (**II**) and 2-carb­oxy-4,6-di­nitro­phenolates (**III**), respectively.

It is plausible to expect that the environment affects the position of the *bridging hydrogen*. Therefore, it can be assumed that the proton transfer stemming from the carboxyl group will affect its position.

The data for the suspect structures published in *Acta Crystallographica* were retrieved from the journal’s web page and recalculated. Tables 1 ▸ and 2 ▸ contain an overview of those structures, which were successfully redetermined. In the following, these structures are referred to by their CSD refcodes; for the pertinent chemical names, see Table 2 ▸.

Notably, JEVNAA turns out not to be a substituted benzoate but a phenolate. NUQVEB though reported as a substituted benzoate turns out to be present in a disordered benzoate and a phenolate form. QIQJAD though reported as a disordered benzoate and a phenolate turns out to be a substituted benzoate. SAFGUD was reported as a substituted benzoate but turns out to be a phenolate. WADXOR was reported as a substituted benzoate that is disordered over two positions but it turns out to be present both in a dominant benzoate as well as in a minor phenolate form. Finally, SEDKET was originally determined as a substituted phenolate but it turns out to be a benzoate.

Some of the retrieved structures were difficult or impossible to recalculate with sufficient accuracy: HILPOI (trimetho­prim­ium 3,5-di­nitro­salicylate; Subashini *et al.*, 2007 ▸) because of an abnormally low proportion of observed reflections (moreover the *bridging hydrogen* H6*a* is situated out of the plane between the carboxyl­ate and hy­droxy oxygen atoms, which seems to indicate an error) and VUZNEK (3,4-di­amino­pyridinium 2-carb­oxy-4,6-di­nitro­phenolate; Hemamalini & Fun, 2010*b* ▸) because of the disorder present in the structure.

## Refinement of the title structures   

For each structure, two methods have been applied for the refinement of the hydrogen atoms involved in hydrogen bonding. In *Method 1*, the positions of the *bridging hydrogens* as well as those of the hy­droxy, primary and secondary amine and ammonium hydrogen atoms were fixed after their local­ization in the difference electron-density maps while their displacement parameters were refined. In *Method 2*, the positional parameters of the latter hydrogen atoms were refined while their displacement parameters were constrained in the usual manner: *U*_iso_(H) = 1.2*U*_eq_(N_amine_) or *U*_iso_(H) = 1.5*U*_eq_(O_hy­droxy_) or *U*_iso_(H) = 1.5*U*_eq_(N_ammonium_).

The appropriate sections of the difference electron-density maps of the title structures (see supplementary Fig. S1) show regions with the hy­droxy, amine and ammonium hydrogen atoms. These sections comprise the maps that were obtained after the refinement of the models without the pertinent hydrogen atoms as well as the maps that were calculated by either refinement method. It can be seen from the supplementary Fig. S1 that one of the reasons that hinders the correct localization of the hydrogen atoms involved in the hydrogen bonds is an apparent non-spherical electron density of the donor and acceptor atoms. Thus, hydrogen-atom local­ization by X-ray diffraction is hindered not only by its weak scattering power, but also by the polarization of its electron density resulting from the proximity of the acceptor and by the asphericity of the electron density of the donor and acceptor atoms. Therefore, refinement *Method 1* was given preference. The hydrogen bonds in the title structures are listed in Table 3 ▸, which shows that there might be quite a large difference between the results with the fixed and the refined positional parameters of such hydrogen atoms. In the following, a detailed description of the refinement of the recalculated structures is given:

**DUJZAK** (Zhang & Jian, 2009 ▸): C—H_ar­yl_ were constrained to be equal to 0.93 Å while *U*_iso_(H_ar­yl_) = 1.2*U*_eq_(C_ar­yl_). The position of the *bridging hydrogen* H3*b* as well as those of the hy­droxy hydrogen atoms H1*aa* and H2*aa* were located in a difference electron-density map. Their positional parameters were fixed during the refinement while their isotropic displace­ment parameters were refined.

**JEVNAA** (Huang *et al.*, 2007 ▸): C—H_ar­yl_ were constrained to be equal to 0.93 Å while *U*_iso_(H_ar­yl_) = 1.2*U*_eq_(C_ar­yl_). The position of the *bridging hydrogen* H1*a* as well as those of the secondary amine hydrogen atoms H2*a* and H4*a* were located in the difference electron-density map. Their positional parameters were fixed during the refinement while their isotropic displacement parameters were refined.

**LUDFUL** (Senthil Kumar *et al.*, 2002 ▸): C—H_ar­yl_ were constrained to be equal to 0.93 Å while *U*_iso_(H_ar­yl_) = 1.2*U*_eq_(C_ar­yl_). The position of the *bridging hydrogen* H3*a* as well as that of the hydroxy hydrogen atom H1*a* were located in a difference electron-density map. Their positional parameters were fixed during the refinement while their isotropic displacement parameters were refined.

**NUQVEB** (Hemamalini & Fun, 2010*a* ▸): The subroutine *TwinRotMax* of *PLATON* (Spek, 2009 ▸) indicated non-merohedral twinning: *h*2 = −*h*1; *k*2 = −*k*1; *l*2 = −0.488 *h*1 − 0.153*k*1 + *l*1. The refinement was carried out on the non-overlapped reflections only. The refined value of the second domain fraction converged to the value −0.0006 (4). Therefore the value of the second domain fraction was set to 0 and was not refined further. C—H_ar­yl_ and C—H_meth­yl_ were constrained to be equal to 0.95 and 0.98 Å, respectively. *U*_iso_(H_ar­yl_) = 1.2*U*_eq_(C_ar­yl_) and *U*_iso_(H_meth­yl_) = 1.5*U*_eq_(C_meth­yl_). The positions of the disordered *bridging hydrogens* H1*o*1 and H1*o*7 as well as those of the primary (H2*a*, H2*b*) and the secondary amine hydrogen atoms (H1*a*) were located in a difference electron-density map. Their positional parameters were fixed during the refinement while their isotropic displacement parameters were refined; in the case of the *bridging hydrogens* H1*o*1 and H1*o*7, their isotropic displacement parameters were refined to be equal while their occupational parameters were refined under the condition that their sum was equal to 1.

**QIQJAD** (Sridhar *et al.*, 2013 ▸): The subroutine *TwinRotMax* of *PLATON* (Spek, 2009 ▸) indicated non-merohedral twinning: *h*2 = −1.018*h*1 + 0.054*k*1; *k*2 = −0.673*h*1 + 1.018*k*1; *l*2 = −0.039*h*1 + 0.116*k*1 − *l*1. The refined value of the second domain fraction converged to the value 0.028 (13). Therefore the value of the second domain fraction was set to 0 and was not refined further. C—H*sp* ^2^ and C—H_meth­yl_ were constrained to equal to 0.93 and 0.96 Å, respectively. *U*_iso_(H*sp* ^2^) = 1.2*U*_eq_(C*sp* ^2^) and *U*_iso_(H_meth­yl_) = 1.5*U*_eq_(C_meth­yl_). The positions of the *bridging hydrogen* H3*o* and those of the primary (H3*n*, H4*n*, H5*n*, H6*n*) as well as of the secondary (H2*n*) amine hydrogen atoms were located in a difference electron-density map. Their positional parameters were fixed during the refinement while their isotropic displacement parameters were refined.

**SAFGUD** (Wang *et al.*, 2012 ▸)**:** C—H_ar­yl_ were constrained to be equal to 0.93 Å while *U*_iso_(H_ar­yl_) = 1.2*U*_eq_(C_ar­yl_). The *bridging hydrogen* H7 was located in a difference electron-density map and its position was fixed while its isotropic displacement parameter *U*_iso_(H7) was refined.

**SEDKET (**Wei *et al.*, 2012 ▸): The non-centrosymmetric structure is composed of the light atoms only (the heaviest atom is O) and the data collection was carried out with Mo *K*α radiation. The article by Wei *et al.* (2012 ▸) does not indicate whether the Friedel pairs were merged and nor does it contain the value of the Flack parameter. The Flack parameter was set to 0.5 without being refined in the present model. C—H_ar­yl_ and C—H_meth­yl_ were constrained to be equal to 0.93 and 0.96 Å, respectively. *U*_iso_(H_ar­yl_) = 1.2*U*_eq_(C_ar­yl_) and *U*_iso_(H_meth­yl_) = 1.5*U*_eq_(C_meth­yl_). The position of the *bridging hydrogen* H2*a* as well as those of the secondary amine hydrogen atoms H1 and H2 were located in a difference electron-density map. Their positional parameters were fixed during the refinement while their isotropic displacement parameters were refined.

**TIYZIM** (Yamuna *et al.* (2014 ▸): C—H_ar­yl_ and C—H_methyl­ene_ were constrained to be equal to 0.95 and 0.99 Å, respectively. *U*_iso_(H_ar­yl_) = 1.2*U*_eq_(C_ar­yl_) and *U*_iso_(H_methyl­ene_) = 1.5*U*_eq_(C_methyl­ene_). The position of the *bridging hydrogen* H2*b* as well as those of the ammonium hydrogen atoms (H3*aa*, H3*ab*, H3*ac*) were found in a difference electron-density map. Their positional parameters were fixed during the refinement while their isotropic displacement parameters were refined; in the case of the ammonium hydrogen atoms (H3*ab*, H3*ac*,), their displacement parameters were constrained to be equal to that of H3*aa*.

**TUJPEV** (Malathy *et al.*, 2015 ▸): C—H_ar­yl_ were constrained to be equal to 0.93 Å while *U*_iso_(H_ar­yl_) = 1.2*U*_eq_(C_ar­yl_). C—H_meth­yl_ were constrained to be equal to 0.96 Å while *U*_iso_(H_meth­yl_) = 1.5*U*_eq_(C_meth­yl_). The position of the *bridging hydrogen* H6*a* as well as those of the secondary amine group H2*a* and of the ammonium hydrogen atoms H1*a*, H1*b* and H1*c* were found in a difference-electron map. Their positional parameters were fixed during the refinement while their isotropic displacement parameters were refined; in the case of the ammonium hydrogen atoms (H1*b*, H1*c*), their displacement parameters were constrained to be equal to that of H1*a*.

**VABZIJ** (Hemamalini & Fun, 2010*c* ▸): C—H_ar­yl_, C—H_meth­yl_, C—H_methine_ were constrained to be equal to 0.93, 0.96 and 0.98 Å, respectively. *U*_iso_(H_ar­yl_) = 1.2*U*_eq_(C_ar­yl_), *U*_iso_(H_methine_) = 1.2*U*_eq_(C_methine_), *U*_iso_(H_meth­yl_) = 1.5*U*_eq_(C_meth­yl_). The position of the *bridging hydrogen* H7 as well as those of the secondary amine hydrogen atom H1*n*4 and of the water hydrogen atoms H1*w*1 and H1*w*2 were located in a difference electron-density map. Their positional parameters were fixed during the refinement while their displacement parameters were refined.

**WADXOR** (Smith & Lynch, 2016 ▸): The non-centrosymmetric structure is composed of light atoms only (the heaviest atoms present in the structure are oxygens) and the data collection was carried out with Mo *K*α radiation. The original article reported the refined Flack parameter to be equal to −0.1 (13); however, the refinement using *JANA*2006 (Petříček *et al.*, 2014 ▸) did not converge and therefore the Flack parameter was set to 0.5 without being refined. C—H_ar­yl_ and C—H_methyl­ene_ were constrained to be equal to 0.95 and 0.99 Å, respectively, except for the distances between the methyl­ene atom C11 and the attached hydrogen atoms H12*a* and H13*a*, which were restrained to 0.99 (1) Å (Müller, 2009 ▸). [The reason for the different treatment of the latter methyl­ene group was its vicinity to the disordered methyl­ene groups centered on C10 and C12*a*.] *U*_iso_(H_ar­yl_) = 1.2*U*_eq_(C_ar­yl_) and *U*_iso_(H_methyl­ene_) = 1.2*U*_eq_(C_methyl­ene_). There were two types of occupational disorder present in the structure. The first one was related to the fragments with the methyl­ene carbon atoms C9*a*, C10*a* and the attached respective pairs of hydrogen atoms H91*a*, H92*a* and H10*a*, H11*a*, as well as to C13*a* and C12*a* with the attached respective pairs of hydrogen atoms H16*a*, H17*a* and H14*a*, H15*a*. The occupation parameter of C13 was refined while those of the related atoms were either set equal to that of C13 (*i.e.* C12*a* and attached hydrogen atoms) or its complement to 1 (C9*a* and C10*a* and attached hydrogen atoms). The displacement parameters of the disordered pairs of atoms C9*a* and C13*a* as well as C10*a* and C12*a* were set to be equal, *i.e.* that of C13*a* equalled that of C9*a* while that of C10*a* equalled that of C12*a*. The second type of occupational disorder referred to the fragments C2*b*—H61*b*, C2*b*–O2*b*—H2*b* and C6*b*—H6*b*, C6*b*—O21*b*—H21*b*. This means that the occupation parameters of H61*b*, H21*b* were set equal to the refined occupational parameter of O21*b* while being complements to 1 for H6*b*, O2*b*, H2*b*. The positions of the *bridging hydrogens* H2*b* and H21*b* as well as that of the primary amine hydrogen atom H8*a* were located in a difference electron-density map. Their positional parameters were fixed during the refinement while their isotropic displace­ment parameters were refined; in the case of *bridging hydrogens* H2*b* and H21*b*, their isotropic displacement parameters were constrained to be equal.

**YAXPOE** (Dayananda *et al.*, 2012 ▸): C—H_ar­yl_ and C—H_methyl­ene_ were constrained to equal to 0.95 and 0.99 Å, respectively. *U*_iso_(H_ar­yl_) = 1.2*U*_eq_(C_ar­yl_) and *U*_iso_(H_methyl­ene_) = 1.5*U*_eq_(C_methyl­ene_). The *bridging hydrogen* H7 was located in a difference electron-density map. Its positional parameters were fixed while *U*_iso_(H7*a*) was refined. A high instability factor Δ in the weighting scheme (0.0064) was applied in order to avoid a large number of reflections with (*I*_obs_ − *I*_calc_)/σ(*w*) > 10 where σ(*w*) = [σ^2^(*I*) + Δ*I* ^2^]^−1/2^. [This condition generates A alerts for Δ = 0.0004, which has been used in other refinements of the title structure, when running *checkCIF* (Spek, 2009 ▸).] The residual electron-density map contains peaks which are difficult to inter­pret (see supplementary Fig. S1).

## Discussion of the inter­dependence of bond lengths and angles   

**Figure 3:**
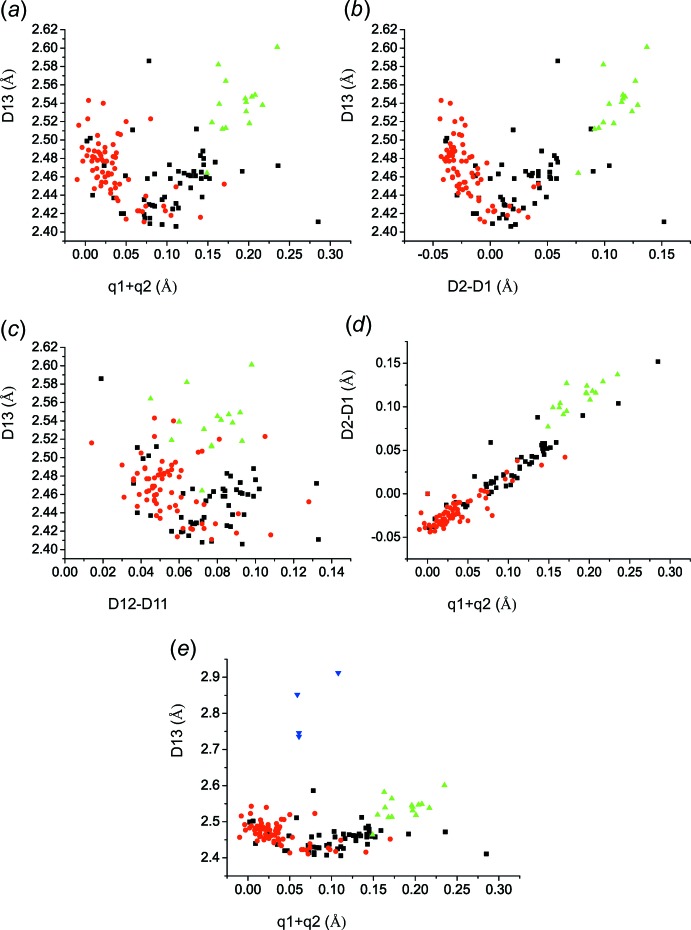
The dependence of distances: (*a*) *D*13 on (*q*1 + *q*2); (*b*) *D*13 on *D*2 − *D*1; (*c*) *D*13 on *D*12—*D*11; (*d*) *D*2 − *D*1 on (*q*1 + *q*2); (*e*) *D*13 on (*q*1 + *q*2), also for the structures with 3,5-di­nitro-2-oxidobenzoate (**IV**), which are shown as blue triangles. Colour code for symbols: green triangles refer to the structures with 2-hy­droxy-3,5-di­nitro­benzoic acid (**I**), black squares are the structures with 2-hy­droxy-3,5-di­nitro­benzoate (**II**), and red circles are the structures with 2-carb­oxy-4,6-di­nitro­phenolates (**III**).

For this discussion, the definition of the various bonds and angles in the moieties of **I**–**IV** (shown in the scheme), are illustrated in Figs. 1 ▸ *a* and 1*b*, respectively. As already pointed out, the dependence *D*2 on *D*4 and *D*1 on *D*3 (Fig. 2 ▸) has shown that a large number of structures are biased by incorrectly applied constraints or restraints on the *bridging hydrogen*. However, a dubious or incorrect localization of the *bridging hydrogen* or the acid hydrogen is believed to affect the positions of the non-hydrogen atoms only minutely, and therefore even the biased structures can be considered further. The parameters *q*1 = *D*2 − *D*1 and *q*2 = *D*12 − *D*11 express the electron delocalization within the fragment *D*1–*D1*2–*D*11–*D*2. The introduction of the parameters *q*1 and *q*2 follows an analogous discussion of resonance-assisted hydrogen bonds in the enol forms of β-diketone fragments (Gilli *et al.*, 1989 ▸, 2009 ▸). Fig. 3 ▸ *a* shows that the distance where the structures with 2-carb­oxy-4,6-di­nitro­phenolates (**III**; red circles) transform into 2-hy­droxy-3,5-di­nitro­benzoates (**II**; black squares) corresponds to the shortest distance *D*13_min_ ≃ 2.41 Å, which in turn corresponds to (*q*1 + *q*2) ≃ 0.08 Å. This implies that this is the region where the *bridging hydrogen* has the greatest tendency to be situated about the centre of the O⋯O intra­molecular hydrogen bond or disordered about it. A very similar dependence is shown in Fig. 3 ▸ *b*, where only distances *D*1 and *D3* are compared. The observed dependence means that the elongation of one C—O bond takes place mostly at the cost of the shortening of the neighbouring C=O bond; in other words, the distance between these two O atoms, D13 ≃ [(*D*13_min_)^2^ + (*D*2 − *D*1)^2^]^1/2^ (Fig. 1) ▸. Table 4 ▸ lists the structures in which the title mol­ecules are present in different forms. In the recalculated structure of SEDKET (Table 2 ▸) and *e.g.* the reported structures of KEZJIJ (Song *et al.*, 2007 ▸) and KEZJIJ01 (Smith *et al.*, 2007 ▸) that refer to the structure determination of 2-(pyridin-2-yl)pyridinium 2-carb­oxy-4,6-di­nitro­phenolate, the *bridging hydrogen* is attached to the O atom having the shorter C—O bond distance.

Fig. 3 ▸ *a* and 3*b* also show that the *bridging hydrogen* cannot be situated near the centre of the intra­molecular O⋯O hydrogen bond in structures with 2-hy­droxy-3,5-di­nitro­benzoic acid (**I**). Fig. 3 ▸ *c* shows a similar dependence of *D*13 on (*D*12 − *D*11). It can be seen that the adjacent C—C conjugated bonds are less, but still sensitive to the bonding of the hy­droxy hydrogen atom to one of the neighbouring C—O groups. These properties indicate that the O⋯H⋯O hydrogen bonding with the pertinent O⋯O distance *D*13 belongs to the category of resonance-assisted hydrogen bonds (Gilli *et al.*, 1989 ▸, 2009 ▸; Sobczyk *et al.*, 2005 ▸).

Fig. 3 ▸ *d* compares both dependences shown in Figs. 3 ▸ *a* and 3*b*. It can be seen that the dependence of (*D*2 − *D*1) on (*q*1 + *q*2) is fairly linear. The dependence seems to show the narrowest spread for the 2-hy­droxy-3,5-di­nitro­benzoates (**II**), which are represented by the black squares. Importantly, the line for each class of mol­ecules inter­cepts the *D*2 − *D*1 axis at different values. The structures that contain 2-hy­droxy-3,5-di­nitro­benzoic acid (**I**) mol­ecules (green triangles) are clearly separated from the rest of the structures although they show a similar trend. Figs. 3 ▸ *a*–3*d* also show outliers that do not fit the overall trends and which are most probably the structures determined as 2-hy­droxy-3,5-di­nitro­benzoates (**II**) instead of 2-carb­oxy-4,6-di­nitro­phenolates (**III**) and *vice versa*. Fig. 3 ▸ *e* shows the same as Fig. 3 ▸ *a* except for the addition of a few known structures that contain a 3,5-di­nitro-2-oxidobenzoate (**IV**), which are indicated by blue triangles. Their positions can be explained by the fact that the carboxyl­ate groups are substanti­ally inclined to the benzene ring in such compounds, which causes elongation of the distance between the carboxyl­ate and oxo group, and these mol­ecules will not be considered further.

**Figure 4:**
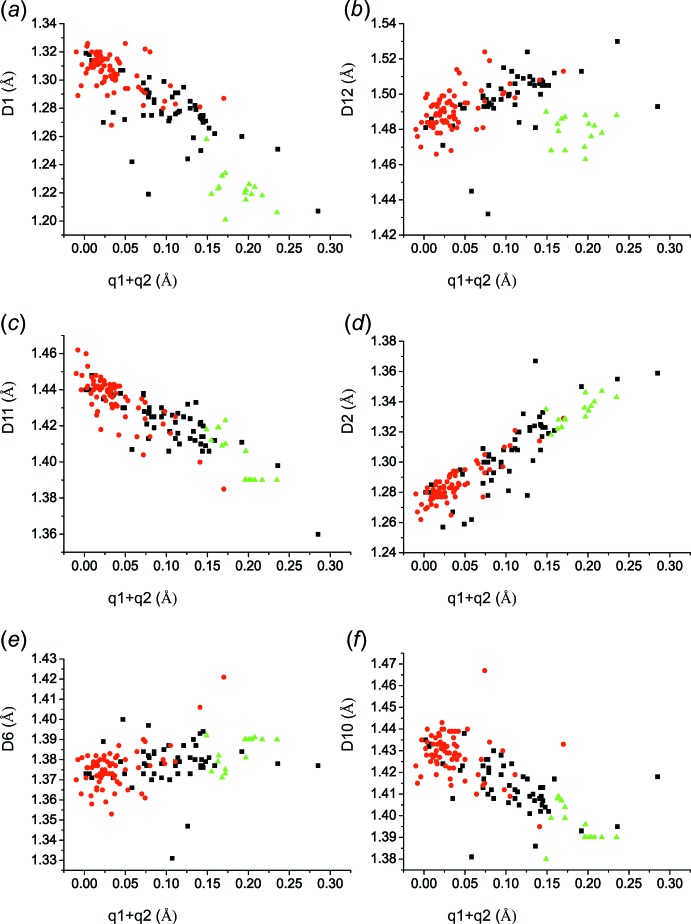
The dependence of bond distances: (*a*) *D*1 on (*q*1 + *q*2); (*b*) *D*12 on (*q*1 + *q*2); (*c*) *D*11 on (*q*1 + *q*2); (*d*) *D*2 on (*q*1 + *q*2); (*e*) *D*6 on (*q*1 + *q*2); (*f*) *D*10 on (*q*1 + *q*2). The colour code for the symbols is the same as in Fig. 3 ▸.

The alternation of the inclinations (Fig. 4 ▸ *a*–4*d*) of the dependences of *D*1, *D*12, *D*11, and *D*2 on (*q*1 + *q*2) are in agreement with the delocalization of the electron density in these bonds. The 2-hy­droxy-3,5-di­nitro­benzoic acid (**I**) mol­ecules (green triangles) and the 2-hy­droxy-3,5-di­nitro­benzoates (**II;** black squares) are situated apart from the 2-carb­oxy-4,6-di­nitro­phenolates (**III;** red circles) in the given figures. The fact that *D*1 tends to be shortest in 2-hy­droxy-3,5-di­nitro­benzoic acid (**I**) mol­ecules (Fig. 4 ▸ *a*) can be explained by the elongation of bond *D*5 in the latter mol­ecules because of the attachment of the hydrogen atom and the concomitant shortening of *D*1. The bond lengths *D*1 (Fig. 4 ▸ *a*) are equal to 1.28–1.30 Å at (*q*1 + *q*2) ≃ 0.08 where the highest probability for the occurrence of a symmetric intra­molecular O⋯H⋯O hydrogen bond takes place. The corresponding values of *D*12, *D*11, *D*2, *D*6 and *D*10 are 1.49 Å (Fig. 4 ▸ *b*), 1.43 Å (Fig. 4 ▸ *c*), 1.30 Å (Fig. 4 ▸ *d*), 1.37–1.39 Å (Fig. 4 ▸ *e*) and 1.41–1.43 Å (Fig. 4 ▸ *f*).

**Figure 5:**
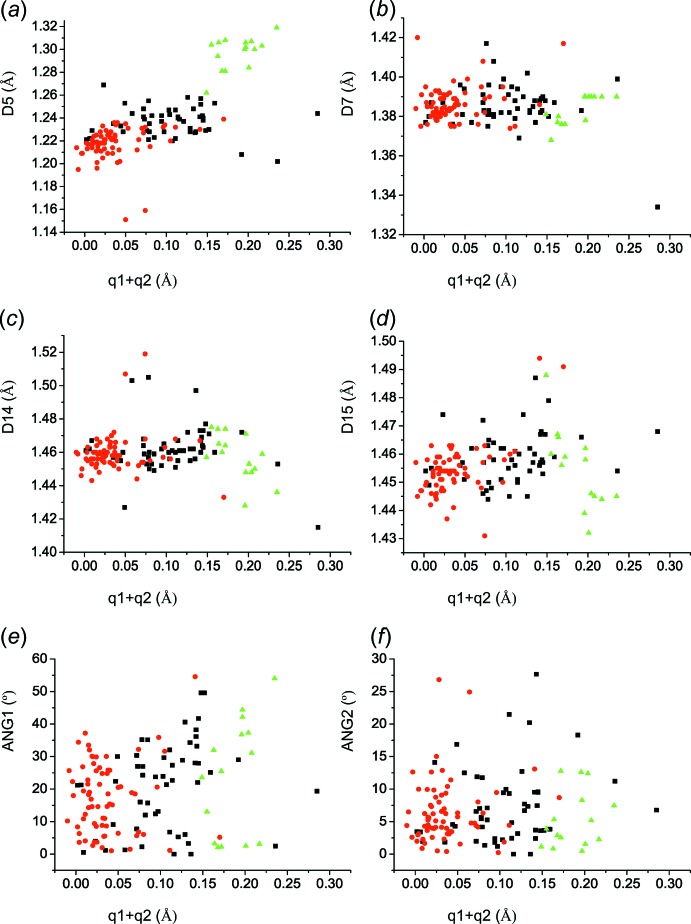
The dependence of bond distances: (*a*) *D*5 on (*q*1 + *q*2); (*b*) *D*7 on (*q*1 + *q*2); (*c*) *D*14 on (*q*1 + *q*2). The dependence of dihedral angles: (*e*) *ANG*1 on (*q*1 + *q*2); (*f*) *ANG*2 on (*q*1 + *q*2). [*ANG*1 and *ANG*2 are the dihedral angles of the nitro groups involving bonds *D*14 and *D*15, respectively, toward the ring plane.] The colour code of the symbols is the same as in Fig. 3 ▸.

Fig. 5 ▸ *a* shows the dependence of *D*5 on (*q*1 + *q*2). Comparing Fig. 5 ▸ *a* to Fig. 4 ▸ *a*, which shows the dependence of *D*1 on (*q*1 + *q*2), an indirect proportionality of both dependences can be observed. The bond length *D*5 is equal to 1.22–1.24 Å for (*q*1 + *q*2) ≃ 0.08 Å. The dependence of *D*5 on (*q*1 + *q*2) (Fig. 5 ▸ *a*) is similar to that of bond *D*12 (Fig. 4 ▸ *b*) in 2-hy­droxy-3,5-di­nitro­benzoates (**II**) and 2-carb­oxy-4,6-di­nitro­phenolates (**III**), but not in mol­ecules of 2-hydroxo-3,5-di­nitro­benzoic acid (**I**). It is inter­esting that 2-hy­droxy-3,5-di­nitro­benzoic acid (**I**) mol­ecules are in line with other forms of the title mol­ecules for the dependences in Fig. 5 ▸ *c* and Fig. 4 ▸ *d*. Bond *D*7 is rather distant from the carb­oxy­lic group (Fig. 5 ▸ *b*) and the delocalization within the pyridine ring is no longer clear. The same holds for bonds *D*14 and *D*15 (Figs. 5 ▸ *c* and 5*d*). Figs. 5 ▸ *e* and 5*f* show the inclinations, *ANG*1 and *ANG*2, of the nitro groups involving bonds *D*14 and *D*15, respectively, toward the ring plane.

**Figure 6:**
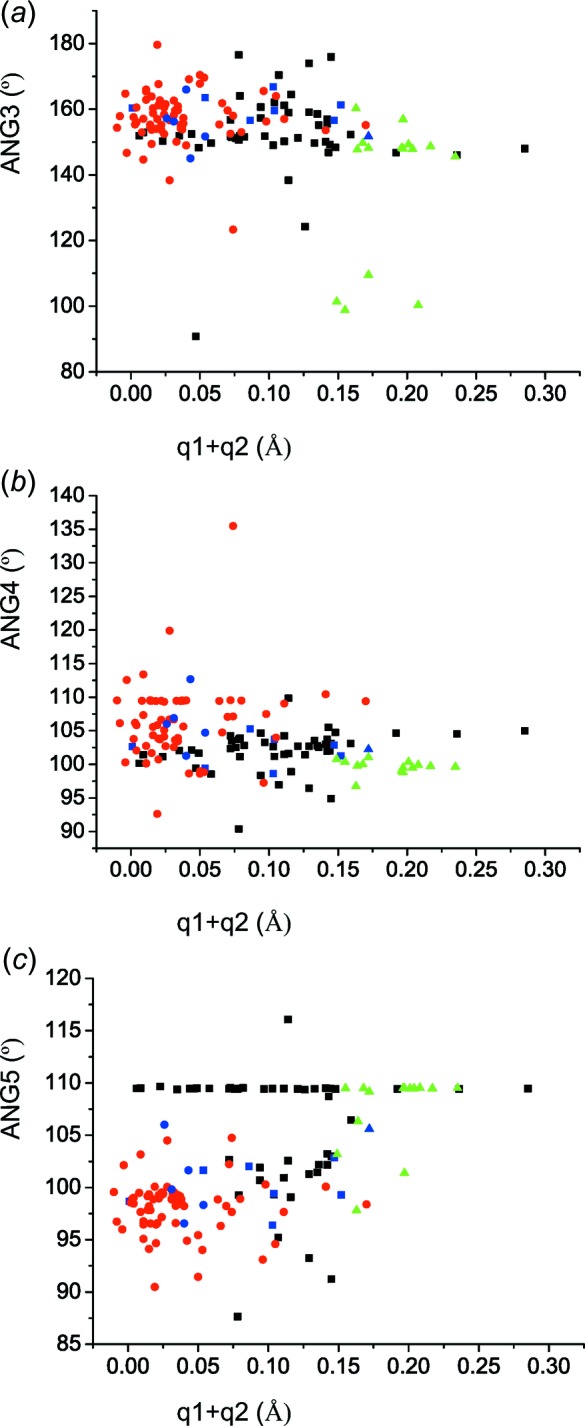
(*a*) Dependence of the O⋯H⋯O angle *ANG*3 on (*q*1 + *q*2); (*b*) dependence of *ANG*4 on (*q*1 + *q*2); (*c*) dependence of *ANG*5 on (*q*1 + *q*2). Colour code for symbols: green triangles refer to the structures with 2-hy­droxy-3,5-di­nitro­benzoic acid (**I**), black squares are the structures with 2-hy­droxy-3,5-di­nitro­benzoate (**II**), and red circles are the structures with 2-carb­oxy-4,6-di­nitro­phenolate (**III**); blue triangles, squares and circles are the recalculated structures with 2-hy­droxy-3,5-di­nitro­benzoic acid (**I**), 2-hy­droxy-3,5-di­nitro­benzoate (**II**) and 2-carb­oxy-4,6-di­nitro­phenolate (**III**), respectively.

Fig. 6 ▸ *a*–6*c* show dependences in which the localization of the *bridging hydrogen* takes place. It seems that the most obtuse angles of O⋯H⋯O (*ANG*3) occur for (*q*1 + *q*2) in the range <0.06–0.10> Å, *i.e.* for the shortest distances of *D*13 (2.41 Å). It is questionable whether the position of a *bridging hydrogen* in the transition zone between 2-hy­droxy-3,5-di­nitro­benzoates (**II**) and 2-carb­oxy-4,6-di­nitro­phenolates (**III**) facilitates its positional disorder, which occurs *e.g.* in NUQVEB, because of the impossibility of angle *ANG*3 approaching 180°. The dependence of the angles *ANG*4 and *ANG*5 (Fig. 1 ▸ *b*) shows once more the effect of incorrectly applied constraints, which are manifested by values close to 109.54° (*cf*. Figs. 2 ▸ *a* and 2*b*).

The previous discussion has shown the correlations of *D*1 and *D5* on (*q*1 + *q*2) (Figs. 4 ▸ *a* and 5*a*, respectively), and the indirect dependence of *D*1 on *D*5. Therefore, the position of the *bridging hydrogen* is expected to be related to the environment of the mol­ecules, *i.e.* to be dependent on Δp*K*_*a*_ = p*K*_*a*_(base) − p*K*_*a*_(acid). The value of Δp*K*_*a*_ is correlated with the occurrence of a structure where the base and the acid components are not ionized, thus forming a co-crystal (Δ < 0), or ionized forming a salt (Δp*K*_*a*_ > 3) (Childs *et al.*, 2007 ▸). It is difficult to predict the form in which the acid and the base are present for 0 < Δp*K*_*a*_ < 3 (Childs *et al.*, 2007 ▸).

In Table 4 ▸, the structures are ordered according to ascending values of the p*K*_*a*_ values of the bases, *i.e.* according to increasing basicity. The corresponding values of Δp*K*_*a*_ are compared with (*q*1 + *q*2) and *D*13. The p*K*_*a*_ of 2-hy­droxy-3,5-di­nitro­benzoic acid (**I**; 3,5-di­nitro­salicylic acid) is reported as 2.18 (Smith & Wermuth, 2014 ▸; Hemamalini & Fun, 2010*a* ▸), although a value of 1.53 has been reported in the literature (https://www.chemicalbook.com/ProductMSDSDetailCB9172047_EN.htm). The weakest bases given at the top of Table 4 ▸ are not able to deprotonate the title mol­ecule, which remains in the form of 2-hy­droxy-3,5-di­nitro­benzoic acid (**I**). On the other hand, the bases with the largest values of p*K*_*a*_ (see the bottom of Table 3 ▸) are able to deprive the title mol­ecule of the hy­droxy and acid hydrogen atoms, so in such cases the resulting mol­ecule would be in the form of 3,5-di­nitro-2-oxidobenzoate (**IV**). The compounds with moderate basicities are able to deprotonate the acid hydrogen atom but not the *bridging hydrogen;* hence, the resulting forms are 2-hy­droxy-3,5-di­nitro­benzoate (**II**) or 2-carb­oxy-4,6-di­nitro­phenolate (**III**). These structures appear in the inter­mediate region of Table 4 ▸. A more radical transfer of the acid hydrogen atom should cause a more significant shortening of bond *D*5, which should be concomitant with the elongation of bond *D*1. Such an elongation of bond *D*1 (*cf.* Fig. 1 ▸ *a*) should support the formation of a 2-carb­oxy-4,6-di­nitro­phenolate (**III**).

## Summary   

(1) The *bridging hydrogen* in the mol­ecules discussed (**I**–**III**) is involved in a resonance-assisted hydrogen bond, which is part of a hexa­gonal 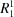
(6) ring. The system of conjugated bonds in the title mol­ecules, however, comprises more atoms than the ring in which the *bridging hydrogen* is involved. In particular, the whole carboxyl­ate/carb­oxy­lic group affects the discussed intra­molecular O⋯H⋯O hydrogen bond.

(2) The transition region between the forms of 2-hy­droxy-3,5-di­nitro­benzoates (**II**) and 2-carb­oxy-4,6-di­nitro­phenolates (**III**) takes place for C—O (*D*1) ≃ 1.28–1.30 Å, C—O (*D*2) ≃ 1.30 Å, O⋯O distance *D*13 ≃ 2.41 Å and (*q*1 + *q*2) ≃ 0.08 Å. Simultaneously, the highest probability for the presence of the *bridging hydrogen* to be in the centre of the hydrogen bond is expected in this transition region. However, the hydrogen atom can also be disordered over two positions as occurs in NUQVEB.

(3) The *bridging hydrogen* in the discussed intra­molecular hydrogen bond can be situated at the centre between both oxygen atoms with approximately equal C—O bond distances. Therefore, the *bridging hydrogen* can not be situated at the centre of the intra­molecular O⋯H⋯O hydrogen bond in compounds containing 2-hy­droxy-3,5-di­nitro­benzoic acid (**I**).

(4) In some rare cases (*e.g.* recalculated SEDKET, KEZJIJ and KEZJIJ01), the *bridging hydrogen* is bonded to the oxygen atom that forms the shorter C—O bond distance (Table 3 ▸). It would be of inter­est to see how the localization of the *bridging hydrogen* develops with changing temperature in such cases.

(5) Table 4 ▸ shows the occurrence of the different forms of the mol­ecules (see scheme) and the dependence on basicity. Alhough it would be expected that the increasing basicity should support the occurrence of 2-carb­oxy-4,6-di­nitro­phenolates (**III**) and, of course, for very strong bases, 3,5-di­nitro-2-oxidobenzoates (**IV)**, there are many exceptions to this rule.

(6) The positioning of the hydrogen atoms can be affected by the asphericity of the electron density of the donor and acceptor atoms.

(7) It is essential to calculate difference electron-density maps in order to locate correctly the *bridging hydrogen* atom, and any other hydrogen atoms involved in hydrogen bonding.

(8) The present overview has shown that the application of constraints and restraints is frequently incorrect.

## Supplementary Material

Crystal structure: contains datablock(s) global, DUJZAK, JEVNAA, NUQVEB, QIQJAD, SEDKET, VABZIJ, WADXOR, YAXPOE, LUDFUL, SAFGUD, TIYZIM, TUJPEV. DOI: 10.1107/S2056989018011544/su5452sup1.cif

Structure factors: contains datablock(s) I Structure factors: contains datablock(s) I. DOI: 10.1107/S2056989018011544/su5452Isup2.hkl

Structure factors: contains datablock(s) II. DOI: 10.1107/S2056989018011544/su5452IIsup3.hkl

Structure factors: contains datablock(s) III. DOI: 10.1107/S2056989018011544/su5452IIIsup4.hkl

Structure factors: contains datablock(s) IV. DOI: 10.1107/S2056989018011544/su5452IVsup5.hkl

Structure factors: contains datablock(s) V. DOI: 10.1107/S2056989018011544/su5452Vsup6.hkl

Structure factors: contains datablock(s) I Structure factors: contains datablock(s) I. DOI: 10.1107/S2056989018011544/su5452Isup2.hkl

Structure factors: contains datablock(s) VI. DOI: 10.1107/S2056989018011544/su5452VIsup7.hkl

Structure factors: contains datablock(s) VII. DOI: 10.1107/S2056989018011544/su5452VIIsup8.hkl

Structure factors: contains datablock(s) VIII. DOI: 10.1107/S2056989018011544/su5452VIIIsup9.hkl

Structure factors: contains datablock(s) IX. DOI: 10.1107/S2056989018011544/su5452IXsup10.hkl

Structure factors: contains datablock(s) X. DOI: 10.1107/S2056989018011544/su5452Xsup11.hkl

Structure factors: contains datablock(s) XI. DOI: 10.1107/S2056989018011544/su5452XIsup12.hkl

Supporting information file. DOI: 10.1107/S2056989018011544/su5452NUQVEBsup13.cml

Supporting information file. DOI: 10.1107/S2056989018011544/su5452QIQJADsup14.cml

Supporting information file. DOI: 10.1107/S2056989018011544/su5452SEDKETsup15.cml

Supporting information file. DOI: 10.1107/S2056989018011544/su5452TIYZIMsup16.cml

Supporting information file. DOI: 10.1107/S2056989018011544/su5452TUJPEVsup17.cml

Supporting information file. DOI: 10.1107/S2056989018011544/su5452VABZIJsup18.cml

Supporting information file. DOI: 10.1107/S2056989018011544/su5452WADXORsup19.cml

Supporting information file. DOI: 10.1107/S2056989018011544/su5452YAXPOEsup20.cml

Supporting information file. DOI: 10.1107/S2056989018011544/su5452sup21.pdf

CCDC references: 1063245, 1862187, 1862188, 1862189, 1862190, 1862191, 1862192, 1862193, 1862194, 1862195, 1862196, 1862197, 1862198

Additional supporting information:  crystallographic information; 3D view; checkCIF report

## Figures and Tables

**Table:** 

	DUJZAK	JEVNAA	LUDFUL	NUQVEB
Crystal data				
Chemical formula	[Ag(C_9_H_7_NO)_2_](C_7_H_3_N_2_O_7_)	[Zn(C_3_H_4_N_2_)_4_](C_7_H_3_N_2_O_7_)_2_	C_7_H_4_N_2_O_7_·C_12_H_8_N_2_	C_6_H_9_N_2_^+^·C_7_H_3_N_2_O_7_^−^
*M*_r_	625.30	791.93	408.33	336.27
Crystal system, space group	Monoclinic, *P*2_1_	Monoclinic, *C*2/*c*	Monoclinic, *P*2_1_/*a*	Triclinic, *P* 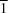
Temperature (K)	293	293	293	100
*a*, *b*, *c* (Å)	9.0154 (18), 7.6122 (15), 17.138 (3)	25.0809 (15), 6.7251 (4), 18.9145 (10)	14.8002 (15), 7.4029 (16), 16.0091 (16)	5.8673 (7), 8.0991 (9), 15.2437 (17)
α, β, γ (°)	90, 104.38 (3), 90	90, 97.658 (6), 90	90, 96.395 (8), 90	86.844 (3), 84.252 (3), 81.209 (3)
*V* (Å^3^)	1139.3 (4)	3161.9 (3)	1743.1 (5)	711.69 (14)
*Z*	2	4	4	2
Radiation type	Mo *K*α	Mo *K*α	Mo *K*α	Mo *K*α
μ (mm^−1^)	0.95	0.87	0.12	0.13
Crystal size (mm)	0.20 × 0.15 × 0.11	0.20 × 0.18 × 0.10	0.36 × 0.34 × 0.26	0.29 × 0.14 × 0.08

Data collection				
Diffractometer	Bruker SMART CCD area-detector	Bruker APEXII area-detector	Enraf–Nonius CAD-4	Bruker APEX DUO CCD area-detector
Absorption correction	–	Multi-scan (*SADABS*; Bruker, 1999)	–	Multi-scan (*SADABS*; Bruker, 2009)
*T*_min_, *T*_max_	–	0.846, 0.918	–	0.963, 0.990
No. of measured, independent and observed [*I* > 3σ(*I*)] reflections	10841, 4602, 4225	20634, 3635, 2152	8396, 4202, 1587	12709, 4943, 3677
*R*_int_	0.022	0.058	0.056	0.023
(sin θ/λ)_max_ (Å^−1^)	0.651	0.651	0.661	0.756

Refinement				
*R* factors and goodness of fit	*R*[*F* > 3σ(*F*)] = 0.023, *wR*(*F*) = 0.053, *S* = 1.34	*R*[*F* > 3σ(*F*)] = 0.036, *wR*(*F*) = 0.075, *S* = 1.23	*R*[*F* > 3σ(*F*)] = 0.044, *wR*(*F*) = 0.083, *S* = 1.08	*R*[*F* > 3σ(*F*)] = 0.042, *wR*(*F*) = 0.109, *S* = 2.06
No. of reflections	4602	3635	4202	4943
No. of parameters	356	244	274	222
No. of restraints	0	0	0	0
H-atom treatment	H atoms treated by a mixture of independent and constrained refinement	H atoms treated by a mixture of independent and constrained refinement	H atoms treated by a mixture of independent and constrained refinement	H atoms treated by a mixture of independent and constrained refinement
Δρ_max_, Δρ_min_ (e Å^−3^)	0.44, −0.30	0.23, −0.23	0.29, −0.31	0.40, −0.32
Absolute structure	1800 of Friedel pairs used in the refinement	–	–	–
Absolute structure parameter	0.004 (17)	–	–	–

**Table:** 

	QIQJAD	SAFGUD	SEDKET	TIYZIM
Crystal data				
Chemical formula	C_9_H_8_Cl_2_N_5_^+^·C_7_H_3_N_2_O_7_^−^·C_3_H_7_NO	[Ag(C_12_H_6_N_2_O_2_)](C_7_H_3_N_2_O_7_)	C_5_H_9_N_2_^+^·C_7_H_3_N_2_O_7_^−^	C_6_H_12_N_3_^+^·C_7_H_3_N_2_O_7_^−^
*M*_r_	557.31	755.36	324.26	353.30
Crystal system, space group	Triclinic, *P* 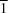	Monoclinic, *P*2_1_/*c*	Monoclinic, *P*2_1_	Triclinic, *P* 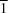
Temperature (K)	294	174	293	173
*a*, *b*, *c* (Å)	10.0227 (5), 10.5507 (5), 12.5359 (6)	11.757 (2), 18.297 (4), 13.223 (3)	8.1183 (7), 6.0636 (5), 14.1453 (11)	7.0109 (4), 10.6617 (8), 10.7454 (7)
α, β, γ (°)	81.858 (1), 71.888 (1), 70.009 (1)	90, 103.91 (3), 90	90, 91.904 (1), 90	93.075 (6), 95.863 (5), 104.944 (6)
*V* (Å^3^)	1183.1 (1)	2761.1 (11)	695.93 (10)	769.30 (9)
*Z*	2	4	2	2
Radiation type	Mo *K*α	Mo *K*α	Mo *K*α	Cu *K*α
μ (mm^−1^)	0.34	0.81	0.13	1.09
Crystal size (mm)	0.16 × 0.14 × 0.08	0.3 × 0.24 × 0.2	0.40 × 0.27 × 0.11	0.22 × 0.14 × 0.12

Data collection				
Diffractometer	Bruker SMART APEX CCD area-detector	Oxford Diffraction Gemini R Ultra	Bruker SMART CCD	Agilent Xcalibur (Eos, Gemini)
Absorption correction	Multi-scan (*SADABS*; Bruker, 2001)	Multi-scan (*SADABS*; Bruker, 2002)	Multi-scan (*SADABS*; Bruker, 2002)	Multi-scan (*CrysAlis PRO* and *CrysAlis RED*; Agilent, 2012)
*T*_min_, *T*_max_	0.93, 0.97	0.780, 0.910	0.959, 0.986	0.925, 1.000
No. of measured, independent and observed [*I* > 3σ(*I*)] reflections	13936, 5507, 4441	12726, 5013, 3100	3523, 2301, 1444	4664, 2953, 2426
*R*_int_	0.019	0.052	0.040	0.026
(sin θ/λ)_max_ (Å^−1^)	0.661	0.603	0.595	0.618

Refinement				
*R* factors and goodness of fit	*R*[*F* > 3σ(*F*)] = 0.056, *wR*(*F*) = 0.147, *S* = 3.41	*R*[*F* > 3σ(*F*)] = 0.062, *wR*(*F*) = 0.118, *S* = 1.64	*R*[*F* > 3σ(*F*)] = 0.041, *wR*(*F*) = 0.088, *S* = 1.16	*R*[*F* > 3σ(*F*)] = 0.041, *wR*(*F*) = 0.100, *S* = 1.64
No. of reflections	5507	5013	2301	2953
No. of parameters	340	444	212	229
No. of restraints	0	0	0	0
H-atom treatment	H atoms treated by a mixture of independent and constrained refinement	H-atom parameters constrained	H atoms treated by a mixture of independent and constrained refinement	H atoms treated by a mixture of independent and constrained refinement
Δρ_max_, Δρ_min_ (e Å^−3^)	0.80, −0.36	0.76, −0.63	0.11, −0.10	0.21, −0.18
Absolute structure	–	–	955 Friedel pairs used in the refinement	–
Absolute structure parameter	–	–	0.5	–

**Table:** 

	TUJPEV	(VABZIJ)	WADXOR	YAXPOE
Crystal data				
Chemical formula	C_10_H_12_N_3_O_3_S^+^·C_7_H_3_N_2_O_7_^−^	C_8_H_13_N_2_O^+^·C_7_H_3_N_2_O_7_^−^·H_2_O	C_9_H_17_N_2_^+^·C_7_H_3_N_2_O_7_^−^	C_26_H_29_N_2_^+^·C_7_H_3_N_2_O_7_^−^
*M*_r_	481.41	398.33	380.35	596.63
Crystal system, space group	Triclinic, *P* 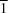	Triclinic, *P* 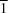	Monoclinic, *P*2_1_/*n*	Monoclinic, *P*2_1_/*c*
Temperature (K)	296	100	200	200
*a*, *b*, *c* (Å)	8.5551 (1), 10.5000 (2), 12.7576 (3)	6.6691 (3), 11.3831 (4), 12.2900 (5)	6.1537 (3), 19.1541 (14), 14.5527 (11)	14.5648 (3), 12.9374 (3), 16.1619 (3)
α, β, γ (°)	106.463 (1), 100.913 (1), 108.272 (1)	89.727 (2), 76.771 (2), 76.930 (2)	90, 98.343 (6), 90	90, 103.900 (1), 90
*V* (Å^3^)	993.72 (3)	883.62 (6)	1697.2 (2)	2956.22 (11)
*Z*	2	2	4	4
Radiation type	Mo *K*α	Mo *K*α	Mo *K*α	Mo *K*α
μ (mm^−1^)	0.23	0.13	0.12	0.10
Crystal size (mm)	0.20 × 0.20 × 0.16	0.52 × 0.13 × 0.10	0.30 × 0.13 × 0.10	0.51 × 0.26 × 0.17

Data collection				
Diffractometer	Bruker Kappa APEXII CCD	Bruker *SMART* APEXII CCD area-detector	Oxford Diffraction Gemini-S CCD-detector	Bruker APEXII CCD
Absorption correction	Multi-scan (*SADABS*; Bruker, 2004)	Multi-scan (*SADABS*; Bruker, 2009)	Multi-scan (*CrysAlis PRO*; Agilent, 2014)	Multi-scan (*SADABS*; Bruker, 2008)
*T*_min_, *T*_max_	0.955, 0.964	0.937, 0.987	0.920, 0.990	0.932, 1.000
No. of measured, independent and observed [*I* > 3σ(*I*)] reflections	24261, 6717, 4398	17014, 4061, 3042	7800, 3339, 1976	29552, 7344, 5724
*R*_int_	0.030	0.030	0.034	0.015
(sin θ/λ)_max_ (Å^−1^)	0.758	0.650	0.617	0.667

Refinement				
*R* factors and goodness of fit	*R*[*F* > 3σ(*F*)] = 0.044, *wR*(*F*) = 0.104, *S* = 1.95	*R*[*F* > 3σ(*F*)] = 0.038, *wR*(*F*) = 0.086, *S* = 1.77	*R*[*F* ^2^ > 2σ(*F* ^2^)] = 0.046, *wR*(*F* ^2^) = 0.095, *S* = 1.33	*R*[*F* > 3σ(*F*)] = 0.054, *wR*(*F*) = 0.190, *S* = 1.80
No. of reflections	6717	4061	3339	7344
No. of parameters	301	258	268	399
No. of restraints	0	0	2	0
H-atom treatment	H atoms treated by a mixture of independent and constrained refinement	H atoms treated by a mixture of independent and constrained refinement	H atoms treated by a mixture of independent and constrained refinement	H atoms treated by a mixture of independent and constrained refinement
Δρ_max_, Δρ_min_ (e Å^−3^)	0.31, −0.35	0.46, −0.23	0.36, −0.24	0.63, −0.28
Absolute structure	–	–	–	–
Absolute structure parameter	–	–	–	–

**Table 2:** Overview of the redetermined structures

REFCODE	Chemical name original/corrected if necessary
DUJZAK^*a*^	Bis(quinolin-8-ol)silver(I) 2-hy­droxy-3,5-di­nitro­benzoate
JEVNAA^*b*^	Tetra­kis(1*H*-imidazole-*N* ^3^)zinc(II) bis­(2-hy­droxy-3,5-di­nitro­benzoate / tetra­kis­(1*H*-imidazole-*N* ^3^)zinc(II) bis­(2-carb­oxy-4,6-di­nitro­phenolate)
LUDFUL^*c*^	1-Aza-8-azoniabi­cyclo­[5.4.0]undec-7-ene 2-hy­droxy-3,5-di­nitro­benzoate / phenazine 2-hy­droxy-3,5-di­nitro­benzoic acid
NUQVEB^*d*^	2-Amino-5-methyl­pyridinium 2-hy­droxy-3,5-di­nitro­benzoate) / 2-amino-5-methyl­pyridinium 2-hy­droxy-3,5-di­nitro­benzoate) (0.38) / 2-amino-5-methyl­pyridinium 2-carb­oxy-4,6-di­nitro­phenolate (0.62)
QIQJAD^*e*^	3,5-Di­amino-6-(2,3-di­chloro­phen­yl)-1,2,4-triazin-2-ium 3,5-di­nitro-2-hy­droxy­benzoate *N*,*N*-di­methyl­formamide solvate / 3,5-di­nitro-2-hy­droxy­benzoate (0.55) 2-carb­oxy-4,6-di­nitro­phenolate (0.45) *N*,*N*-di­methyl­formamide monosolvate / 3,5-di­amino-6-(2,3-di­chloro­phen­yl)-1,2,4-triazin-2-ium 3,5-di­nitro-2-hy­droxy­benzoate *N*,*N*-di­methyl­formamide monosolvate
SAFGUD^*f*^	Bis(1,10-phenanthroline-5,6-dione-2*N*,*N*′)silver(I) 2-hy­droxy-3,5-di­nitro­benzoate / bis(1,10-phenanthroline-5,6-dione-2*N*,*N*′)silver(I) 2-carb­oxy-4,6-di­nitro­phenolate
SEDKET^*g*^	3,5-Di­methyl­pyrazolium 2-carb­oxy-4,6-di­nitro­phenolate / 3,5-di­methyl­pyrazolium 2-hy­droxy-3,5-di­nitro­benzoate
TIYZI*M* ^*h*^	3-(1*H*-Imidazol-1-yl)propanaminium 2-carb­oxy-4,6-di­nitro­phenolate
TUJPEV^*i*^	4-[(5-methyl­isoxazol-3-yl)amino­sulfon­yl]anilinium 3,5-di­nitro­salicylate
VABZIJ^*j*^	2-Isopropyl-6-methyl-4-oxo-3,4-di­hydro­pyrimidin-1-ium 2-carb­oxy-4,6-di­nitro­phenolatemonohydrate
WADXO*R* ^*k*^	1-Aza-8-azoniabi­cyclo­[5.4.0]undec-7-ene 2-hy­droxy-3,5-di­nitro­benzoate / 2,3,4,6,7,8,9,10-octa­hydro­pyrimido[1,2-*a*]azepin-1-ium 2-hy­droxy-3,5-di­nitro­benzoate (0.73) / 2,3,4,6,7,8,9,10-octa­hydro­pyrimido[1,2-*a*]azepin-1-ium 2-carb­oxy-4,6–2-carb­oxy-4,6-di­nitro­phenolate (0.37)
YAXPOE^*l*^	4-(Di­phenyl­meth­yl)-1-(3-phenyl­prop-2-en-1-yl)piperazin-1-ium 2-carb­oxy-4,6-di­nitro­phenolate

Notes: (*a*) Zhang & Jian (2009 ▸); (*b*) Huang *et al.* (2007 ▸); (*c*) Senthil Kumar *et al.* (2002 ▸); (*d*) Hemamalini & Fun (2010*a* ▸); (*e*) Sridhar *et al.* (2013 ▸); (*f*) Wang *et al.* (2012 ▸); (*g*) Wei *et al.* (2012 ▸); (*h*) Yamuna *et al.* (2014 ▸); (*i*) Malathy *et al.* (2015 ▸); (*j*) Hemamalini & Fun (2010*b* ▸); (*k*) Smith & Lynch (2016 ▸); (*l*) Dayananda *et al.* (2012 ▸).

**Table 3:** Hydrogen bonds (Å, °) in the redetermined structures The upper entries for each hydrogen bond refer to refinement *Method 1*: fixed hydrogen-atom positions, which were obtained from the difference electron-density maps, and refined displacement parameters. The lower entries refer to refinement *Method 2*: refined hydrogen-atom positions and constrained displacement parameters.

*D*—H⋯*A*	*D*—H	H⋯*A*	*D*⋯*A*	*D*—H⋯*A*
DUJZAK				
O1—H1*aa*⋯O8	0.759 (2)	1.859 (2)	2.606 (3)	167.96 (14)
	0.97 (4)	1.64 (4)	2.603 (3)	175 (3)
O2—H2*aa*⋯O9	0.922 (2)	1.727 (2)	2.631 (3)	166.48 (15)
	0.75 (4)	1.90 (4)	2.636 (3)	165 (4)
O3—H3*b*⋯O9	1.040 (2)	1.495 (2)	2.481 (3)	155.88 (12)
	1.11 (4)	1.41 (4)	2.480 (3)	160 (3)
				
JEVNAA				
O2—H1*a*⋯O1	1.039 (2)	1.496 (2)	2.498 (2)	160.4 (1)
	0.89 (2)	1.65 (3)	2.503 (2)	160 (2)
N2—H2*a*⋯O3	0.967 (2)	1.890 (2)	2.838 (3)	165.9 (1)
	0.84 (2)	2.02 (2)	2.845 (3)	169 (2)
N4—H4*a*⋯O1^i^	0.943 (2)	1.924 (1)	2.784 (2)	150.6 (1)
	0.86 (2)	1.95 (2)	2.792 (2)	165 (2)
				
LUDFUL				
O3—H3*a*⋯O2	1.059 (1)	1.530 (1)	2.513 (2)	151.7 (1)
	1.06 (2)	1.51 (2)	2.516 (2)	156 (2)
O1—H1*a*⋯N3	1.163 (1)	1.416 (1)	2.552 (2)	163.2 (1)
	1.14 (2)	1.44 (2)	2.552 (2)	166 (2)
				
NUQVEB				
O7—H1*o*7⋯O1	0.919 (1)	1.531 (1)	2.4202 (12)	161.55 (6)
	1.14 (2)	1.31 (2)	2.4178 (12)	163 (2)
O1—H1*o*1⋯O7	0.931 (1)	1.513 (1)	2.4202 (12)	163.52 (6)
	1.31 (2)	1.14 (2)	2.4178 (12)	163 (2)
N2—H2*a*⋯O7^ii^	0.892 (1)	2.079 (1)	2.9655 (14)	172.84 (6)
	0.87 (1)	2.095 (14)	2.9674 (14)	176.5 (12)
N2—H2*b*⋯O1^iii^	0.846 (1)	2.165 (1)	2.8526 (14)	138.40 (6)
	0.88 (2)	2.146 (14)	2.852 (1)	137.3 (11)
N2—H2*b*⋯O2^iii^	0.846 (1)	2.413 (1)	3.1741 (14)	150.02 (6)
	0.88 (2)	2.384 (14)	3.1736 (15)	150.3 (11)
N1—H1⋯O6^ii^	0.898 (1)	1.783 (1)	2.6781 (13)	174.83 (6)
	0.90 (1)	1.784 (14)	2.6773 (14)	173.3 (13)
				
QIQJAD				
N3—H3*n*⋯O2	0.862 (2)	1.994 (2)	2.854 (2)	174.8 (1)
	0.81 (3)	2.05 (3)	2.854 (3)	175 (3)
N3—H4*n*⋯O8^iv^	0.863 (2)	2.059 (1)	2.921 (2)	176.9 (1)
	0.85 (2)	2.07 (2)	2.920 (2)	173 (3)
N2—H2*n*⋯O1	0.897 (2)	1.831 (2)	2.728 (2)	177.5 (1)
	0.81 (3)	1.93 (3)	2.731 (2)	171 (2)
N5—H5*n*⋯N4^v^	0.866 (1)	2.141 (1)	2.9992 (19)	171.1 (1)
	0.84 (2)	2.17 (2)	2.999 (2)	171 (2)
N5—H6*n*⋯O8^vi^	0.863 (2)	2.041 (2)	2.760 (2)	140.2 (1)
	0.78 (2)	2.12 (3)	2.764 (2)	141 (2)
O3—H3*o*⋯O1	0.926 (1)	1.562 (1)	2.4572 (18)	161.3 (1)
	0.99 (3)	1.49 (3)	2.4569 (19)	164 (3)
				
SAFGUD				
O8—H7⋯O7	1.155 (4)	1.346 (4)	2.462 (6)	159.6 (3)
	1.05 (7)	1.57 (7)	2.452 (7)	138 (6)
				
SEDKET				
O1—H2*a*⋯O2	1.22 (5)	1.34 (5)	2.476 (3)	149 (5)
	1.27 (3)	1.29 (3)	2.477 (3)	151 (3)
O2—H2*a*⋯O1	1.34 (5)	1.22 (5)	2.476 (3)	149 (5)
	1.29 (3)	1.27 (3)	2.477 (3)	151 (3)
N1—H1⋯O1^vii^	1.11 (5)	1.92 (5)	2.799 (4)	133 (3)
	0.99 (4)	2.00 (3)	2.804 (4)	137 (3)
N1—H1⋯O7^vii^	1.11 (5)	1.94 (5)	2.850 (4)	137 (3)
	0.99 (4)	2.03 (3)	2.855 (4)	140 (3)
N2—H2⋯O3	0.96 (3)	1.77 (3)	2.685 (4)	158 (3)
	0.99 (3)	1.75 (3)	2.684 (4)	157 (3)
				
TIYZIM				
O2*b*—H2*b*⋯O1*b*	0.982 (1)	1.516 (1)	2.4473 (16)	156.3 (1)
	1.02 (2)	1.48 (2)	2.4476 (16)	156 (2)
N3*a*—H3*aa*⋯N1*aa* ^viii^	0.904 (1)	1.932 (1)	2.797 (2)	159.6 (1)
	0.91	1.92	2.797 (2)	162
N3*a*—H3*ab*⋯O2*b* ^ix^	0.901 (1)	2.565 (1)	3.1297 (17)	121.4 (1)
	0.91	2.58	3.1298 (17)	120
N3*a*—H3*ab*⋯O2*b* ^ix^	0.901 (1)	2.565 (1)	3.1297 (17)	121.4 (1)
	0.91	2.58	3.1297 (18)	120
N3*a*—H3*ab*⋯O3*b* ^ix^	0.901 (1)	2.072 (1)	2.9537 (17)	165.8 (1)
	0.91	2.06	2.9542 (17)	165
N3*a*—H3*ac*⋯O1*b* ^*x*^	0.893 (1)	2.061 (1)	2.815 (2)	141.5 (1)
	0.91	2.03	2.815 (2)	144
N3*a*—H3*ac*⋯O7*b* ^*x*^	0.893 (1)	2.484 (1)	2.9712 (19)	114.7 (1)
	0.91	2.46	2.9706 (19)	116
				
TUJPEV				
O6—H6*a*⋯O5	1.184 (1)	1.295 (1)	2.4268 (16)	156.58 (6)
	1.24 (2)	1.21 (2)	2.4280 (17)	165.3 (14)
N1—H1*a*⋯O6^xi^	1.002 (1)	2.068 (1)	3.0655 (17)	173.55 (7)
	0.89	2.24	3.0694 (17)	155
N1—H1*b*⋯N3^v^	0.793 (1)	2.292 (1)	3.0393 (15)	157.3 (1)
	0.89	2.20	3.0382 (15)	157
N1—H1*c*⋯O4^v^	0.832 (2)	1.831 (1)	2.663 (2)	177.1 (1)
	0.89	1.77	2.660 (2)	175
N2—H2*a*⋯O5	0.970 (1)	1.844 (1)	2.7852 (15)	162.64 (9)
	0.827 (17)	1.986 (16)	2.7900 (16)	164.0 (18)
				
VABZIJ				
N3—H1*n*3⋯O6^*x*^	0.973 (1)	1.754 (1)	2.7182 (14)	170.48 (8)
	0.91 (1)	1.823 (14)	2.7214 (14)	170.8 (15)
N4—H1*n*4⋯O1*w*	0.909 (1)	1.840 (1)	2.7348 (15)	167.76 (8)
	0.91 (2)	1.833 (15)	2.7323 (16)	172.0 (15)
O1*w*—H2*w*1⋯O1^xii^	0.917 (1)	1.890 (1)	2.7886 (14)	166.21 (6)
	0.82 (2)	1.995 (19)	2.7906 (15)	162.7 (16)
O1*w*—H1*w*1—O3^iii^	0.915 (1)	2.040 (1)	2.9352 (14)	165.84 (7)
	0.89 (2)	2.064 (18)	2.9357 (15)	168.0 (17)
O7—H7⋯O1	1.019 (1)	1.433 (1)	2.4340 (13)	165.94 (7)
	0.96 (2)	1.505 (16)	2.4358 (13)	162.0 (16)
				
WADXOR				
N8*a*—H8*a*⋯O11*b*	0.960 (2)	1.933 (2)	2.864 (2)	162.83 (11)
	0.91 (2)	1.96 (2)	2.869 (2)	174.1 (17)
O11*b*—H21*b*⋯O21*b*	1.145 (2)	1.303 (6)	2.433 (6)	167.3 (3)
	1.07 (9)	1.48 (9)	2.430 (6)	145 (7)
O2*b*—H2*b*⋯O12*b*	1.103 (2)	1.385 (2)	2.471 (2)	166.81 (13)
	0.91 (3)	1.61 (3)	2.475 (3)	159 (3)
				
YAXPOE				
N1—H71⋯O1^iv^	0.945 (1)	1.954 (1)	2.813 (2)	150.01 (8)
	0.90 (2)	1.98 (2)	2.812 (2)	154.0 (19)
N1—H71⋯O2^iv^	0.945 (1)	2.302 (2)	3.032 (2)	133.62 (8)
	0.90 (2)	2.36 (2)	3.034 (2)	131.8 (17)
O7—H7⋯O1	0.924 (2)	1.668 (1)	2.505 (2)	148.96 (9)
	0.92 (3)	1.71 (3)	2.504 (2)	142 (2)

Symmetry codes: (i) −*x* + 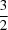, −*y* + 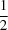, −*z* + 1; (ii) −*x* + 1, −*y* + 1, −*z*; (iii) −*x* + 1, −*y*, −*z*; (iv) −*x* + 1, −*y* + 1, −*z* + 1; (v) −*x* + 1, −*y* + 2, −*z* + 1; (vi) *x* − 1, *y* + 1, *z*; (vii) −*x* + 1, *y* + 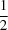, −*z* + 1; (viii) −*x*, −*y*, −*z*; (ix) *x* + 1, *y*, *z*; (*x*) −*x*, −*y* + 1, −*z* + 1; (xi) −*x*, −*y* + 2, −*z* + 1; (xii) *x*, *y* + 1, *z*.

**Table 4:** Overview of selected structures with different forms of the mol­ecules: 2-hy­droxy-3,5-di­nitro­benzoic acid (**I**); 2-hy­droxy-3,5-di­nitro­benzoate (**II**); 2-carb­oxy-4,6-di­nitro­phenolate (**III**); 3,5-di­nitro-2-oxidobenzoate (**IV**) The structures are ordered by ascending p*K*_*a*_ value of the base. The corresponding values of (*q*1 + *q*2), *D*13, *D*1, *D*2 and *D*5 (*cf*. Fig. 1 ▸) are also given.

	Refcode	Base and its form present in the structure	p*K*_*a*_	Δp*K*_*a*_	Type	(*q*1 + *q*2) (Å)	*D*13 (Å)	*D*1 (Å)	*D*2 (Å)	*D*5 (Å)	Remarks
1	GORXA*M* ^*a*^	1,4-dioxane	−3.9	−6.08	**I**	0.204 0.196	2.547 2.545	1.219 1.220	1.337 1.336	1.307 1.300	Two independent mol­ecules
2	GORXEQ^*a*^	1,4-dioxane	−3.9	−6.08	**I**	0.235	2.601	1.206	1.343	1.319	
3	GORXEQ01^*a*^	1,4-dioxane	−3.9	−6.08	**I**	0.197	2.531	1.222	1.346	1.302	
4	AJEBOG^*b*^	4-cyanopyridinium	1.92	−0.26	**III**	0.003	2.523	1.324	1.28	1.213	
5	ABULA*M* ^*c*^	2-aminoanilinium	<2	<-0.18	**III**	0.011	2.447	1.309	1.282	1.219	
6	PIDCAI^*c*^	2-aminoanilinium	<2	<-0.18	**III**	0.009	2.44	1.314	1.285	1.229	Wrongly attached hydrogen due to C=O distances. Originally determined as type **II** but it should be **III**.
7	PERBA*R* ^*d*^	3-carbamoylpyridinium	3.35	1.2	**II**	0.17	2.452	1.287	1.329	1.239	Wrongly attached hydrogen due to C=O distances. Originally determined as type **II** but it is probably **III**. Disorder present in the structure.
8	GIFMUE^*e*^	1-naphthylammonium	3.92	1.74	**III**	0.011	2.488	1.31	1.279	1.224	
9	MIPROS^*f*^	8-aminoquinolinium	3.95	1.77	**II**	0.072	2.408	1.278	1.300	1.237	The *bridging hydrogen* is situated about the centre.
10	ABUKUF^*g*^	4-chloroanilinium	3.98	1.80	**II**	0.094	2.435	1.276	1.297	1.242	
11	YIVHIW^*h*^	4-iodoanilinium	4.18	1.63	**II**	0.129	2.461	1.285	1.321	1.228	
12	GIFNUF^*i*^	1,10-phenanthrolinium	4.27	2.09	**II**	0.096	2.428	1.280	1.297	1.232	Determined as the type **III** but it is probably **II** (Fig. 1 ▸). The chemical name was correct.
13	FOXHAD^*j*^	2-(pyridin-2-yl)pyridinium	4.33	2.15	**II**	0.047	2.42	1.307	1.292	1.228	100 K; the reported hydrogen H3 is situated out of the plane formed by C⋯O bonds and is superficial.
14	KEZJIJ^*j*^	2-(pyridin-2-yl)pyridinium	4.33	2.15	**III**	0.07	2.422	1.293	1.296	1.231	C=O distances are about equal. The recalculation has shown that the *bridging hydrogen* is about the centre of the hydrogen bond, slightly closer to atom O2, which forms a shorter C=O bond.
15	KEZJIJ01^*j*^	2-(pyridin-2-yl)pyridinium	4.33	2.15	**III**	0.066	2.423	1.295	1.299	1.221	C=O distances are about equal, the hydrogen is attached to the O atom forming a shorter C=O bond.
16	FICXIZ^*k*^	cytosinium	4.60	2.42	**II**	0.098	2.423	1.285	1.310	1.234	The type according to the C=O distances should be **II**; the *bridging hydrogen* was wrongly attached.
17	ABUJUE^*l*^	anilinium	4.60	2.42	**II**	0.129	2.448	1.280	1.323	1.231	
18	ABUKO*Z* ^*m*^	4-fluoroanilinium	4.65	2.47	**II**	0.142	2.465	1.273	1.325	1.252	
19	GIFMOY^*n*^	quinolinium	4.85	2.67	**III**	0.05	2.414	1.294	1.285	1.235	The title mol­ecule has similarly long C=O distances.
20	ZAJHAT^*o*^	2-amminobenzoic acid	4.96	2.78	**II**	0.135	2.461	1.282	1.324	1.227	
21	AJEBIA^*p*^	pyridinium	5.23	3.05	**I** and **II**	0.142 0.163	2.458 2.582	1.250	1.308	1.257	Two independent mol­ecules
22	EGABOF^*q*^	2-methylquinolinium	5.71	3.53	**II**	0.285	2.411	1.207	1.359	1.244	Outlier
23	AJECEX01^*r*^	2,6-di­aminopyridin-1-ium	6.13	3.95	**II**	0.072 0.121	2.435 2.464	1.298 1.295	1.309 1.332	1.241 1.237	One of the title mol­ecules has similarly long C=O distances.
24	AJECIB^*s*^	2-aminopyrimidinium	6.82	4.64	**II**	0.114 0.145	2.466 2.473	1.277 1.270	1.308 1.323	1.241 1.238	
25	TUMWAB^*t*^	1*H*-imidazol-3-ium	6.95	4.77	**III**	−0.01	2.457	1.320	1.279	1.214	
26	LUMJOU^*u*^	hydrazinium	8.12	5.94	**III**	0.014	2.459	1.318	1.275	1.211	
27	SEDKET^*v*^	3,5-di­methylpyrazolium	9	6.82	**III**	0.037	2.481	1.300	1.282	1.224	
28	SEDKET^*v*^ (corrected)	3,5-di­methylpyrazolium	9	6.82	**II**	0.027	2.476	1.305	1.277	1.229	The *bridging hydrogen* after recalculation is closer to oxygen O1, which forms the shorter C=O bond (C12—O1).
29	LUDDET^*w*^	benzylammonium	9.33	7.15	**III**	0.002	2.483	1.305	1.269	1.218	
30	LUDDET01^*w*^	benzylammonium	9.33	7.15	**III**			1.311 1.311	1.275 1.279	1.217 1.219	
31	INELUI^*x*^	1-phenyl­ethylammonium	9.79	7.61	**III**	0.009 0.009	2.467 2.482	1.309 1.320	1.272 1.277	1.221 1.214	
32	MILLOI^*y*^	di­cyclo­hexylammonium	10.4	8.22	**III**	0.028	2.464	1.289	1.273	1.225	The C=O distances of the title mol­ecule are similar.
33	ACIFAT^*z*^	4-sulfamoylanilinium	10.6	8.42	**III**	0.028	2.462	1.315	1.287	1.209	
34	EGUTIJ^*aa*^	methylammonium	10.6	8.42	**III**	0.011	2.481	1.314	1.276	1.218	
35	EGUTOP^*bb*^	tri­ethylammonium	10.78	8.6	**II**	0.082	2.429	1.275	1.286	1.248	
36	EGUTOP01^*bb*^	tri­ethylammonium	10.78	8.6	**II**	0.072	2.419	1.275	1.288	1.242	
37	FOGZIL^*cc*^	di­ethylammonium	11.09	8.91	**III**	0.004	2.489	1.308	1.270	1.217	
38	XEBFA*M* ^*dd*^	piperidinium C_5_H_11_N	11.28	9.1	**II** and **IV**	0.078 0.061	2.586 2.736	1.219 1.234	1.278 1.253	1.255 1.271	One mol­ecule of DNSA (**I**) is fully ionized, the other is in form **II**.
39	YEJZAO^*ee*^	guanidinium	12.5	10.32	**II**	0.079	2.415	1.291	1.305	1.235	
40	YEJZAO01^*ee*^	guanidinium	12.5	10.32	**II**	0.073	2.415	1.292	1.300	1.239	

References for the p*K*_*a*_ values: (*a*) https://chemaxon.com/products/calculators-and-predictors#pka; (*b*) https://www.chemicalbook.com/ProductMSDSDetailCB0688145_EN.htm; (*c*) Dean (1987 ▸); (*d*) https://pubchem.ncbi.nlm.nih.gov/compound/nicotinamide#section=p*K*_a_; (*e*) https://labs.chem.ucsb.edu/zhang/liming/pdf/pKas_of_Organic_Acids_and_Bases.pdf; (*f*) http://binarystore.wiley.com/store/10.1002/jcc.23068/asset/supinfo/JCC_23068_sm_SuppInfo.pdf?v=1&s=e864a51d58b4cdc175f6b69c92ceddb546201e3b; (*g*) http://sites.chem.colostate.edu/diverdi/all_courses/CRC%20reference%20data/dissociation%20constants%20of%20organic%20acids%20and%20bases.pdf; (*h*) http://sites.chem.colostate.edu/diverdi/all_courses/CRC%20reference%20data/dissociation%20constants%20of%20organic%20acids%20and%20bases.pdf; (*i*) http://chemicalland21.com/specialtychem/finechem/1,10-PHENANTHROLINE.htm; (*j*) https://www.chemicalbook.com/ProductMSDSDetailCB5195697_EN.htm; (*k*) http://www.drugfuture.com/chemdata/cytosine.html; (*l*) https://pubchem.ncbi.nlm.nih.gov/compound/aniline#section=pKa; (*m*) http://sites.chem.colostate.edu/diverdi/all_courses/CRC%20reference%20data/dissociation%20constants%20of%20organic%20acids%20and%20bases.pdf; (*n*) Hosmane & Liebman (2009 ▸); (*o*) http://www.csun.edu/\~hcchm003/321/Ka.pdf; (*p*) https://pubchem.ncbi.nlm.nih.gov/compound/pyridine#section=Dissociation-Constants; (*q*) https://onlinelibrary.wiley.com/doi/pdf/10.1002/jcc.23068; (*r*) https://www.chemicalbook.com/ProductMSDSDetailCB0236195_EN.htm; (*s*) https://pubchem.ncbi.nlm.nih.gov/compound/2-amino­pyridine#section=Dissociation-Constants; (*t*) https://pubchem.ncbi.nlm.nih.gov/compound/imidazole#section=pKa; (*u*) http://evans.rc.fas.harvard.edu/pdf/evans_pKa_table.pdf; (*v*) https://www.chemicalbook.com/ProductMSDSDetailCB2707394_EN.htm; (*w*) https://pubchem.ncbi.nlm.nih.gov/compound/benzyl­amine#section=p*K*_a_; (*x*) https://www.drugbank.ca/drugs/DB04325; (*z*) https://pubchem.ncbi.nlm.nih.gov/compound/di­cyclo­hexyl­amine#section=Dissociation-Constants; (*z*) https://pubchem.ncbi.nlm.nih.gov/compound/sulfanilamide#section=Dissociation-Constants; (*aa*) https://pubchem.ncbi.nlm.nih.gov/compound/methyl­amine#section=p*K*_a_; (*ab*) https://pubchem.ncbi.nlm.nih.gov/compound/tri­ethyl­amine#section=Dissociation-Constants; (*ac*) https://pubchem.ncbi.nlm.nih.gov/compound/di­ethyl­amine#section=Dissociation-Constants; (*ad*) https://pubchem.ncbi.nlm.nih.gov/compound/piperidine#section=Dissociation-Constants; (*ae*) https://pubchem.ncbi.nlm.nih.gov/compound/guanidine#section=p*K*_a_.

References to publications with the chemical names of the determined compounds (original and corrected ones if necessary): (1) Senthil Kumar *et al.* (1999 ▸): 3,5-di­nitro­salicylic acid 1,4-dioxane solvate, 3,5-di­nitro­salicylic acid 1,4-dioxane (1:1)]; (2) Senthil Kumar *et al.* (1999 ▸): 3,5-di­nitro­salicylic acid 1,4-dioxane solvate, 3,5-di­nitro­salicylic acid 1,4-dioxane (2:1); (3) Senthil Kumar *et al.* (1999 ▸): 3,5-di­nitro­salicylic acid 1,4-dioxane solvate, 3,5-di­nitro­salicylic acid 1,4-dioxane (2:1); (4) Smith *et al.* (2003*a* ▸): 4-cyano­pyridinium 3,5-di­nitro­salicylate, 4-cyano­pyridinium 3,5-di­nitro­salicylate 2-carb­oxy-4,6-di­nitro­phenolate; (5) Smith *et al.* (2011 ▸): 2-amino­anilinium 2-carb­oxy-4,6-di­nitro­phenolate; (6) Khan *et al.* (2013 ▸): 2-amino­anilinium 2-hy­droxy-3,5-di­nitro­benzoate, 2-amino­anilinium 2-carb­oxy-4,6-di­nitro­phenolate; (7) Jin *et al.* (2013 ▸): 3-carbamoylpyridinium 2-carb­oxy-4,6-di­nitro­phenolate, 3-carbamoylpyridinium 2-hy­droxy-3,5-di­nitro­benzoate; (8) Smith *et al.* (2007 ▸): 1-naphthyl­ammonium 3,5-di­nitro­salicylate, 1-naphthyl­ammonium 2-carb­oxy-4,6-di­nitro­phenolate; (9) Smith *et al.* (2001*b* ▸): 8-amino­quinolinium 3,5-di­nitro­salicylate; (10) Smith *et al.* (2011 ▸): 4-chloro­anilinium 2-hy­droxy-3,5-di­nitro­benzoate; (11) Jones *et al.* (2014 ▸): (4-iodo­anilinium 2-hy­droxy-3,5-di­nitro­benzoate; (12) Smith *et al.* (2007 ▸): 1,10-Phenanthrolinium 3,5-di­nitro­salicylate; (13) Singh *et al.* (2014 ▸): 2-(pyridin-2-yl)pyridinium 2-hy­droxy-3,5-di­nitro­benzoate; (14) Song *et al.* (2007 ▸): 2,2′-bipyridinium 2-carb­oxy-4,6-di­nitro­phenolate; (15) Smith *et al.* (2007 ▸): 2,2′-bipyridinium 2-carb­oxy-4,6-di­nitro­phenolate; (16) Smith *et al.* (2005*a* ▸): cytosinium 3,5-di­nitro­salicylate, cytosinium 2-carb­oxy-4,6-di­nitro­phenolate; (17) Smith *et al.* (2011 ▸): anilinium 2-hy­droxy-3,5-di­nitro­benzoate; (18) Smith *et al.* (2011 ▸): 4-fluoro­anilinium 2-hy­droxy-3,5-di­nitro­benzoate; (19) Smith *et al.* (2007 ▸): quinolinium 3,5-di­nitro­salicylate, quinolinium 2-carb­oxy-4,6-di­nitro­phenolate; (20) Smith *et al.* (1995 ▸): 3,5-di­nitro­salicylic acid 2-amino­benzoic acid, 2-ammonium­benzoic acid 2-carb­oxy-4,6-di­nitro­phenolate; (21) Smith *et al.* (2003*a* ▸): pyridinium 3,5-di­nitro­salicylate 3,5-di­nitro­salicylic acid; (22) Zhang *et al.* (2014 ▸): 2-methyl­quinolinium 2-hy­droxy-3,5-di­nitro­benzoate; (23) Gao *et al.* (2015 ▸): 2,6-di­amino­pyridin-1-ium 2-hy­droxy-3,5-di­nitro­benzoate; (24) Smith *et al.* (2003*a* ▸): 2-amino­pyrimidinium 3,5-di­nitro­salicylate ethanol solvate, 2-amino­pyrimidinium 3,5-di­nitro­salicylate ethanol (2:2:1); (25) Jin *et al.* (2015*b* ▸): 1*H*-imidazol-3-ium 2-carb­oxy-4,6-di­nitro­phenolate; (26) Fu *et al.* (2015 ▸): hydrazinium 2-carb­oxy-4,6-di­nitro­phenolate; (27) Wei *et al.* (2012 ▸): (3,5-Di­methyl­pyrazolium 2-carb­oxy-4,6-di­nitro­phenolate); (28) this work: (3,5-di­methyl­pyrazolium 2-hy­droxy-3,5-di­nitro­benzoate; (29) Smith *et al.* (2002*b* ▸): benzyl­ammonium 3,5-di­nitro­salicylate, benzyl­ammonium 2-carb­oxy-4,6-di­nitro­phenolate; (30) Jin *et al.* (2015*a* ▸): benzyl­ammonium 2-carb­oxy-4,6-di­nitro­phenolate; (31) Smith *et al.* (2003*b* ▸): (*S*)-(−)-1-phenyl­ethyl­aminium 3,5-di­nitro­salicylate, (*S*)-(−)-1-phenyl­ethyl­aminium 2-carb­oxy-4,6-di­nitro­phenolate; (32) Ng *et al.* (2001 ▸): di­cyclo­hexyl­ammonium 2-carb­oxy-4,6-di­nitro­phenolate; (33) Smith *et al.* (2001*c* ▸): 4-ammonio­benzene­sulfonamide 3,5-di­nitro­salicylate, 4-ammonio­benzene­sulfonamide 2-carb­oxy-4,6-di­nitro­phenolate; (34) Smith *et al.* (2002*a* ▸): methyl­ammonium 3,5-di­nitro­salicylate, methyl­ammonium 2-carb­oxy-4,6-di­nitro­phenolate; (35) Smith *et al.* (2002*a* ▸): tri­ethyl­ammonium 3,5-di­nitro­salicylate; (36) Rajkumar & Chandramohan (2017 ▸): tri­ethyl­ammonium 2-hy­droxy-3,5-di­nitro­benzoate; (37) Smith *et al.* (2005*b* ▸): di­ethyl­ammonium 3,5-di­nitro­salicylate, di­ethyl­ammonium 2-carb­oxy-4,6-di­nitro­phenolate; (38) Smith *et al.* (2006 ▸): tris­(piperidinium) bis­(3,5-di­nitro­salicylate) monohydrate, tris­(piperidinium) 2-hy­droxy-3,5-di­nitro­benzoate 2-olate-3,5-di­nitro­benzoate monohydrate; (39) Smith *et al.* (2001*a* ▸): guanidinium 3,5-di­nitro­salicylate; (40) Fu *et al.* (2015 ▸): guanidinium 3,5-di­nitro­salicylate.

## supplementary crystallographic information

### Bis(quinolin-8-ol)silver(I) 2-hydroxy-3,5-dinitrobenzoate (DUJZAK) Crystal data

**Table:**

[Ag(C_9_H_7_NO)_2_](C_7_H_3_N_2_O_7_)	*F*(000) = 628
*M**_r_* = 625.30	*D*_x_ = 1.823 Mg m^−^^3^
Monoclinic, *P*2_1_	Mo *K*α radiation, λ = 0.71073 Å
Hall symbol: P 2yb	Cell parameters from 4356 reflections
*a* = 9.0154 (18) Å	θ = 3.6–27.6°
*b* = 7.6122 (15) Å	µ = 0.95 mm^−^^1^
*c* = 17.138 (3) Å	*T* = 293 K
β = 104.38 (3)°	Block, yellow
*V* = 1139.3 (4) Å^3^	0.20 × 0.15 × 0.11 mm
*Z* = 2	

### Bis(quinolin-8-ol)silver(I) 2-hydroxy-3,5-dinitrobenzoate (DUJZAK) Data collection

**Table:**

Bruker SMART CCD area-detector diffractometer	4225 reflections with *I* > 3σ(*I*)
Radiation source: fine-focus sealed tube	*R*_int_ = 0.022
Graphite monochromator	θ_max_ = 27.6°, θ_min_ = 3.6°
φ and ω scans	*h* = −11→11
10841 measured reflections	*k* = −9→8
4602 independent reflections	*l* = −22→22

### Bis(quinolin-8-ol)silver(I) 2-hydroxy-3,5-dinitrobenzoate (DUJZAK) Refinement

**Table:**

Refinement on *F*^2^	Secondary atom site location: difference Fourier map
*R*[*F* > 3σ(*F*)] = 0.023	Hydrogen site location: difference Fourier map
*wR*(*F*) = 0.053	H atoms treated by a mixture of independent and constrained refinement
*S* = 1.34	Weighting scheme based on measured s.u.'s *w* = 1/(σ^2^(*I*) + 0.0004*I*^2^)
4602 reflections	(Δ/σ)_max_ = 0.025
356 parameters	Δρ_max_ = 0.44 e Å^−^^3^
0 restraints	Δρ_min_ = −0.30 e Å^−^^3^
48 constraints	Absolute structure: 1800 of Friedel pairs used in the refinement
Primary atom site location: structure-invariant direct methods	Absolute structure parameter: 0.004 (17)

### Bis(quinolin-8-ol)silver(I) 2-hydroxy-3,5-dinitrobenzoate (DUJZAK) Special details

**Table:**

Geometry. All esds (except the esd in the dihedral angle between two l.s. planes) are estimated using the full covariance matrix. The cell esds are taken into account individually in the estimation of esds in distances, angles and torsion angles; correlations between esds in cell parameters are only used when they are defined by crystal symmetry. An approximate (isotropic) treatment of cell esds is used for estimating esds involving l.s. planes.
Refinement. Refinement of *F*^2^ against ALL reflections. The weighted *R*-factor *wR* and goodness of fit *S* are based on *F*^2^, conventional *R*-factors *R* are based on *F*, with *F* set to zero for negative *F*^2^. The threshold expression of *F*^2^ > σ(*F*^2^) is used only for calculating *R*-factors(gt) *etc*. and is not relevant to the choice of reflections for refinement. *R*-factors based on *F*^2^ are statistically about twice as large as those based on *F*, and *R*- factors based on ALL data will be even larger.Number of fixed parameters 9.

### Bis(quinolin-8-ol)silver(I) 2-hydroxy-3,5-dinitrobenzoate (DUJZAK) Fractional atomic coordinates and isotropic or equivalent isotropic displacement parameters (Å^2^)

**Table:**

	*x*	*y*	*z*	*U*_iso_*/*U*_eq_	
Ag1	0.062197 (18)	0.74282 (3)	0.668863 (11)	0.01868 (5)	
O1	−0.0993 (2)	0.4630 (2)	0.64021 (12)	0.0202 (6)	
O2	0.1741 (2)	0.5061 (2)	0.77196 (12)	0.0214 (6)	
N1	−0.1154 (2)	0.7667 (3)	0.55616 (13)	0.0177 (7)	
N2	0.2685 (2)	0.8371 (3)	0.75427 (14)	0.0163 (7)	
C1	−0.1280 (3)	0.9181 (4)	0.51463 (18)	0.0222 (9)	
H1a	−0.061241	1.009276	0.535386	0.0267*	
C2	−0.2362 (3)	0.9448 (4)	0.44205 (18)	0.0243 (9)	
H2a	−0.240923	1.051917	0.415439	0.0291*	
C3	−0.3354 (3)	0.8130 (4)	0.41016 (19)	0.0209 (9)	
H3a	−0.407848	0.829471	0.361612	0.0251*	
C4	−0.3270 (3)	0.6506 (4)	0.45167 (18)	0.0169 (8)	
C5	−0.4264 (3)	0.5079 (4)	0.42259 (17)	0.0209 (9)	
H5a	−0.500744	0.518344	0.37429	0.0251*	
C6	−0.4131 (3)	0.3554 (4)	0.46539 (17)	0.0217 (9)	
H6a	−0.477402	0.261662	0.445311	0.026*	
C7	−0.3036 (3)	0.3371 (3)	0.53948 (17)	0.0184 (8)	
H7a	−0.297965	0.232672	0.568204	0.0221*	
C8	−0.2057 (3)	0.4717 (3)	0.56954 (16)	0.0148 (8)	
C9	−0.2139 (3)	0.6331 (3)	0.52566 (16)	0.0141 (8)	
C10	0.3159 (3)	0.9993 (4)	0.74633 (17)	0.0188 (9)	
H10a	0.256892	1.069872	0.706108	0.0225*	
C11	0.4512 (3)	1.0703 (4)	0.79567 (18)	0.0220 (9)	
H11a	0.480885	1.184529	0.787703	0.0264*	
C12	0.5373 (3)	0.9693 (4)	0.85495 (18)	0.0210 (9)	
H12a	0.627346	1.01397	0.887796	0.0252*	
C13	0.4907 (3)	0.7960 (3)	0.86712 (16)	0.0171 (8)	
C14	0.5743 (3)	0.6845 (4)	0.92853 (17)	0.0200 (8)	
H14a	0.665083	0.723938	0.962749	0.024*	
C15	0.5225 (3)	0.5197 (4)	0.93771 (17)	0.0206 (8)	
H15a	0.577789	0.447807	0.978539	0.0247*	
C16	0.3863 (3)	0.4570 (3)	0.88620 (16)	0.0173 (8)	
H16a	0.35178	0.344669	0.8937	0.0208*	
C17	0.3043 (3)	0.5596 (3)	0.82528 (16)	0.0142 (8)	
C18	0.3541 (2)	0.7340 (5)	0.81443 (13)	0.0140 (6)	
O3	0.1402 (2)	−0.0134 (3)	0.92525 (13)	0.0259 (7)	
O4	−0.4152 (2)	−0.3755 (3)	0.69139 (13)	0.0284 (7)	
O5	−0.3271 (2)	−0.5961 (3)	0.76858 (13)	0.0271 (7)	
O6	0.1238 (2)	−0.5247 (2)	0.98352 (12)	0.0211 (6)	
O7	0.1679 (2)	−0.2659 (4)	1.03546 (11)	0.0286 (6)	
O8	−0.0981 (2)	0.1498 (2)	0.70180 (12)	0.0237 (7)	
O9	0.06657 (19)	0.2138 (2)	0.81885 (11)	0.0196 (6)	
N3	−0.3217 (2)	−0.4436 (3)	0.74764 (14)	0.0173 (7)	
N4	0.1088 (2)	−0.3645 (3)	0.98085 (13)	0.0152 (7)	
C19	−0.1970 (3)	−0.3339 (4)	0.79254 (18)	0.0130 (8)	
C20	−0.1027 (3)	−0.3992 (3)	0.86303 (15)	0.0126 (7)	
H20a	−0.114934	−0.512855	0.880295	0.0151*	
C21	0.0095 (2)	−0.2908 (3)	0.90660 (15)	0.0114 (8)	
C22	0.0326 (3)	−0.1188 (3)	0.88194 (16)	0.0130 (8)	
C23	−0.0605 (3)	−0.0603 (3)	0.80708 (16)	0.0129 (7)	
C24	−0.1770 (3)	−0.1678 (3)	0.76332 (17)	0.0132 (8)	
H24a	−0.240539	−0.128661	0.715089	0.0159*	
C25	−0.0307 (3)	0.1145 (3)	0.77245 (16)	0.0148 (8)	
H1aa	−0.091407	0.377732	0.663815	0.047 (14)*	
H2aa	0.145536	0.395004	0.783901	0.043 (11)*	
H3b	0.135279	0.095353	0.887993	0.17 (3)*	

### Bis(quinolin-8-ol)silver(I) 2-hydroxy-3,5-dinitrobenzoate (DUJZAK) Atomic displacement parameters (Å^2^)

**Table:**

	*U*^11^	*U*^22^	*U*^33^	*U*^12^	*U*^13^	*U*^23^
Ag1	0.01622 (8)	0.01722 (9)	0.01945 (10)	−0.00339 (11)	−0.00154 (6)	0.00024 (11)
O1	0.0219 (9)	0.0127 (9)	0.0199 (10)	−0.0063 (8)	−0.0065 (8)	0.0058 (8)
O2	0.0185 (9)	0.0173 (9)	0.0231 (11)	−0.0065 (8)	−0.0049 (8)	0.0030 (8)
N1	0.0190 (9)	0.0152 (14)	0.0181 (11)	−0.0003 (11)	0.0033 (8)	0.0008 (10)
N2	0.0145 (10)	0.0161 (11)	0.0187 (12)	−0.0018 (9)	0.0049 (9)	0.0005 (9)
C1	0.0254 (14)	0.0145 (12)	0.0263 (16)	−0.0042 (12)	0.0056 (12)	0.0044 (11)
C2	0.0325 (15)	0.0177 (13)	0.0239 (16)	0.0069 (13)	0.0093 (12)	0.0104 (11)
C3	0.0214 (13)	0.0247 (13)	0.0154 (15)	0.0059 (12)	0.0021 (12)	0.0023 (11)
C4	0.0136 (12)	0.0220 (14)	0.0150 (15)	0.0026 (11)	0.0031 (10)	0.0005 (12)
C5	0.0160 (12)	0.0289 (15)	0.0156 (14)	0.0005 (12)	−0.0002 (10)	−0.0035 (12)
C6	0.0156 (12)	0.0247 (14)	0.0217 (16)	−0.0092 (12)	−0.0009 (11)	−0.0062 (11)
C7	0.0179 (12)	0.0175 (14)	0.0187 (14)	−0.0039 (11)	0.0026 (10)	0.0018 (11)
C8	0.0139 (12)	0.0146 (12)	0.0140 (13)	0.0001 (11)	0.0000 (9)	−0.0001 (10)
C9	0.0147 (12)	0.0142 (12)	0.0132 (13)	−0.0002 (11)	0.0032 (9)	0.0016 (10)
C10	0.0208 (13)	0.0176 (13)	0.0198 (15)	−0.0021 (12)	0.0084 (11)	0.0021 (11)
C11	0.0259 (14)	0.0166 (13)	0.0254 (16)	−0.0085 (12)	0.0099 (12)	−0.0039 (11)
C12	0.0180 (12)	0.0226 (14)	0.0235 (15)	−0.0108 (12)	0.0074 (11)	−0.0101 (12)
C13	0.0138 (11)	0.0232 (14)	0.0156 (14)	−0.0032 (10)	0.0064 (10)	−0.0059 (10)
C14	0.0118 (11)	0.0285 (14)	0.0182 (15)	−0.0034 (11)	0.0010 (10)	−0.0071 (10)
C15	0.0143 (12)	0.0281 (15)	0.0175 (15)	0.0038 (12)	0.0002 (10)	0.0017 (11)
C16	0.0160 (12)	0.0146 (12)	0.0208 (15)	−0.0018 (11)	0.0035 (10)	−0.0001 (10)
C17	0.0107 (11)	0.0149 (12)	0.0165 (14)	−0.0020 (10)	0.0025 (9)	−0.0031 (10)
C18	0.0115 (9)	0.0160 (11)	0.0156 (11)	0.0014 (17)	0.0052 (8)	0.0001 (15)
O3	0.0215 (10)	0.0236 (11)	0.0279 (12)	−0.0066 (9)	−0.0029 (9)	0.0016 (9)
O4	0.0212 (10)	0.0283 (11)	0.0267 (12)	−0.0044 (9)	−0.0110 (8)	−0.0010 (9)
O5	0.0295 (10)	0.0195 (10)	0.0288 (12)	−0.0130 (9)	0.0004 (9)	0.0016 (9)
O6	0.0213 (9)	0.0158 (9)	0.0236 (11)	0.0028 (8)	0.0007 (8)	0.0059 (8)
O7	0.0340 (9)	0.0225 (9)	0.0194 (9)	0.0057 (15)	−0.0121 (7)	−0.0015 (13)
O8	0.0304 (11)	0.0156 (10)	0.0204 (11)	−0.0061 (9)	−0.0028 (8)	0.0051 (8)
O9	0.0209 (8)	0.0129 (12)	0.0223 (10)	−0.0061 (8)	0.0003 (7)	0.0009 (8)
N3	0.0141 (10)	0.0190 (11)	0.0169 (12)	−0.0064 (10)	0.0001 (9)	−0.0044 (9)
N4	0.0115 (10)	0.0169 (11)	0.0154 (12)	0.0012 (9)	−0.0003 (8)	0.0025 (9)
C19	0.0080 (11)	0.0164 (12)	0.0135 (15)	−0.0043 (10)	0.0009 (10)	−0.0044 (11)
C20	0.0158 (12)	0.0082 (11)	0.0137 (13)	−0.0009 (10)	0.0036 (9)	0.0004 (9)
C21	0.0098 (9)	0.0122 (17)	0.0103 (11)	0.0048 (10)	−0.0012 (8)	0.0021 (9)
C22	0.0086 (11)	0.0150 (13)	0.0147 (14)	−0.0009 (10)	0.0015 (9)	−0.0033 (10)
C23	0.0133 (11)	0.0110 (12)	0.0134 (13)	−0.0001 (10)	0.0015 (9)	−0.0007 (9)
C24	0.0120 (12)	0.0135 (13)	0.0142 (15)	0.0018 (11)	0.0033 (10)	0.0004 (11)
C25	0.0153 (12)	0.0109 (11)	0.0177 (14)	0.0006 (10)	0.0029 (10)	0.0010 (10)

### Bis(quinolin-8-ol)silver(I) 2-hydroxy-3,5-dinitrobenzoate (DUJZAK) Geometric parameters (Å, º)

**Table:**

O1—C8	1.347 (3)	C13—C14	1.415 (4)
O1—H1aa	0.7585 (19)	C13—C18	1.415 (3)
O2—C17	1.359 (3)	C14—H14a	0.93
O2—H2aa	0.922 (2)	C14—C15	1.361 (4)
N1—C1	1.344 (4)	C15—H15a	0.93
N1—C9	1.365 (3)	C15—C16	1.406 (3)
N2—C10	1.325 (4)	C16—H16a	0.93
N2—C18	1.371 (4)	C16—C17	1.365 (4)
C1—H1a	0.93	C17—C18	1.429 (5)
C1—C2	1.392 (4)	O3—C22	1.333 (3)
C2—H2a	0.93	O3—H3b	1.040 (2)
C2—C3	1.364 (4)	O4—N3	1.227 (3)
C3—H3a	0.93	O5—N3	1.219 (3)
C3—C4	1.419 (4)	O6—N4	1.226 (3)
C4—C5	1.418 (4)	O7—N4	1.215 (3)
C4—C9	1.423 (4)	O8—C25	1.242 (3)
C5—H5a	0.93	O9—C25	1.275 (3)
C5—C6	1.362 (4)	O9—H3b	1.4952 (19)
C6—H6a	0.93	N3—C19	1.458 (3)
C6—C7	1.408 (4)	N4—C21	1.473 (3)
C7—H7a	0.93	C19—C20	1.385 (4)
C7—C8	1.367 (4)	C19—C24	1.388 (4)
C8—C9	1.433 (4)	C20—H20a	0.93
C10—H10a	0.93	C20—C21	1.373 (3)
C10—C11	1.408 (4)	C21—C22	1.408 (4)
C11—H11a	0.93	C22—C23	1.419 (3)
C11—C12	1.354 (4)	C23—C24	1.394 (3)
C12—H12a	0.93	C23—C25	1.508 (4)
C12—C13	1.416 (4)	C24—H24a	0.93
			
C8—O1—H1aa	118.2 (2)	C13—C14—C15	120.2 (2)
C17—O2—H2aa	111.60 (19)	H14a—C14—C15	119.88
C1—N1—C9	118.3 (2)	C14—C15—H15a	119.61
C10—N2—C18	118.4 (2)	C14—C15—C16	120.8 (2)
N1—C1—H1a	118.43	H15a—C15—C16	119.61
N1—C1—C2	123.1 (2)	C15—C16—H16a	119.71
H1a—C1—C2	118.43	C15—C16—C17	120.6 (2)
C1—C2—H2a	120.19	H16a—C16—C17	119.71
C1—C2—C3	119.6 (3)	O2—C17—C16	123.8 (2)
H2a—C2—C3	120.19	O2—C17—C18	116.0 (2)
C2—C3—H3a	120.26	C16—C17—C18	120.2 (2)
C2—C3—C4	119.5 (2)	N2—C18—C13	121.8 (3)
H3a—C3—C4	120.27	N2—C18—C17	119.6 (2)
C3—C4—C5	122.8 (2)	C13—C18—C17	118.6 (2)
C3—C4—C9	117.8 (2)	C22—O3—H3b	102.89 (19)
C5—C4—C9	119.5 (2)	C25—O9—H3b	102.84 (17)
C4—C5—H5a	120	O4—N3—O5	124.3 (2)
C4—C5—C6	120.0 (2)	O4—N3—C19	117.5 (2)
H5a—C5—C6	120	O5—N3—C19	118.2 (2)
C5—C6—H6a	119.4	O6—N4—O7	124.2 (2)
C5—C6—C7	121.2 (2)	O6—N4—C21	116.66 (19)
H6a—C6—C7	119.4	O7—N4—C21	119.1 (2)
C6—C7—H7a	119.72	N3—C19—C20	118.7 (2)
C6—C7—C8	120.6 (2)	N3—C19—C24	118.9 (2)
H7a—C7—C8	119.72	C20—C19—C24	122.3 (2)
O1—C8—C7	123.5 (2)	C19—C20—H20a	121.04
O1—C8—C9	116.5 (2)	C19—C20—C21	117.9 (2)
C7—C8—C9	120.0 (2)	H20a—C20—C21	121.04
N1—C9—C4	121.7 (2)	N4—C21—C20	116.7 (2)
N1—C9—C8	119.6 (2)	N4—C21—C22	120.67 (19)
C4—C9—C8	118.7 (2)	C20—C21—C22	122.7 (2)
N2—C10—H10a	118.33	O3—C22—C21	122.3 (2)
N2—C10—C11	123.3 (2)	O3—C22—C23	120.0 (2)
H10a—C10—C11	118.33	C21—C22—C23	117.7 (2)
C10—C11—H11a	120.58	C22—C23—C24	120.0 (2)
C10—C11—C12	118.8 (3)	C22—C23—C25	120.6 (2)
H11a—C11—C12	120.58	C24—C23—C25	119.4 (2)
C11—C12—H12a	119.87	C19—C24—C23	119.2 (2)
C11—C12—C13	120.3 (2)	C19—C24—H24a	120.38
H12a—C12—C13	119.87	C23—C24—H24a	120.38
C12—C13—C14	123.1 (2)	O8—C25—O9	125.0 (2)
C12—C13—C18	117.4 (2)	O8—C25—C23	118.9 (2)
C14—C13—C18	119.6 (3)	O9—C25—C23	116.1 (2)
C13—C14—H14a	119.88	O3—H3b—O9	155.88 (12)

### Bis(quinolin-8-ol)silver(I) 2-hydroxy-3,5-dinitrobenzoate (DUJZAK) Hydrogen-bond geometry (Å, º)

**Table:**

*D*—H···*A*	*D*—H	H···*A*	*D*···*A*	*D*—H···*A*
C11—H11*a*···O5^i^	0.93	2.48	3.335 (4)	152
O1—H1*aa*···O8	0.7585 (19)	1.859 (2)	2.606 (3)	167.96 (14)
O2—H2*aa*···O9	0.922 (2)	1.727 (2)	2.631 (3)	166.48 (15)
O3—H3*b*···O9	1.040 (2)	1.4952 (19)	2.481 (3)	155.88 (12)

Symmetry code: (i) *x*+1, *y*+2, *z*.

### Tetrakis(1H-imidazole-κN3)zinc(II) bis(2-hydroxy-3,5-dinitrobenzoate) (JEVNAA) Crystal data

**Table:**

[Zn(C_3_H_4_N_2_)_4_](C_7_H_3_N_2_O_7_)_2_	*F*(000) = 1616
*M**_r_* = 791.93	*D*_x_ = 1.664 Mg m^−^^3^
Monoclinic, *C*2/*c*	Mo *K*α radiation, λ = 0.71073 Å
Hall symbol: -C 2yc	Cell parameters from 3242 reflections
*a* = 25.0809 (15) Å	θ = 2.1–26.9°
*b* = 6.7251 (4) Å	µ = 0.87 mm^−^^1^
*c* = 18.9145 (10) Å	*T* = 293 K
β = 97.658 (6)°	Platelet, yellow
*V* = 3161.9 (3) Å^3^	0.20 × 0.18 × 0.10 mm
*Z* = 4	

### Tetrakis(1H-imidazole-κN3)zinc(II) bis(2-hydroxy-3,5-dinitrobenzoate) (JEVNAA) Data collection

**Table:**

Bruker APEX-II area-detector diffractometer	3635 independent reflections
Radiation source: fine-focus sealed tube	2152 reflections with *I* > 3σ(*I*)
Graphite monochromator	*R*_int_ = 0.058
φ and ω scans	θ_max_ = 27.6°, θ_min_ = 1.6°
Absorption correction: multi-scan (SADABS; Bruker, 1999)	*h* = −32→31
*T*_min_ = 0.846, *T*_max_ = 0.918	*k* = −8→8
20634 measured reflections	*l* = −24→24

### Tetrakis(1H-imidazole-κN3)zinc(II) bis(2-hydroxy-3,5-dinitrobenzoate) (JEVNAA) Refinement

**Table:**

Refinement on *F*^2^	Secondary atom site location: difference Fourier map
Least-squares matrix: full	Hydrogen site location: difference Fourier map
*R*[*F* > 3σ(*F*)] = 0.036	H atoms treated by a mixture of independent and constrained refinement
*wR*(*F*) = 0.075	Weighting scheme based on measured s.u.'s *w* = 1/(σ^2^(*I*) + 0.0004*I*^2^)
*S* = 1.23	(Δ/σ)_max_ = 0.007
3635 reflections	Δρ_max_ = 0.23 e Å^−^^3^
244 parameters	Δρ_min_ = −0.23 e Å^−^^3^
0 restraints	Extinction correction: B-C type 1 Lorentzian isotropic (Becker & Coppens, 1974)
32 constraints	Extinction coefficient: 1400 (500)
Primary atom site location: structure-invariant direct methods	

### Tetrakis(1H-imidazole-κN3)zinc(II) bis(2-hydroxy-3,5-dinitrobenzoate) (JEVNAA) Special details

**Table:**

Geometry. All esds (except the esd in the dihedral angle between two l.s. planes) are estimated using the full covariance matrix. The cell esds are taken into account individually in the estimation of esds in distances, angles and torsion angles; correlations between esds in cell parameters are only used when they are defined by crystal symmetry. An approximate (isotropic) treatment of cell esds is used for estimating esds involving l.s. planes.
Refinement. Refinement of F^2^ against ALL reflections. The weighted R-factor wR and goodness of fit S are based on F^2^, conventional R-factors R are based on F, with F set to zero for negative F^2^. The threshold expression of F^2^ > 2sigma(F^2^) is used only for calculating R-factors(gt) etc. and is not relevant to the choice of reflections for refinement. R-factors based on F^2^ are statistically about twice as large as those based on F, and R- factors based on ALL data will be even larger. Number of fixed parameters: 9

### Tetrakis(1H-imidazole-κN3)zinc(II) bis(2-hydroxy-3,5-dinitrobenzoate) (JEVNAA) Fractional atomic coordinates and isotropic or equivalent isotropic displacement parameters (Å^2^)

**Table:**

	*x*	*y*	*z*	*U*_iso_*/*U*_eq_	
C1	0.47960 (10)	0.4200 (3)	0.62639 (13)	0.0460 (10)	
H1	0.444004	0.398202	0.633096	0.0552*	
C2	0.50386 (10)	0.3369 (4)	0.57533 (13)	0.0484 (10)	
H2	0.488745	0.248295	0.540565	0.0581*	
C3	0.56018 (9)	0.5299 (3)	0.63970 (12)	0.0401 (9)	
H3	0.591591	0.597819	0.656666	0.0481*	
C4	0.59325 (9)	0.9065 (4)	0.83991 (12)	0.0404 (9)	
H4	0.591722	0.823229	0.87885	0.0485*	
N4	0.62879 (7)	1.0508 (3)	0.83789 (11)	0.0471 (8)	
C6	0.57665 (9)	1.0451 (4)	0.73735 (13)	0.0444 (9)	
H6	0.560952	1.074705	0.691199	0.0533*	
C7	0.73999 (9)	0.4553 (3)	0.34225 (11)	0.0329 (8)	
C8	0.79245 (9)	0.5059 (3)	0.36676 (12)	0.0342 (8)	
H8	0.817639	0.51762	0.335092	0.0411*	
C9	0.80717 (8)	0.5388 (3)	0.43803 (12)	0.0311 (8)	
C10	0.77048 (8)	0.5210 (3)	0.48978 (12)	0.0302 (8)	
C11	0.71702 (8)	0.4593 (3)	0.46099 (11)	0.0282 (7)	
C12	0.70286 (9)	0.4300 (3)	0.38898 (12)	0.0323 (8)	
H12	0.667845	0.392676	0.371656	0.0388*	
C13	0.67545 (9)	0.4249 (3)	0.50834 (12)	0.0333 (8)	
N1	0.51487 (7)	0.5429 (3)	0.66784 (9)	0.0373 (7)	
N2	0.55523 (8)	0.4075 (3)	0.58408 (10)	0.0438 (8)	
N3	0.56024 (7)	0.8957 (3)	0.77961 (9)	0.0354 (7)	
C5	0.61854 (9)	1.1406 (4)	0.77303 (14)	0.0479 (10)	
H5	0.637064	1.247402	0.75675	0.0575*	
N5	0.72386 (8)	0.4225 (3)	0.26644 (10)	0.0427 (8)	
N6	0.86351 (7)	0.5897 (3)	0.46068 (11)	0.0403 (8)	
O1	0.78238 (6)	0.5518 (2)	0.55681 (8)	0.0378 (6)	
O2	0.68832 (6)	0.4695 (2)	0.57640 (8)	0.0420 (6)	
O3	0.63104 (6)	0.3579 (2)	0.48669 (8)	0.0426 (6)	
O4	0.67630 (7)	0.3835 (3)	0.24643 (8)	0.0575 (7)	
O5	0.75772 (7)	0.4340 (3)	0.22570 (9)	0.0662 (8)	
O6	0.89634 (7)	0.5475 (3)	0.42013 (9)	0.0658 (8)	
O7	0.87622 (6)	0.6730 (3)	0.51757 (9)	0.0568 (7)	
Zn1	0.5	0.70908 (6)	0.75	0.03866 (15)	
H1a	0.728641	0.508268	0.579335	0.108 (11)*	
H2a	0.580668	0.367714	0.552562	0.088 (9)*	
H4a	0.657593	1.098579	0.870594	0.106 (11)*	

### Tetrakis(1H-imidazole-κN3)zinc(II) bis(2-hydroxy-3,5-dinitrobenzoate) (JEVNAA) Atomic displacement parameters (Å^2^)

**Table:**

	*U*^11^	*U*^22^	*U*^33^	*U*^12^	*U*^13^	*U*^23^
C1	0.0323 (14)	0.0558 (17)	0.0507 (17)	−0.0069 (12)	0.0086 (12)	−0.0071 (13)
C2	0.0460 (16)	0.0536 (18)	0.0448 (17)	−0.0064 (13)	0.0033 (12)	−0.0116 (13)
C3	0.0327 (13)	0.0485 (16)	0.0396 (16)	−0.0053 (11)	0.0071 (11)	0.0037 (12)
C4	0.0366 (14)	0.0491 (16)	0.0343 (15)	0.0066 (12)	0.0004 (11)	0.0062 (12)
N4	0.0332 (12)	0.0568 (15)	0.0484 (15)	−0.0016 (10)	−0.0048 (10)	−0.0044 (11)
C6	0.0416 (15)	0.0568 (17)	0.0336 (15)	−0.0015 (12)	0.0004 (12)	0.0077 (13)
C7	0.0344 (13)	0.0369 (14)	0.0264 (14)	0.0024 (10)	0.0002 (10)	−0.0008 (10)
C8	0.0332 (13)	0.0366 (14)	0.0337 (15)	0.0009 (10)	0.0073 (10)	0.0030 (10)
C9	0.0254 (12)	0.0287 (13)	0.0382 (15)	−0.0031 (9)	0.0003 (10)	−0.0001 (10)
C10	0.0338 (13)	0.0219 (12)	0.0342 (14)	0.0035 (9)	0.0013 (10)	0.0006 (10)
C11	0.0288 (12)	0.0245 (12)	0.0310 (14)	0.0011 (9)	0.0030 (10)	−0.0013 (10)
C12	0.0281 (12)	0.0306 (14)	0.0370 (15)	0.0009 (9)	−0.0002 (10)	0.0004 (10)
C13	0.0338 (13)	0.0312 (14)	0.0356 (15)	0.0037 (10)	0.0067 (11)	−0.0026 (10)
N1	0.0341 (11)	0.0433 (13)	0.0357 (12)	−0.0029 (9)	0.0086 (9)	−0.0001 (9)
N2	0.0437 (12)	0.0526 (14)	0.0371 (13)	0.0034 (10)	0.0126 (10)	−0.0035 (10)
N3	0.0321 (10)	0.0442 (12)	0.0288 (11)	0.0016 (9)	0.0003 (9)	0.0042 (9)
C5	0.0393 (15)	0.0540 (18)	0.0499 (18)	−0.0090 (12)	0.0040 (12)	0.0069 (14)
N5	0.0414 (13)	0.0517 (14)	0.0338 (13)	0.0032 (10)	0.0004 (10)	−0.0005 (10)
N6	0.0327 (11)	0.0435 (13)	0.0438 (14)	−0.0038 (9)	0.0023 (10)	0.0059 (10)
O1	0.0362 (9)	0.0445 (10)	0.0308 (10)	−0.0009 (7)	−0.0026 (7)	−0.0054 (7)
O2	0.0399 (10)	0.0552 (11)	0.0313 (10)	−0.0055 (8)	0.0056 (7)	−0.0066 (8)
O3	0.0325 (9)	0.0561 (11)	0.0400 (10)	−0.0091 (8)	0.0079 (7)	−0.0084 (8)
O4	0.0395 (10)	0.0890 (14)	0.0408 (11)	−0.0047 (9)	−0.0063 (8)	−0.0048 (9)
O5	0.0499 (11)	0.1146 (17)	0.0361 (11)	−0.0057 (10)	0.0133 (9)	−0.0036 (10)
O6	0.0351 (10)	0.1012 (16)	0.0635 (13)	−0.0075 (10)	0.0156 (9)	−0.0121 (11)
O7	0.0430 (10)	0.0799 (14)	0.0453 (11)	−0.0182 (9)	−0.0029 (8)	−0.0108 (10)
Zn1	0.0355 (2)	0.0455 (3)	0.0352 (3)	0	0.00566 (17)	0

### Tetrakis(1H-imidazole-κN3)zinc(II) bis(2-hydroxy-3,5-dinitrobenzoate) (JEVNAA) Geometric parameters (Å, º)

**Table:**

C1—H1	0.93	C8—H8	0.93
C1—C2	1.331 (4)	C8—C9	1.367 (3)
C1—N1	1.377 (3)	C9—C10	1.435 (3)
C2—H2	0.93	C9—N6	1.462 (3)
C2—N2	1.362 (3)	C10—C11	1.440 (3)
C3—H3	0.93	C10—O1	1.280 (3)
C3—N1	1.320 (3)	C11—C12	1.375 (3)
C3—N2	1.328 (3)	C11—C13	1.480 (3)
C4—H4	0.93	C12—H12	0.93
C4—N4	1.322 (3)	C13—O2	1.319 (3)
C4—N3	1.319 (3)	C13—O3	1.220 (3)
N4—C5	1.361 (3)	N2—H2a	0.967 (2)
N4—H4a	0.9427 (18)	C5—H5	0.93
C6—H6	0.93	N5—O4	1.231 (3)
C6—N3	1.381 (3)	N5—O5	1.223 (3)
C6—C5	1.335 (3)	N6—O6	1.231 (3)
C7—C8	1.378 (3)	N6—O7	1.217 (3)
C7—C12	1.377 (3)	O1—H1a	1.4955 (15)
C7—N5	1.454 (3)	O2—H1a	1.0386 (15)
			
H1—C1—C2	124.94	C9—C10—O1	125.18 (18)
H1—C1—N1	124.94	C11—C10—O1	120.2 (2)
C2—C1—N1	110.1 (2)	C10—C11—C12	121.4 (2)
C1—C2—H2	126.81	C10—C11—C13	120.78 (19)
C1—C2—N2	106.4 (2)	C12—C11—C13	117.85 (18)
H2—C2—N2	126.81	C7—C12—C11	120.69 (19)
H3—C3—N1	124.28	C7—C12—H12	119.65
H3—C3—N2	124.28	C11—C12—H12	119.66
N1—C3—N2	111.44 (19)	C11—C13—O2	117.03 (18)
H4—C4—N4	124.35	C11—C13—O3	122.6 (2)
H4—C4—N3	124.35	O2—C13—O3	120.3 (2)
N4—C4—N3	111.3 (2)	C1—N1—C3	104.66 (19)
C4—N4—C5	107.74 (19)	C2—N2—C3	107.4 (2)
C4—N4—H4a	133.8 (2)	C2—N2—H2a	121.3 (2)
C5—N4—H4a	118.5 (2)	C3—N2—H2a	131.3 (2)
H6—C6—N3	125.29	C4—N3—C6	105.04 (18)
H6—C6—C5	125.29	N4—C5—C6	106.5 (2)
N3—C6—C5	109.4 (2)	N4—C5—H5	126.75
C8—C7—C12	120.8 (2)	C6—C5—H5	126.75
C8—C7—N5	119.8 (2)	C7—N5—O4	117.82 (19)
C12—C7—N5	119.40 (19)	C7—N5—O5	119.10 (18)
C7—C8—H8	120.27	O4—N5—O5	123.08 (19)
C7—C8—C9	119.5 (2)	C9—N6—O6	117.66 (19)
H8—C8—C9	120.27	C9—N6—O7	119.73 (19)
C8—C9—C10	123.06 (18)	O6—N6—O7	122.61 (18)
C8—C9—N6	116.8 (2)	C10—O1—H1a	98.67 (13)
C10—C9—N6	120.16 (19)	C13—O2—H1a	102.66 (16)
C9—C10—C11	114.56 (19)	O1—H1a—O2	160.37 (10)

### Tetrakis(1H-imidazole-κN3)zinc(II) bis(2-hydroxy-3,5-dinitrobenzoate) (JEVNAA) Hydrogen-bond geometry (Å, º)

**Table:**

*D*—H···*A*	*D*—H	H···*A*	*D*···*A*	*D*—H···*A*
C4—H4···O3^i^	0.93	2.47	3.327 (3)	154
O2—H1*a*···C10	1.0386 (15)	2.110 (2)	2.820 (3)	123.5 (1)
O2—H1*a*···O1	1.0386 (15)	1.4955 (15)	2.498 (2)	160.4 (1)
N2—H2*a*···O3	0.967 (2)	1.8902 (16)	2.838 (3)	165.87 (12)
N4—H4*a*···O1^ii^	0.9427 (18)	1.9236 (14)	2.784 (2)	150.60 (13)
N4—H4*a*···O7^ii^	0.9427 (18)	2.4336 (18)	2.873 (3)	108.36 (13)

Symmetry codes: (i) *x*, −*y*+1, *z*+1/2; (ii) −*x*+3/2, *y*+1/2, −*z*+3/2.

### 3,5-Dinitrosalicylic acid–phenazine (1/1) (LUDFUL) Crystal data

**Table:**

C_7_H_4_N_2_O_7_·C_12_H_8_N_2_	*D*_x_ = 1.556 Mg m^−^^3^
*M**_r_* = 408.33	Melting point: 471 K
Monoclinic, *P*2_1_/*a*	Mo *K*α radiation, λ = 0.71073 Å
*a* = 14.8002 (15) Å	Cell parameters from 25 reflections
*b* = 7.4029 (16) Å	θ = 5–12°
*c* = 16.0091 (16) Å	µ = 0.12 mm^−^^1^
β = 96.395 (8)°	*T* = 293 K
*V* = 1743.1 (5) Å^3^	Rhombic, yellow
*Z* = 4	0.36 × 0.34 × 0.26 mm
*F*(000) = 840	

### 3,5-Dinitrosalicylic acid–phenazine (1/1) (LUDFUL) Data collection

**Table:**

Enraf-Nonius CAD-4 diffractometer	*R*_int_ = 0.056
Radiation source: fine-focus sealed tube	θ_max_ = 28.0°, θ_min_ = 1.5°
Graphite monochromator	*h* = 0→19
w scans	*k* = −9→9
8396 measured reflections	*l* = −21→21
4202 independent reflections	3 standard reflections every 150 reflections
1587 reflections with *I* > 3σ(*I*)	intensity decay: 2%

### 3,5-Dinitrosalicylic acid–phenazine (1/1) (LUDFUL) Refinement

**Table:**

Refinement on *F*^2^	Secondary atom site location: difference Fourier map
Least-squares matrix: full	Hydrogen site location: difference Fourier map
*R*[*F* > 3σ(*F*)] = 0.044	H atoms treated by a mixture of independent and constrained refinement
*wR*(*F*) = 0.083	Weighting scheme based on measured s.u.'s *w* = 1/(σ^2^(*I*) + 0.0004*I*^2^)
*S* = 1.08	(Δ/σ)_max_ = 0.006
4202 reflections	Δρ_max_ = 0.29 e Å^−^^3^
274 parameters	Δρ_min_ = −0.31 e Å^−^^3^
0 restraints	Extinction correction: B-C type 1 Lorentzian isotropic (Becker & Coppens, 1974)
40 constraints	Extinction coefficient: 5100 (500)
Primary atom site location: structure-invariant direct methods	

### 3,5-Dinitrosalicylic acid–phenazine (1/1) (LUDFUL) Special details

**Table:**

Geometry. All esds (except the esd in the dihedral angle between two l.s. planes) are estimated using the full covariance matrix. The cell esds are taken into account individually in the estimation of esds in distances, angles and torsion angles; correlations between esds in cell parameters are only used when they are defined by crystal symmetry. An approximate (isotropic) treatment of cell esds is used for estimating esds involving l.s. planes.
Refinement. Refinement of F^2^ against ALL reflections. The weighted R-factor wR and goodness of fit S are based on F^2^, conventional R-factors R are based on F, with F set to zero for negative F^2^. The threshold expression of F^2^ > 2sigma(F^2^) is used only for calculating R-factors(gt) etc. and is not relevant to the choice of reflections for refinement. R-factors based on F^2^ are statistically about twice as large as those based on F, and R- factors based on ALL data will be even larger. Number of fixed parameters: 6

### 3,5-Dinitrosalicylic acid–phenazine (1/1) (LUDFUL) Fractional atomic coordinates and isotropic or equivalent isotropic displacement parameters (Å^2^)

**Table:**

	*x*	*y*	*z*	*U*_iso_*/*U*_eq_	
O3	0.98761 (10)	0.2874 (2)	0.46747 (9)	0.0599 (6)	
O1	0.85079 (8)	0.4309 (2)	0.24121 (9)	0.0539 (6)	
C1	0.98636 (12)	0.4161 (3)	0.33159 (12)	0.0373 (7)	
O7	1.12739 (10)	0.6537 (3)	0.15665 (10)	0.0716 (7)	
C4	1.17200 (13)	0.4785 (3)	0.36328 (13)	0.0421 (8)	
H4a	1.234071	0.501018	0.373432	0.0506*	
C5	1.12429 (13)	0.5256 (3)	0.28746 (12)	0.0378 (7)	
O2	0.84456 (9)	0.3110 (2)	0.36758 (9)	0.0632 (7)	
C2	1.03254 (14)	0.3661 (3)	0.40995 (13)	0.0430 (8)	
C3	1.12654 (13)	0.3980 (3)	0.42348 (12)	0.0423 (8)	
N1	1.18089 (15)	0.3455 (3)	0.50174 (12)	0.0665 (9)	
C7	0.88704 (14)	0.3818 (3)	0.31414 (14)	0.0457 (9)	
C6	1.03232 (13)	0.4947 (3)	0.27113 (12)	0.0386 (7)	
H6a	1.001506	0.526882	0.219465	0.0463*	
O6	1.25212 (10)	0.6448 (3)	0.23814 (10)	0.0804 (8)	
O4	1.14445 (14)	0.2742 (3)	0.55587 (13)	0.1291 (12)	
N2	1.17129 (12)	0.6128 (3)	0.22290 (12)	0.0504 (8)	
O5	1.26041 (12)	0.3839 (3)	0.51002 (10)	0.0996 (10)	
N3	0.68389 (10)	0.3729 (2)	0.18982 (10)	0.0388 (6)	
N4	0.50803 (11)	0.3575 (3)	0.10509 (11)	0.0517 (7)	
C17	0.57960 (14)	0.2988 (3)	0.06927 (13)	0.0505 (9)	
C19	0.61248 (13)	0.4326 (3)	0.22726 (12)	0.0377 (7)	
C8	0.74378 (15)	0.2489 (3)	0.06912 (15)	0.0552 (9)	
H8a	0.802687	0.252957	0.096215	0.0663*	
C16	0.66987 (13)	0.3064 (3)	0.11118 (13)	0.0405 (8)	
C18	0.52369 (13)	0.4267 (3)	0.18296 (13)	0.0413 (8)	
C12	0.45074 (14)	0.4976 (3)	0.22251 (15)	0.0533 (9)	
H12a	0.392387	0.498106	0.194132	0.064*	
C14	0.55304 (15)	0.5655 (3)	0.34545 (14)	0.0522 (9)	
H14a	0.561295	0.610201	0.400052	0.0627*	
C13	0.46523 (15)	0.5645 (3)	0.30108 (15)	0.0555 (10)	
H13a	0.416606	0.610633	0.326453	0.0666*	
C15	0.62592 (14)	0.5020 (3)	0.30951 (13)	0.0442 (8)	
H15a	0.683759	0.504497	0.338918	0.053*	
C10	0.6400 (2)	0.1763 (4)	−0.05197 (16)	0.0789 (12)	
H10a	0.631177	0.131533	−0.10654	0.0946*	
C11	0.56781 (17)	0.2291 (3)	−0.01395 (15)	0.0689 (11)	
H11a	0.509671	0.219906	−0.042373	0.0827*	
C9	0.72833 (18)	0.1879 (3)	−0.01060 (16)	0.0685 (12)	
H9a	0.777245	0.152877	−0.038619	0.0822*	
H3a	0.919191	0.279002	0.440153	0.138 (12)*	
H1a	0.773139	0.402666	0.229166	0.105 (8)*	

### 3,5-Dinitrosalicylic acid–phenazine (1/1) (LUDFUL) Atomic displacement parameters (Å^2^)

**Table:**

	*U*^11^	*U*^22^	*U*^33^	*U*^12^	*U*^13^	*U*^23^
O3	0.0634 (10)	0.0734 (13)	0.0442 (9)	−0.0001 (9)	0.0122 (8)	0.0094 (9)
O1	0.0315 (8)	0.0749 (13)	0.0545 (10)	−0.0027 (8)	0.0009 (7)	0.0096 (9)
C1	0.0315 (11)	0.0413 (14)	0.0394 (12)	0.0004 (10)	0.0046 (10)	−0.0033 (11)
O7	0.0556 (10)	0.1066 (17)	0.0520 (10)	−0.0111 (10)	0.0040 (8)	0.0199 (11)
C4	0.0326 (11)	0.0468 (15)	0.0457 (13)	0.0043 (11)	−0.0016 (11)	−0.0111 (12)
C5	0.0325 (11)	0.0414 (15)	0.0398 (12)	0.0028 (10)	0.0059 (10)	−0.0035 (11)
O2	0.0445 (9)	0.0836 (14)	0.0637 (10)	−0.0069 (9)	0.0161 (8)	0.0161 (10)
C2	0.0493 (13)	0.0426 (15)	0.0377 (12)	0.0029 (12)	0.0073 (11)	−0.0033 (12)
C3	0.0449 (12)	0.0468 (16)	0.0337 (12)	0.0128 (11)	−0.0024 (10)	−0.0056 (12)
N1	0.0646 (15)	0.090 (2)	0.0420 (13)	0.0141 (14)	−0.0078 (12)	−0.0014 (13)
C7	0.0413 (13)	0.0461 (16)	0.0509 (14)	−0.0004 (12)	0.0098 (11)	−0.0019 (13)
C6	0.0334 (11)	0.0433 (14)	0.0381 (12)	0.0051 (10)	−0.0003 (10)	−0.0035 (11)
O6	0.0328 (8)	0.1225 (17)	0.0871 (12)	−0.0130 (10)	0.0128 (8)	0.0119 (12)
O4	0.1041 (16)	0.197 (3)	0.0807 (15)	−0.0108 (16)	−0.0151 (13)	0.0758 (17)
N2	0.0369 (11)	0.0590 (15)	0.0567 (13)	−0.0011 (11)	0.0116 (10)	−0.0046 (12)
O5	0.0575 (11)	0.177 (2)	0.0581 (11)	0.0108 (14)	−0.0193 (9)	−0.0056 (13)
N3	0.0310 (9)	0.0431 (12)	0.0419 (10)	−0.0041 (9)	0.0024 (8)	0.0009 (10)
N4	0.0446 (11)	0.0533 (14)	0.0545 (12)	0.0017 (10)	−0.0065 (9)	−0.0035 (11)
C17	0.0538 (14)	0.0491 (17)	0.0469 (14)	0.0046 (13)	−0.0024 (12)	−0.0024 (13)
C19	0.0354 (12)	0.0362 (14)	0.0412 (13)	−0.0040 (11)	0.0029 (10)	0.0023 (11)
C8	0.0564 (15)	0.0521 (18)	0.0588 (16)	0.0030 (13)	0.0136 (13)	−0.0013 (13)
C16	0.0459 (13)	0.0339 (14)	0.0419 (13)	−0.0006 (11)	0.0057 (11)	0.0018 (11)
C18	0.0352 (11)	0.0378 (15)	0.0504 (13)	−0.0009 (11)	0.0032 (10)	−0.0008 (12)
C12	0.0355 (13)	0.0510 (17)	0.0730 (17)	0.0020 (12)	0.0041 (12)	−0.0052 (14)
C14	0.0630 (15)	0.0471 (17)	0.0484 (14)	−0.0037 (14)	0.0143 (13)	−0.0049 (13)
C13	0.0457 (14)	0.0512 (18)	0.0728 (18)	0.0032 (13)	0.0208 (13)	−0.0040 (15)
C15	0.0419 (13)	0.0481 (16)	0.0418 (13)	−0.0029 (12)	0.0012 (11)	0.0047 (12)
C10	0.107 (2)	0.078 (2)	0.0504 (16)	0.009 (2)	0.0051 (17)	−0.0198 (16)
C11	0.0775 (19)	0.071 (2)	0.0533 (17)	0.0057 (16)	−0.0147 (14)	−0.0148 (15)
C9	0.089 (2)	0.060 (2)	0.0602 (18)	0.0060 (17)	0.0270 (15)	−0.0069 (16)

### 3,5-Dinitrosalicylic acid–phenazine (1/1) (LUDFUL) Geometric parameters (Å, º)

**Table:**

O3—C2	1.329 (3)	N4—C17	1.332 (3)
O3—H3a	1.0592 (14)	N4—C18	1.344 (3)
O1—C7	1.282 (3)	C17—C16	1.428 (3)
O1—H1a	1.1628 (13)	C17—C11	1.421 (3)
C1—C2	1.410 (3)	C19—C18	1.423 (3)
C1—C7	1.487 (3)	C19—C15	1.407 (3)
C1—C6	1.373 (3)	C8—H8a	0.93
O7—N2	1.219 (2)	C8—C16	1.413 (3)
C4—H4a	0.93	C8—C9	1.349 (3)
C4—C5	1.379 (3)	C18—C12	1.412 (3)
C4—C3	1.371 (3)	C12—H12a	0.93
C5—C6	1.376 (3)	C12—C13	1.347 (3)
C5—N2	1.459 (3)	C14—H14a	0.93
O2—C7	1.234 (3)	C14—C13	1.410 (3)
C2—C3	1.404 (3)	C14—C15	1.361 (3)
C3—N1	1.464 (3)	C13—H13a	0.93
N1—O4	1.194 (3)	C15—H15a	0.93
N1—O5	1.204 (3)	C10—H10a	0.93
C6—H6a	0.93	C10—C11	1.346 (4)
O6—N2	1.217 (2)	C10—C9	1.400 (4)
N3—C19	1.346 (3)	C11—H11a	0.93
N3—C16	1.346 (3)	C9—H9a	0.93
			
C2—O3—H3a	105.60 (13)	C16—C17—C11	117.8 (2)
C7—O1—H1a	113.94 (15)	N3—C19—C18	119.68 (18)
C2—C1—C7	119.58 (18)	N3—C19—C15	120.03 (17)
C2—C1—C6	120.63 (17)	C18—C19—C15	120.28 (19)
C7—C1—C6	119.79 (17)	H8a—C8—C16	120.23
H4a—C4—C5	120.49	H8a—C8—C9	120.23
H4a—C4—C3	120.49	C16—C8—C9	119.5 (2)
C5—C4—C3	119.01 (18)	N3—C16—C17	119.52 (18)
C4—C5—C6	121.58 (19)	N3—C16—C8	120.61 (17)
C4—C5—N2	119.83 (17)	C17—C16—C8	119.86 (19)
C6—C5—N2	118.58 (17)	N4—C18—C19	121.90 (19)
C7—O2—H3a	102.24 (13)	N4—C18—C12	119.74 (18)
O3—C2—C1	120.09 (17)	C19—C18—C12	118.35 (19)
O3—C2—C3	122.05 (17)	C18—C12—H12a	119.82
C1—C2—C3	117.84 (19)	C18—C12—C13	120.35 (19)
C4—C3—C2	121.34 (18)	H12a—C12—C13	119.82
C4—C3—N1	116.79 (18)	H14a—C14—C13	119.52
C2—C3—N1	121.86 (19)	H14a—C14—C15	119.52
C3—N1—O4	119.3 (2)	C13—C14—C15	121.0 (2)
C3—N1—O5	118.0 (2)	C12—C13—C14	121.0 (2)
O4—N1—O5	122.7 (2)	C12—C13—H13a	119.52
O1—C7—C1	115.30 (19)	C14—C13—H13a	119.52
O1—C7—O2	123.93 (18)	C19—C15—C14	119.05 (18)
C1—C7—O2	120.76 (18)	C19—C15—H15a	120.48
C1—C6—C5	119.59 (17)	C14—C15—H15a	120.48
C1—C6—H6a	120.21	H10a—C10—C11	119.51
C5—C6—H6a	120.2	H10a—C10—C9	119.51
O7—N2—C5	118.45 (16)	C11—C10—C9	121.0 (2)
O7—N2—O6	122.87 (19)	C17—C11—C10	120.6 (2)
C5—N2—O6	118.66 (17)	C17—C11—H11a	119.71
C19—N3—C16	119.31 (15)	C10—C11—H11a	119.71
C19—N3—H1a	119.38 (14)	C8—C9—C10	121.3 (3)
C16—N3—H1a	120.79 (14)	C8—C9—H9a	119.37
C17—N4—C18	117.41 (17)	C10—C9—H9a	119.37
N4—C17—C16	122.14 (19)	O3—H3a—O2	151.72 (10)
N4—C17—C11	120.09 (19)	O1—H1a—N3	163.24 (10)

### 3,5-Dinitrosalicylic acid–phenazine (1/1) (LUDFUL) Hydrogen-bond geometry (Å, º)

**Table:**

*D*—H···*A*	*D*—H	H···*A*	*D*···*A*	*D*—H···*A*
C13—H13*a*···O4^i^	0.93	2.49	3.334 (3)	151
O3—H3*a*···O2	1.0592 (14)	1.5297 (14)	2.5132 (19)	151.72 (10)
O1—H1*a*···N3	1.1628 (13)	1.4160 (14)	2.5515 (19)	163.24 (10)

Symmetry code: (i) −*x*+3/2, *y*+1/2, −*z*+1.

### 2-Amino-5-methylpyridinium 2-hydroxy-3,5-dinitrobenzoate (NUQVEB) Crystal data

**Table:**

C_6_H_9_N_2_^+^·C_7_H_3_N_2_O_7_^−^	*Z* = 2
*M**_r_* = 336.27	*F*(000) = 348
Triclinic, *P*1	*D*_x_ = 1.569 Mg m^−^^3^
Hall symbol: -P 1	Mo *K*α radiation, λ = 0.71073 Å
*a* = 5.8673 (7) Å	Cell parameters from 5139 reflections
*b* = 8.0991 (9) Å	θ = 2.7–32.4°
*c* = 15.2437 (17) Å	µ = 0.13 mm^−^^1^
α = 86.844 (3)°	*T* = 100 K
β = 84.252 (3)°	Block, yellow
γ = 81.209 (3)°	0.29 × 0.14 × 0.08 mm
*V* = 711.69 (14) Å^3^	

### 2-Amino-5-methylpyridinium 2-hydroxy-3,5-dinitrobenzoate (NUQVEB) Data collection

**Table:**

Bruker APEX DUO CCD area-detector diffractometer	4943 independent reflections
Radiation source: fine-focus sealed tube	3677 reflections with *I* > 3σ(*I*)
Graphite monochromator	*R*_int_ = 0.023
φ and ω scans	θ_max_ = 32.5°, θ_min_ = 1.3°
Absorption correction: multi-scan (SADABS; Bruker, 2009)	*h* = −8→8
*T*_min_ = 0.963, *T*_max_ = 0.990	*k* = −12→11
12709 measured reflections	*l* = −22→23

### 2-Amino-5-methylpyridinium 2-hydroxy-3,5-dinitrobenzoate (NUQVEB) Refinement

**Table:**

Refinement on *F*^2^	Primary atom site location: structure-invariant direct methods
Least-squares matrix: full	Secondary atom site location: difference Fourier map
*R*[*F* > 3σ(*F*)] = 0.042	Hydrogen site location: difference Fourier map
*wR*(*F*) = 0.109	H atoms treated by a mixture of independent and constrained refinement
*S* = 2.06	Weighting scheme based on measured s.u.'s *w* = 1/(σ^2^(*I*) + 0.0004*I*^2^)
4943 reflections	(Δ/σ)_max_ = 0.009
222 parameters	Δρ_max_ = 0.40 e Å^−^^3^
0 restraints	Δρ_min_ = −0.32 e Å^−^^3^
34 constraints	

### 2-Amino-5-methylpyridinium 2-hydroxy-3,5-dinitrobenzoate (NUQVEB) Special details

**Table:**

Experimental. The crystal was placed in the cold stream of an Oxford Cryosystems Cobra open-flow nitrogen cryostat (Cosier & Glazer, 1986) operating at 100.0 (1) K.
Geometry. All s.u.'s (except the s.u. in the dihedral angle between two l.s. planes) are estimated using the full covariance matrix. The cell s.u.'s are taken into account individually in the estimation of s.u.'s in distances, angles and torsion angles; correlations between s.u.'s in cell parameters are only used when they are defined by crystal symmetry. An approximate (isotropic) treatment of cell s.u.'s is used for estimating s.u.'s involving l.s. planes.
Refinement. Refinement of F^2^ against ALL reflections. The weighted R-factor wR and goodness of fit S are based on F^2^, conventional R-factors R are based on F, with F set to zero for negative F^2^. The threshold expression of F^2^ > 2σ(F^2^) is used only for calculating R-factors(gt) etc. and is not relevant to the choice of reflections for refinement. R-factors based on F^2^ are statistically about twice as large as those based on F, and R- factors based on ALL data will be even larger. Number of fixed parameters: 15

### 2-Amino-5-methylpyridinium 2-hydroxy-3,5-dinitrobenzoate (NUQVEB) Fractional atomic coordinates and isotropic or equivalent isotropic displacement parameters (Å^2^)

**Table:**

	*x*	*y*	*z*	*U*_iso_*/*U*_eq_	Occ. (<1)
N1	0.50355 (15)	0.28624 (11)	0.24318 (6)	0.0153 (3)	
N2	0.82331 (16)	0.41649 (12)	0.19964 (6)	0.0182 (3)	
C1	0.67739 (18)	0.36376 (13)	0.26429 (7)	0.0152 (3)	
C2	0.35124 (18)	0.22197 (14)	0.30471 (7)	0.0165 (3)	
H2	0.233313	0.167725	0.285731	0.0198*	
C3	0.36539 (18)	0.23424 (14)	0.39257 (7)	0.0180 (3)	
C4	0.54266 (19)	0.31918 (15)	0.41642 (7)	0.0200 (3)	
H4	0.556083	0.332079	0.477169	0.024*	
C5	0.69461 (19)	0.38301 (14)	0.35491 (7)	0.0186 (3)	
H5	0.811136	0.439959	0.372833	0.0223*	
C6	0.2014 (2)	0.16274 (17)	0.46143 (8)	0.0262 (4)	
H6a	0.118019	0.252439	0.498084	0.0394*	
H6b	0.090144	0.110743	0.432458	0.0394*	
H6c	0.289083	0.078457	0.498608	0.0394*	
O1	0.17284 (13)	0.61818 (10)	0.14277 (5)	0.0201 (2)	
O2	0.10549 (15)	0.60964 (11)	0.31689 (5)	0.0252 (3)	
O3	0.28472 (15)	0.76461 (12)	0.38855 (5)	0.0275 (3)	
O4	0.93312 (14)	0.99538 (11)	0.26216 (6)	0.0242 (3)	
O5	0.99966 (15)	1.02486 (11)	0.11988 (6)	0.0279 (3)	
O6	0.55543 (14)	0.76370 (11)	−0.07523 (5)	0.0224 (3)	
O7	0.26607 (14)	0.63258 (10)	−0.01549 (5)	0.0199 (2)	
N3	0.25052 (15)	0.70323 (12)	0.31961 (6)	0.0172 (3)	
N4	0.89095 (16)	0.97282 (12)	0.18597 (6)	0.0190 (3)	
C7	0.33912 (17)	0.70052 (13)	0.15501 (7)	0.0141 (3)	
C8	0.38900 (17)	0.74506 (13)	0.23906 (7)	0.0144 (3)	
C9	0.56997 (17)	0.83259 (13)	0.24874 (7)	0.0156 (3)	
H9	0.601439	0.859704	0.305724	0.0187*	
C10	0.70326 (17)	0.87968 (13)	0.17493 (7)	0.0154 (3)	
C11	0.66121 (18)	0.84357 (13)	0.09021 (7)	0.0157 (3)	
H11	0.753695	0.879143	0.040052	0.0188*	
C12	0.48279 (17)	0.75522 (13)	0.08058 (7)	0.0139 (3)	
C13	0.43623 (18)	0.71649 (13)	−0.01006 (7)	0.0165 (3)	
H1o7	0.20768	0.615457	0.041923	0.044 (6)*	0.62 (3)
H1o1	0.186813	0.613081	0.081569	0.044 (6)*	0.38 (3)
H2a	0.809476	0.397973	0.143402	0.035 (4)*	
H2b	0.928572	0.469891	0.211657	0.048 (5)*	
H1	0.481117	0.276133	0.186443	0.032 (4)*	

### 2-Amino-5-methylpyridinium 2-hydroxy-3,5-dinitrobenzoate (NUQVEB) Atomic displacement parameters (Å^2^)

**Table:**

	*U*^11^	*U*^22^	*U*^33^	*U*^12^	*U*^13^	*U*^23^
N1	0.0166 (4)	0.0168 (5)	0.0130 (4)	−0.0032 (3)	−0.0023 (3)	−0.0006 (3)
N2	0.0194 (4)	0.0221 (5)	0.0147 (4)	−0.0085 (4)	−0.0004 (3)	−0.0009 (4)
C1	0.0166 (5)	0.0137 (5)	0.0152 (5)	−0.0012 (4)	−0.0022 (4)	−0.0004 (4)
C2	0.0142 (5)	0.0162 (5)	0.0192 (5)	−0.0026 (4)	−0.0015 (4)	−0.0007 (4)
C3	0.0182 (5)	0.0180 (5)	0.0168 (5)	−0.0011 (4)	0.0004 (4)	0.0009 (4)
C4	0.0239 (5)	0.0239 (6)	0.0127 (5)	−0.0043 (5)	−0.0035 (4)	−0.0007 (4)
C5	0.0201 (5)	0.0201 (6)	0.0169 (5)	−0.0054 (4)	−0.0041 (4)	−0.0004 (4)
C6	0.0238 (6)	0.0324 (7)	0.0219 (6)	−0.0077 (5)	0.0028 (4)	0.0047 (5)
O1	0.0192 (4)	0.0259 (4)	0.0176 (4)	−0.0108 (3)	−0.0027 (3)	−0.0010 (3)
O2	0.0275 (4)	0.0281 (5)	0.0220 (4)	−0.0141 (4)	0.0034 (3)	−0.0013 (3)
O3	0.0290 (5)	0.0434 (6)	0.0120 (4)	−0.0097 (4)	−0.0019 (3)	−0.0062 (4)
O4	0.0215 (4)	0.0234 (4)	0.0302 (5)	−0.0036 (3)	−0.0110 (3)	−0.0066 (4)
O5	0.0227 (4)	0.0276 (5)	0.0354 (5)	−0.0122 (4)	−0.0009 (3)	0.0022 (4)
O6	0.0280 (4)	0.0278 (5)	0.0128 (4)	−0.0102 (4)	0.0001 (3)	−0.0006 (3)
O7	0.0229 (4)	0.0252 (4)	0.0139 (4)	−0.0096 (3)	−0.0034 (3)	−0.0010 (3)
N3	0.0168 (4)	0.0200 (5)	0.0145 (4)	−0.0020 (4)	−0.0017 (3)	−0.0002 (4)
N4	0.0154 (4)	0.0150 (5)	0.0275 (5)	−0.0027 (4)	−0.0051 (4)	−0.0025 (4)
C7	0.0135 (4)	0.0136 (5)	0.0150 (5)	−0.0010 (4)	−0.0022 (4)	−0.0010 (4)
C8	0.0145 (5)	0.0155 (5)	0.0128 (5)	−0.0016 (4)	0.0000 (4)	−0.0003 (4)
C9	0.0144 (5)	0.0156 (5)	0.0169 (5)	0.0000 (4)	−0.0038 (4)	−0.0031 (4)
C10	0.0128 (4)	0.0131 (5)	0.0210 (5)	−0.0030 (4)	−0.0035 (4)	−0.0018 (4)
C11	0.0148 (5)	0.0137 (5)	0.0179 (5)	−0.0009 (4)	−0.0007 (4)	−0.0005 (4)
C12	0.0145 (4)	0.0141 (5)	0.0132 (5)	−0.0020 (4)	−0.0018 (4)	−0.0009 (4)
C13	0.0190 (5)	0.0153 (5)	0.0151 (5)	−0.0021 (4)	−0.0026 (4)	−0.0012 (4)

### 2-Amino-5-methylpyridinium 2-hydroxy-3,5-dinitrobenzoate (NUQVEB) Geometric parameters (Å, º)

**Table:**

N1—C1	1.3498 (15)	O1—H1o1	0.9310 (8)
N1—C2	1.3674 (14)	O2—N3	1.2280 (14)
N1—H1	0.8977 (9)	O3—N3	1.2338 (13)
N2—C1	1.3353 (14)	O4—N4	1.2402 (14)
N2—H2a	0.8921 (9)	O5—N4	1.2273 (13)
N2—H2b	0.8456 (10)	O6—C13	1.2340 (13)
C1—C5	1.4139 (15)	O7—C13	1.3022 (15)
C2—H2	0.95	O7—H1o7	0.9185 (8)
C2—C3	1.3602 (16)	N3—C8	1.4564 (13)
C3—C4	1.4174 (17)	N4—C10	1.4544 (15)
C3—C6	1.5049 (16)	C7—C8	1.4197 (15)
C4—H4	0.95	C7—C12	1.4357 (14)
C4—C5	1.3643 (16)	C8—C9	1.3874 (16)
C5—H5	0.95	C9—H9	0.95
C6—H6a	0.98	C9—C10	1.3750 (15)
C6—H6b	0.98	C10—C11	1.3934 (16)
C6—H6c	0.98	C11—H11	0.95
H6a—H6b	1.6003	C11—C12	1.3787 (16)
H6a—H6c	1.6003	C12—C13	1.4939 (15)
H6b—H6c	1.6003	H2a—H2b	1.4990 (2)
O1—C7	1.2964 (14)		
			
C1—N1—C2	123.29 (9)	O2—N3—O3	122.52 (9)
C1—N1—H1	120.41 (9)	O2—N3—C8	119.61 (9)
C2—N1—H1	116.29 (10)	O3—N3—C8	117.87 (10)
C1—N2—H2a	120.74 (11)	O4—N4—O5	123.30 (10)
C1—N2—H2b	120.05 (10)	O4—N4—C10	118.02 (9)
H2a—N2—H2b	119.20 (10)	O5—N4—C10	118.69 (10)
N1—C1—N2	118.98 (10)	O1—C7—C8	124.03 (9)
N1—C1—C5	117.27 (9)	O1—C7—C12	119.84 (10)
N2—C1—C5	123.75 (11)	C8—C7—C12	116.12 (10)
N1—C2—H2	119.38	N3—C8—C7	121.67 (10)
N1—C2—C3	121.25 (11)	N3—C8—C9	116.48 (9)
H2—C2—C3	119.38	C7—C8—C9	121.84 (9)
C2—C3—C4	116.54 (10)	C8—C9—H9	120.37
C2—C3—C6	122.15 (11)	C8—C9—C10	119.26 (10)
C4—C3—C6	121.31 (10)	H9—C9—C10	120.37
C3—C4—H4	118.95	N4—C10—C9	118.68 (10)
C3—C4—C5	122.10 (10)	N4—C10—C11	119.25 (9)
H4—C4—C5	118.95	C9—C10—C11	122.05 (10)
C1—C5—C4	119.51 (11)	C10—C11—H11	120.64
C1—C5—H5	120.24	C10—C11—C12	118.72 (9)
C4—C5—H5	120.24	H11—C11—C12	120.64
C3—C6—H6a	109.47	C7—C12—C11	121.98 (10)
C3—C6—H6b	109.47	C7—C12—C13	119.00 (10)
C3—C6—H6c	109.47	C11—C12—C13	119.02 (9)
H6a—C6—H6b	109.47	O6—C13—O7	123.12 (10)
H6a—C6—H6c	109.47	O6—C13—C12	120.31 (10)
H6b—C6—H6c	109.47	O7—C13—C12	116.57 (9)
C7—O1—H1o7	98.33 (6)	O1—H1o7—O7	161.55 (6)
C7—O1—H1o1	101.65 (8)	O7—H1o7—H1o1	166.67 (6)
C13—O7—H1o7	104.71 (9)	O1—H1o1—O7	163.52 (6)
C13—O7—H1o1	99.43 (7)	O1—H1o1—H1o7	171.56 (5)

### 2-Amino-5-methylpyridinium 2-hydroxy-3,5-dinitrobenzoate (NUQVEB) Hydrogen-bond geometry (Å, º)

**Table:**

*D*—H···*A*	*D*—H	H···*A*	*D*···*A*	*D*—H···*A*
C2—H2···O4^i^	0.95	2.47	3.4107 (16)	169
C4—H4···O3^ii^	0.95	2.38	3.2397 (15)	151
C5—H5···O2^iii^	0.95	2.43	3.2361 (16)	143
O7—H1*o*7···O1	0.9185 (8)	1.5313 (8)	2.4202 (12)	161.55 (6)
O1—H1*o*1···O7	0.9310 (8)	1.5130 (8)	2.4202 (12)	163.52 (6)
N2—H2*a*···O7^iv^	0.8921 (9)	2.0783 (9)	2.9655 (14)	172.84 (6)
N2—H2*b*···O1^iii^	0.8456 (10)	2.1644 (9)	2.8526 (14)	138.40 (6)
N2—H2*b*···O2^iii^	0.8456 (10)	2.4133 (10)	3.1741 (14)	150.02 (6)
N1—H1···O6^iv^	0.8977 (9)	1.7828 (9)	2.6781 (13)	174.83 (6)

Symmetry codes: (i) *x*−1, *y*−1, *z*; (ii) −*x*+1, −*y*+1, −*z*+1; (iii) *x*+1, *y*, *z*; (iv) −*x*+1, −*y*+1, −*z*.

### 3,5-Diamino-6-(2,3-dichlorophenyl)-1,2,4-triazin-2-ium 3,5-dinitro-2-hydroxybenzoate N,N-dimethylformamide monosolvate (QIQJAD) Crystal data

**Table:**

C_9_H_8_Cl_2_N_5_^+^·C_7_H_3_N_2_O_7_^−^·C_3_H_7_NO	*Z* = 2
*M**_r_* = 557.31	*F*(000) = 572
Triclinic, *P*1	*D*_x_ = 1.564 Mg m^−^^3^
Hall symbol: -P 1	Mo *K*α radiation, λ = 0.71073 Å
*a* = 10.0227 (5) Å	Cell parameters from 6413 reflections
*b* = 10.5507 (5) Å	θ = 2.3–28.2°
*c* = 12.5359 (6) Å	µ = 0.34 mm^−^^1^
α = 81.858 (1)°	*T* = 294 K
β = 71.888 (1)°	Plate, colourless
γ = 70.009 (1)°	0.16 × 0.14 × 0.08 mm
*V* = 1183.1 (1) Å^3^	

### 3,5-Diamino-6-(2,3-dichlorophenyl)-1,2,4-triazin-2-ium 3,5-dinitro-2-hydroxybenzoate N,N-dimethylformamide monosolvate (QIQJAD) Data collection

**Table:**

Bruker SMART APEX CCD area-detector diffractometer	5507 independent reflections
Radiation source: fine-focus sealed tube	4441 reflections with *I* > 3σ(*I*)
Graphite monochromator	*R*_int_ = 0.019
ω scans	θ_max_ = 28.0°, θ_min_ = 1.7°
Absorption correction: multi-scan (SADABS; Bruker, 2001)	*h* = −12→13
*T*_min_ = 0.93, *T*_max_ = 0.97	*k* = −13→13
13936 measured reflections	*l* = −16→16

### 3,5-Diamino-6-(2,3-dichlorophenyl)-1,2,4-triazin-2-ium 3,5-dinitro-2-hydroxybenzoate N,N-dimethylformamide monosolvate (QIQJAD) Refinement

**Table:**

Refinement on *F*^2^	Primary atom site location: structure-invariant direct methods
*R*[*F* > 3σ(*F*)] = 0.056	Secondary atom site location: difference Fourier map
*wR*(*F*) = 0.147	Hydrogen site location: difference Fourier map
*S* = 3.41	H atoms treated by a mixture of independent and constrained refinement
5507 reflections	Weighting scheme based on measured s.u.'s *w* = 1/(σ^2^(*I*) + 0.0004*I*^2^)
340 parameters	(Δ/σ)_max_ = 0.020
0 restraints	Δρ_max_ = 0.80 e Å^−^^3^
48 constraints	Δρ_min_ = −0.36 e Å^−^^3^

### 3,5-Diamino-6-(2,3-dichlorophenyl)-1,2,4-triazin-2-ium 3,5-dinitro-2-hydroxybenzoate N,N-dimethylformamide monosolvate (QIQJAD) Special details

**Table:**

Geometry. All e.s.d.'s (except the e.s.d. in the dihedral angle between two l.s. planes) are estimated using the full covariance matrix. The cell e.s.d.'s are taken into account individually in the estimation of e.s.d.'s in distances, angles and torsion angles; correlations between e.s.d.'s in cell parameters are only used when they are defined by crystal symmetry. An approximate (isotropic) treatment of cell e.s.d.'s is used for estimating e.s.d.'s involving l.s. planes.
Refinement. Number of fixed parameters: 18

### 3,5-Diamino-6-(2,3-dichlorophenyl)-1,2,4-triazin-2-ium 3,5-dinitro-2-hydroxybenzoate N,N-dimethylformamide monosolvate (QIQJAD) Fractional atomic coordinates and isotropic or equivalent isotropic displacement parameters (Å^2^)

**Table:**

	*x*	*y*	*z*	*U*_iso_*/*U*_eq_	
C1	0.32107 (18)	1.06764 (19)	0.15844 (16)	0.0464 (7)	
C2	0.2718 (2)	1.0232 (2)	0.08378 (15)	0.0485 (7)	
C3	0.2713 (2)	1.0903 (2)	−0.02046 (16)	0.0529 (8)	
C4	0.3231 (2)	1.2001 (2)	−0.04951 (19)	0.0621 (9)	
H4	0.323127	1.245173	−0.11886	0.0745*	
C5	0.3741 (2)	1.2420 (2)	0.02358 (19)	0.0632 (10)	
H5	0.410127	1.314857	0.001937	0.0758*	
C6	0.3745 (2)	1.1807 (2)	0.12857 (17)	0.0537 (8)	
H6	0.40816	1.211924	0.177727	0.0645*	
C7	0.32753 (18)	0.99894 (19)	0.26974 (15)	0.0437 (7)	
C8	0.35068 (19)	0.8912 (2)	0.47363 (15)	0.0449 (7)	
C9	0.19780 (18)	0.99408 (18)	0.36291 (15)	0.0418 (7)	
N1	0.45735 (16)	0.95248 (17)	0.28531 (13)	0.0488 (6)	
N2	0.46755 (16)	0.89842 (17)	0.38722 (13)	0.0484 (6)	
N3	0.37354 (18)	0.8352 (2)	0.56859 (14)	0.0613 (8)	
N4	0.21299 (15)	0.93978 (16)	0.46191 (12)	0.0454 (6)	
N5	0.06397 (16)	1.04599 (17)	0.35102 (13)	0.0504 (7)	
Cl1	0.21444 (7)	0.88366 (6)	0.11855 (5)	0.0685 (3)	
Cl2	0.20368 (7)	1.04039 (8)	−0.11161 (5)	0.0772 (3)	
C10	0.7671 (2)	0.7509 (2)	0.50260 (17)	0.0495 (8)	
C11	0.92521 (19)	0.68875 (18)	0.50532 (16)	0.0456 (8)	
C12	1.0399 (2)	0.68056 (19)	0.40501 (17)	0.0482 (8)	
C13	1.1855 (2)	0.6217 (2)	0.4120 (2)	0.0565 (9)	
C14	1.2169 (3)	0.5728 (2)	0.5120 (2)	0.0650 (11)	
H14	1.31429	0.535992	0.515021	0.078*	
C15	1.1014 (3)	0.5795 (2)	0.6070 (2)	0.0608 (10)	
C16	0.9560 (2)	0.63637 (19)	0.60559 (18)	0.0537 (9)	
H16	0.879592	0.639442	0.671313	0.0645*	
N6	1.3082 (2)	0.6096 (2)	0.3083 (2)	0.0763 (10)	
N7	1.1324 (3)	0.5213 (2)	0.7146 (3)	0.0870 (14)	
O1	0.74835 (14)	0.79734 (16)	0.40757 (12)	0.0638 (7)	
O2	0.66684 (16)	0.75284 (17)	0.58991 (13)	0.0695 (7)	
O3	1.01381 (16)	0.72881 (16)	0.30779 (13)	0.0651 (7)	
O4	1.3027 (2)	0.5624 (2)	0.22939 (18)	0.0962 (10)	
O5	1.4096 (2)	0.6460 (3)	0.3106 (2)	0.1311 (14)	
O6	1.2621 (3)	0.4654 (3)	0.7114 (2)	0.1272 (14)	
O7	1.0297 (3)	0.5356 (3)	0.7980 (2)	0.1153 (15)	
C17	0.8968 (2)	0.2419 (2)	0.13898 (19)	0.0638 (10)	
H17	0.997049	0.225753	0.10284	0.0765*	
C18	0.6486 (3)	0.3564 (4)	0.1366 (3)	0.1002 (16)	
H18a	0.600566	0.362237	0.079827	0.1503*	
H18b	0.612084	0.441665	0.171497	0.1503*	
H18c	0.628154	0.287766	0.192178	0.1503*	
C19	0.8613 (4)	0.3905 (3)	−0.0204 (2)	0.0959 (16)	
H19a	0.812684	0.38463	−0.07367	0.1439*	
H19b	0.842404	0.483699	−0.008394	0.1439*	
H19c	0.965894	0.347377	−0.048929	0.1439*	
N8	0.8043 (2)	0.32305 (17)	0.08649 (14)	0.0588 (8)	
O8	0.86513 (17)	0.18476 (18)	0.23111 (12)	0.0693 (7)	
H3n	0.459992	0.808642	0.579383	0.067 (7)*	
H4n	0.300684	0.831649	0.62665	0.058 (6)*	
H2n	0.560448	0.867423	0.392878	0.069 (7)*	
H5n	−0.01125	1.040769	0.406749	0.056 (6)*	
H6n	0.043449	1.086703	0.2906	0.052 (6)*	
H3o	0.911378	0.756483	0.330791	0.131 (12)*	

### 3,5-Diamino-6-(2,3-dichlorophenyl)-1,2,4-triazin-2-ium 3,5-dinitro-2-hydroxybenzoate N,N-dimethylformamide monosolvate (QIQJAD) Atomic displacement parameters (Å^2^)

**Table:**

	*U*^11^	*U*^22^	*U*^33^	*U*^12^	*U*^13^	*U*^23^
C1	0.0300 (8)	0.0541 (11)	0.0483 (10)	−0.0081 (8)	−0.0060 (7)	−0.0055 (8)
C2	0.0354 (9)	0.0578 (11)	0.0464 (10)	−0.0118 (8)	−0.0044 (8)	−0.0068 (8)
C3	0.0364 (9)	0.0682 (13)	0.0443 (10)	−0.0090 (9)	−0.0049 (8)	−0.0047 (9)
C4	0.0457 (11)	0.0737 (15)	0.0546 (12)	−0.0053 (10)	−0.0119 (9)	−0.0038 (11)
C5	0.0580 (13)	0.0599 (13)	0.0701 (14)	−0.0224 (11)	−0.0153 (11)	0.0054 (11)
C6	0.0442 (10)	0.0589 (12)	0.0502 (11)	−0.0107 (9)	−0.0112 (9)	0.0032 (9)
C7	0.0323 (9)	0.0527 (11)	0.0433 (10)	−0.0115 (8)	−0.0076 (7)	−0.0053 (8)
C8	0.0316 (8)	0.0564 (11)	0.0446 (10)	−0.0113 (8)	−0.0096 (7)	−0.0050 (8)
C9	0.0305 (8)	0.0502 (10)	0.0430 (9)	−0.0112 (7)	−0.0074 (7)	−0.0072 (8)
N1	0.0326 (8)	0.0622 (10)	0.0469 (9)	−0.0126 (7)	−0.0079 (6)	−0.0007 (7)
N2	0.0282 (7)	0.0679 (10)	0.0450 (9)	−0.0113 (7)	−0.0098 (6)	−0.0005 (7)
N3	0.0343 (8)	0.0949 (14)	0.0468 (9)	−0.0152 (9)	−0.0110 (7)	0.0078 (9)
N4	0.0293 (7)	0.0618 (10)	0.0417 (8)	−0.0119 (7)	−0.0077 (6)	−0.0027 (7)
N5	0.0307 (7)	0.0730 (11)	0.0421 (9)	−0.0106 (7)	−0.0102 (7)	−0.0001 (8)
Cl1	0.0784 (4)	0.0786 (4)	0.0600 (3)	−0.0419 (3)	−0.0150 (3)	−0.0059 (3)
Cl2	0.0721 (4)	0.1162 (5)	0.0522 (3)	−0.0373 (4)	−0.0190 (3)	−0.0090 (3)
C10	0.0386 (10)	0.0504 (11)	0.0597 (12)	−0.0073 (8)	−0.0180 (9)	−0.0107 (9)
C11	0.0404 (10)	0.0417 (10)	0.0597 (12)	−0.0103 (8)	−0.0219 (9)	−0.0067 (8)
C12	0.0395 (10)	0.0464 (10)	0.0641 (12)	−0.0136 (8)	−0.0218 (9)	−0.0030 (9)
C13	0.0384 (10)	0.0512 (11)	0.0826 (15)	−0.0101 (8)	−0.0220 (10)	−0.0101 (10)
C14	0.0504 (12)	0.0533 (12)	0.1065 (19)	−0.0093 (10)	−0.0465 (13)	−0.0135 (12)
C15	0.0692 (15)	0.0506 (12)	0.0797 (15)	−0.0144 (10)	−0.0492 (13)	−0.0036 (10)
C16	0.0578 (12)	0.0501 (11)	0.0625 (13)	−0.0162 (9)	−0.0287 (10)	−0.0069 (9)
N6	0.0372 (10)	0.0779 (14)	0.1062 (18)	−0.0082 (9)	−0.0156 (10)	−0.0157 (12)
N7	0.113 (2)	0.0759 (15)	0.1066 (19)	−0.0325 (14)	−0.0800 (17)	0.0073 (14)
O1	0.0374 (7)	0.0841 (11)	0.0645 (9)	−0.0093 (7)	−0.0222 (7)	0.0067 (8)
O2	0.0428 (8)	0.0946 (12)	0.0601 (9)	−0.0074 (8)	−0.0121 (7)	−0.0108 (8)
O3	0.0427 (8)	0.0802 (10)	0.0653 (9)	−0.0151 (7)	−0.0148 (7)	0.0074 (8)
O4	0.0575 (10)	0.1277 (17)	0.0882 (13)	−0.0110 (11)	−0.0135 (9)	−0.0195 (12)
O5	0.0520 (11)	0.164 (2)	0.181 (2)	−0.0480 (13)	0.0026 (13)	−0.0623 (18)
O6	0.1283 (19)	0.1273 (18)	0.142 (2)	0.0037 (15)	−0.1080 (17)	−0.0100 (15)
O7	0.142 (2)	0.159 (2)	0.0879 (15)	−0.0849 (19)	−0.0675 (16)	0.0370 (15)
C17	0.0454 (11)	0.0753 (15)	0.0575 (13)	−0.0065 (10)	−0.0078 (10)	−0.0085 (11)
C18	0.0568 (15)	0.129 (3)	0.120 (2)	−0.0180 (16)	−0.0417 (16)	−0.009 (2)
C19	0.123 (3)	0.0831 (19)	0.0697 (17)	−0.0251 (18)	−0.0261 (16)	0.0120 (14)
N8	0.0593 (11)	0.0601 (11)	0.0548 (10)	−0.0096 (9)	−0.0229 (9)	−0.0033 (8)
O8	0.0558 (9)	0.0937 (12)	0.0520 (9)	−0.0195 (8)	−0.0165 (7)	0.0097 (8)

### 3,5-Diamino-6-(2,3-dichlorophenyl)-1,2,4-triazin-2-ium 3,5-dinitro-2-hydroxybenzoate N,N-dimethylformamide monosolvate (QIQJAD) Geometric parameters (Å, º)

**Table:**

C1—C2	1.382 (3)	C13—C14	1.374 (4)
C1—C6	1.427 (3)	C13—N6	1.471 (3)
C1—C7	1.488 (3)	C14—H14	0.93
C2—C3	1.396 (3)	C14—C15	1.369 (3)
C3—C4	1.385 (4)	C15—C16	1.378 (3)
C4—H4	0.93	C15—N7	1.479 (4)
C4—C5	1.362 (4)	C16—H16	0.93
C5—H5	0.93	N6—O4	1.192 (4)
C5—C6	1.382 (3)	N6—O5	1.213 (4)
C6—H6	0.93	N7—O6	1.221 (4)
C7—C9	1.464 (2)	N7—O7	1.202 (4)
C7—N1	1.291 (2)	O3—H3o	0.9258 (14)
C8—N2	1.342 (2)	C17—H17	0.93
C8—N3	1.303 (3)	C17—N8	1.305 (3)
C8—N4	1.345 (2)	C17—O8	1.226 (3)
C9—N4	1.322 (2)	C18—H18a	0.96
C9—N5	1.312 (2)	C18—H18b	0.96
N1—N2	1.343 (2)	C18—H18c	0.96
N2—H2n	0.8973 (16)	C18—N8	1.425 (3)
N3—H3n	0.8624 (18)	H18a—H18b	1.5677
N3—H4n	0.8630 (15)	H18a—H18c	1.5677
N5—H5n	0.8658 (14)	H18b—H18c	1.5677
N5—H6n	0.8630 (16)	C19—H19a	0.96
C10—C11	1.503 (3)	C19—H19b	0.96
C10—O1	1.267 (3)	C19—H19c	0.96
C10—O2	1.231 (2)	C19—N8	1.470 (3)
C11—C12	1.405 (2)	H19a—H19b	1.5677
C11—C16	1.382 (3)	H19a—H19c	1.5677
C12—C13	1.402 (3)	H19b—H19c	1.5677
C12—O3	1.321 (3)		
			
C2—C1—C6	120.20 (18)	C11—C12—O3	122.09 (17)
C2—C1—C7	122.86 (19)	C13—C12—O3	120.57 (17)
C6—C1—C7	116.9 (2)	C12—C13—C14	122.17 (18)
C1—C2—C3	120.2 (2)	C12—C13—N6	118.7 (2)
C2—C3—C4	119.6 (2)	C14—C13—N6	119.10 (19)
C3—C4—H4	120.02	C13—C14—H14	120.79
C3—C4—C5	120.0 (2)	C13—C14—C15	118.4 (2)
H4—C4—C5	120.02	H14—C14—C15	120.79
C4—C5—H5	118.63	C14—C15—C16	122.1 (2)
C4—C5—C6	122.7 (2)	C14—C15—N7	119.4 (2)
H5—C5—C6	118.63	C16—C15—N7	118.5 (2)
C1—C6—C5	117.3 (2)	C11—C16—C15	119.19 (18)
C1—C6—H6	121.37	C11—C16—H16	120.41
C5—C6—H6	121.36	C15—C16—H16	120.41
C1—C7—C9	124.50 (16)	C13—N6—O4	118.2 (2)
C1—C7—N1	115.60 (15)	C13—N6—O5	117.0 (3)
C9—C7—N1	119.58 (16)	O4—N6—O5	124.8 (2)
N2—C8—N3	118.61 (17)	C15—N7—O6	116.6 (2)
N2—C8—N4	120.57 (17)	C15—N7—O7	118.1 (3)
N3—C8—N4	120.81 (16)	O6—N7—O7	125.3 (3)
C7—C9—N4	120.53 (16)	C10—O1—H3o	101.27 (12)
C7—C9—N5	120.93 (16)	C12—O3—H3o	99.28 (14)
N4—C9—N5	118.52 (15)	H17—C17—N8	116.69
C7—N1—N2	117.85 (14)	H17—C17—O8	116.69
C8—N2—N1	123.77 (16)	N8—C17—O8	126.6 (2)
C8—N2—H2n	122.42 (17)	H18a—C18—H18b	109.47
N1—N2—H2n	113.82 (14)	H18a—C18—H18c	109.47
C8—N3—H3n	122.28 (17)	H18a—C18—N8	109.47
C8—N3—H4n	121.07 (18)	H18b—C18—H18c	109.47
H3n—N3—H4n	116.2 (2)	H18b—C18—N8	109.47
C8—N4—C9	117.69 (14)	H18c—C18—N8	109.47
C9—N5—H5n	119.55 (17)	H19a—C19—H19b	109.47
C9—N5—H6n	124.81 (16)	H19a—C19—H19c	109.47
H5n—N5—H6n	115.64 (18)	H19a—C19—N8	109.47
C11—C10—O1	115.76 (15)	H19b—C19—H19c	109.47
C11—C10—O2	119.27 (19)	H19b—C19—N8	109.47
O1—C10—O2	124.96 (19)	H19c—C19—N8	109.47
C10—C11—C12	119.51 (18)	C17—N8—C18	120.7 (2)
C10—C11—C16	119.71 (16)	C17—N8—C19	119.5 (2)
C12—C11—C16	120.74 (18)	C18—N8—C19	119.6 (2)
C11—C12—C13	117.3 (2)	O1—H3o—O3	161.33 (12)

### 3,5-Diamino-6-(2,3-dichlorophenyl)-1,2,4-triazin-2-ium 3,5-dinitro-2-hydroxybenzoate N,N-dimethylformamide monosolvate (QIQJAD) Hydrogen-bond geometry (Å, º)

**Table:**

*D*—H···*A*	*D*—H	H···*A*	*D*···*A*	*D*—H···*A*
C19—H19*a*···O4^i^	0.96	2.47	3.401 (4)	163
N3—H3*n*···O2	0.8624 (18)	1.9939 (16)	2.854 (2)	174.78 (11)
N3—H4*n*···O8^ii^	0.8630 (15)	2.0586 (14)	2.921 (2)	176.91 (12)
N2—H2*n*···O1	0.8973 (16)	1.8310 (15)	2.728 (2)	177.45 (12)
N5—H5*n*···N4^iii^	0.8658 (14)	2.1409 (13)	2.9992 (19)	171.06 (11)
N5—H6*n*···O8^iv^	0.8630 (16)	2.0412 (16)	2.760 (2)	140.22 (10)
O3—H3*o*···O1	0.9258 (14)	1.5621 (12)	2.4572 (18)	161.33 (12)

Symmetry codes: (i) −*x*+2, −*y*+1, −*z*; (ii) −*x*+1, −*y*+1, −*z*+1; (iii) −*x*, −*y*+2, −*z*+1; (iv) *x*−1, *y*+1, *z*.

### Bis(1,10-phenanthroline-5,6-dione-κ2N,N')silver(I) 3,5-dinitrosalicylate (SAFGUD) Crystal data

**Table:**

[Ag(C_12_H_6_N_2_O_2_)](C_7_H_3_N_2_O_7_)	*F*(000) = 1512
*M**_r_* = 755.36	*D*_x_ = 1.817 Mg m^−^^3^
Monoclinic, *P*2_1_/*c*	Mo *K*α radiation, λ = 0.71073 Å
Hall symbol: -P 2ybc	Cell parameters from 5197 reflections
*a* = 11.757 (2) Å	θ = 3.2–25.4°
*b* = 18.297 (4) Å	µ = 0.81 mm^−^^1^
*c* = 13.223 (3) Å	*T* = 174 K
β = 103.91 (3)°	Prism, yellow
*V* = 2761.1 (11) Å^3^	0.3 × 0.24 × 0.2 mm
*Z* = 4	

### Bis(1,10-phenanthroline-5,6-dione-κ2N,N')silver(I) 3,5-dinitrosalicylate (SAFGUD) Data collection

**Table:**

Oxford Diffraction Gemini R Ultra diffractometer	5013 independent reflections
Radiation source: fine-focus sealed tube	3100 reflections with *I* > 3σ(*I*)
Graphite monochromator	*R*_int_ = 0.052
ω scans	θ_max_ = 25.4°, θ_min_ = 3.2°
Absorption correction: multi-scan (*SADABS*; Bruker, 2002)	*h* = −14→11
*T*_min_ = 0.780, *T*_max_ = 0.910	*k* = −17→22
12726 measured reflections	*l* = −15→13

### Bis(1,10-phenanthroline-5,6-dione-κ2N,N')silver(I) 3,5-dinitrosalicylate (SAFGUD) Refinement

**Table:**

Refinement on *F*^2^	Secondary atom site location: difference Fourier map
Least-squares matrix: full	Hydrogen site location: inferred from neighbouring sites
*R*[*F* > 3σ(*F*)] = 0.062	H-atom parameters constrained
*wR*(*F*) = 0.118	Weighting scheme based on measured s.u.'s *w* = 1/(σ^2^(*I*) + 0.0004*I*^2^)
*S* = 1.64	(Δ/σ)_max_ = 0.016
5013 reflections	Δρ_max_ = 0.76 e Å^−^^3^
444 parameters	Δρ_min_ = −0.63 e Å^−^^3^
0 restraints	Extinction correction: B-C type 1 Lorentzian isotropic (Becker & Coppens, 1974)
56 constraints	Extinction coefficient: 2400 (800)
Primary atom site location: structure-invariant direct methods	

### Bis(1,10-phenanthroline-5,6-dione-κ2N,N')silver(I) 3,5-dinitrosalicylate (SAFGUD) Special details

**Table:**

Geometry. All esds (except the esd in the dihedral angle between two l.s. planes) are estimated using the full covariance matrix. The cell esds are taken into account individually in the estimation of esds in distances, angles and torsion angles; correlations between esds in cell parameters are only used when they are defined by crystal symmetry. An approximate (isotropic) treatment of cell esds is used for estimating esds involving l.s. planes.
Refinement. Refinement of F^2^ against ALL reflections. The weighted R-factor wR and goodness of fit S are based on F^2^, conventional R-factors R are based on F, with F set to zero for negative F^2^. The threshold expression of F^2^ > 2sigma(F^2^) is used only for calculating R-factors(gt) etc. and is not relevant to the choice of reflections for refinement. R-factors based on F^2^ are statistically about twice as large as those based on F, and R- factors based on ALL data will be even larger. Number of fixed parameters 3

### Bis(1,10-phenanthroline-5,6-dione-κ2N,N')silver(I) 3,5-dinitrosalicylate (SAFGUD) Fractional atomic coordinates and isotropic or equivalent isotropic displacement parameters (Å^2^)

**Table:**

	*x*	*y*	*z*	*U*_iso_*/*U*_eq_	
Ag1	0.21070 (4)	0.44031 (3)	0.52479 (4)	0.0538 (2)	
C1	0.4796 (5)	0.5138 (3)	0.6221 (4)	0.043 (2)	
H1	0.440696	0.558209	0.60689	0.0511*	
C2	0.5975 (6)	0.5153 (3)	0.6692 (4)	0.046 (2)	
H2	0.637009	0.559483	0.684873	0.0553*	
C3	0.6551 (5)	0.4500 (3)	0.6925 (4)	0.046 (2)	
H3	0.734391	0.449118	0.725729	0.0547*	
C4	0.5946 (5)	0.3859 (3)	0.6661 (4)	0.035 (2)	
C5	0.6523 (8)	0.3141 (4)	0.6884 (5)	0.068 (3)	
C6	0.5912 (10)	0.2510 (4)	0.6718 (6)	0.093 (5)	
C7	0.4628 (6)	0.2534 (3)	0.6228 (4)	0.042 (2)	
C8	0.3994 (7)	0.1881 (3)	0.6033 (4)	0.052 (3)	
H8	0.435089	0.143184	0.6224	0.0623*	
C9	0.2837 (7)	0.1928 (4)	0.5555 (5)	0.063 (3)	
H9	0.238665	0.150616	0.540161	0.0756*	
C10	0.2345 (6)	0.2592 (3)	0.5304 (5)	0.052 (3)	
H10	0.155582	0.26107	0.496188	0.0629*	
C11	0.4065 (5)	0.3197 (3)	0.5966 (4)	0.032 (2)	
C12	0.4742 (5)	0.3885 (3)	0.6197 (4)	0.033 (2)	
C13	0.0851 (5)	0.4150 (3)	0.2774 (5)	0.054 (3)	
H13	0.109147	0.367048	0.293174	0.0649*	
C14	0.0376 (5)	0.4324 (4)	0.1743 (5)	0.060 (3)	
H14	0.029151	0.397085	0.122358	0.0717*	
C15	0.0032 (5)	0.5033 (3)	0.1508 (5)	0.051 (3)	
H15	−0.030371	0.516779	0.082274	0.0617*	
C16	0.0190 (5)	0.5550 (3)	0.2306 (4)	0.040 (2)	
C17	−0.0145 (5)	0.6317 (3)	0.2063 (5)	0.051 (3)	
C18	−0.0094 (5)	0.6826 (3)	0.2940 (5)	0.053 (3)	
C19	0.0474 (5)	0.6578 (3)	0.4023 (5)	0.042 (2)	
C20	0.0689 (5)	0.7058 (3)	0.4858 (5)	0.052 (3)	
H20	0.048099	0.754726	0.475014	0.063*	
C21	0.1204 (6)	0.6817 (4)	0.5833 (5)	0.058 (3)	
H21	0.134626	0.713123	0.64029	0.0692*	
C22	0.1503 (5)	0.6091 (4)	0.5941 (5)	0.054 (3)	
H22	0.186352	0.59254	0.660553	0.0653*	
C23	0.0818 (5)	0.5847 (3)	0.4195 (4)	0.036 (2)	
C24	0.0637 (4)	0.5318 (3)	0.3326 (4)	0.032 (2)	
C25	0.6150 (6)	0.4142 (3)	0.9276 (4)	0.041 (2)	
C26	0.5079 (6)	0.4527 (3)	0.8869 (4)	0.040 (2)	
C27	0.4074 (5)	0.4101 (3)	0.8426 (4)	0.037 (2)	
C28	0.4091 (5)	0.3351 (3)	0.8456 (4)	0.041 (2)	
H28	0.341998	0.308064	0.817616	0.0498*	
C29	0.5156 (6)	0.3004 (3)	0.8924 (4)	0.041 (2)	
C30	0.6177 (5)	0.3387 (3)	0.9323 (4)	0.043 (2)	
H30	0.687051	0.314123	0.961804	0.0515*	
C31	0.2956 (6)	0.4467 (4)	0.7881 (5)	0.047 (3)	
N1	0.4179 (4)	0.4525 (2)	0.5968 (3)	0.0338 (17)	
N2	0.2929 (4)	0.3229 (2)	0.5519 (3)	0.0387 (18)	
N3	0.0989 (4)	0.4627 (2)	0.3563 (3)	0.0413 (18)	
N4	0.1317 (4)	0.5603 (2)	0.5163 (3)	0.0405 (18)	
N5	0.7267 (6)	0.4510 (4)	0.9628 (4)	0.059 (3)	
N6	0.5168 (6)	0.2209 (3)	0.9002 (4)	0.055 (3)	
O1	0.7646 (5)	0.3117 (3)	0.7273 (4)	0.092 (3)	
O2	0.6406 (5)	0.1892 (3)	0.6987 (4)	0.102 (3)	
O3	−0.0421 (4)	0.6544 (2)	0.1169 (3)	0.069 (2)	
O4	−0.0501 (5)	0.7444 (2)	0.2776 (4)	0.081 (2)	
O5	0.8168 (5)	0.4145 (3)	0.9750 (4)	0.090 (3)	
O6	0.7298 (5)	0.5169 (3)	0.9762 (4)	0.084 (2)	
O7	0.5025 (4)	0.5233 (2)	0.8850 (3)	0.0617 (19)	
O8	0.2936 (4)	0.5168 (3)	0.7945 (3)	0.066 (2)	
O9	0.2137 (4)	0.4111 (3)	0.7370 (3)	0.064 (2)	
O10	0.4286 (5)	0.1877 (2)	0.8580 (4)	0.070 (2)	
O11	0.6096 (5)	0.1916 (2)	0.9512 (4)	0.068 (2)	
H7	0.388884	0.531747	0.83591	0.019 (12)*	

### Bis(1,10-phenanthroline-5,6-dione-κ2N,N')silver(I) 3,5-dinitrosalicylate (SAFGUD) Atomic displacement parameters (Å^2^)

**Table:**

	*U*^11^	*U*^22^	*U*^33^	*U*^12^	*U*^13^	*U*^23^
Ag1	0.0474 (4)	0.0492 (3)	0.0603 (3)	0.0150 (3)	0.0038 (2)	0.0149 (3)
C1	0.054 (5)	0.035 (4)	0.041 (3)	−0.006 (3)	0.014 (3)	0.001 (3)
C2	0.052 (5)	0.041 (4)	0.048 (4)	−0.010 (3)	0.018 (3)	−0.001 (3)
C3	0.031 (4)	0.063 (4)	0.043 (3)	−0.004 (3)	0.008 (3)	−0.008 (3)
C4	0.036 (4)	0.034 (3)	0.035 (3)	0.015 (3)	0.013 (3)	0.004 (3)
C5	0.085 (7)	0.078 (6)	0.048 (4)	0.014 (5)	0.031 (4)	0.006 (4)
C6	0.201 (12)	0.037 (5)	0.065 (5)	0.012 (6)	0.081 (7)	0.001 (4)
C7	0.033 (4)	0.059 (5)	0.035 (3)	0.007 (3)	0.008 (3)	0.001 (3)
C8	0.088 (6)	0.023 (3)	0.051 (4)	0.010 (4)	0.029 (4)	0.005 (3)
C9	0.074 (6)	0.053 (5)	0.067 (5)	−0.009 (4)	0.025 (4)	−0.008 (4)
C10	0.039 (4)	0.054 (4)	0.064 (4)	−0.003 (4)	0.012 (3)	−0.014 (4)
C11	0.043 (4)	0.026 (3)	0.029 (3)	0.006 (3)	0.010 (3)	0.002 (2)
C12	0.043 (4)	0.031 (3)	0.028 (3)	−0.004 (3)	0.014 (3)	0.002 (2)
C13	0.052 (5)	0.034 (4)	0.071 (5)	−0.002 (3)	0.005 (4)	−0.001 (3)
C14	0.055 (5)	0.055 (5)	0.064 (4)	0.000 (4)	0.003 (4)	−0.024 (4)
C15	0.046 (4)	0.057 (4)	0.045 (4)	−0.004 (3)	0.000 (3)	−0.002 (3)
C16	0.029 (3)	0.038 (3)	0.051 (4)	0.000 (3)	0.004 (3)	0.005 (3)
C17	0.039 (4)	0.054 (4)	0.055 (4)	0.002 (3)	0.001 (3)	0.021 (4)
C18	0.036 (4)	0.042 (4)	0.077 (5)	−0.003 (3)	0.004 (3)	0.005 (4)
C19	0.035 (4)	0.032 (3)	0.057 (4)	0.003 (3)	0.007 (3)	0.002 (3)
C20	0.046 (4)	0.031 (3)	0.079 (5)	0.000 (3)	0.012 (4)	−0.010 (4)
C21	0.049 (5)	0.055 (5)	0.067 (5)	0.002 (4)	0.010 (4)	−0.013 (4)
C22	0.046 (4)	0.070 (5)	0.042 (4)	−0.003 (4)	0.001 (3)	−0.006 (4)
C23	0.027 (3)	0.034 (3)	0.046 (3)	0.002 (3)	0.006 (3)	0.000 (3)
C24	0.019 (3)	0.031 (3)	0.046 (3)	−0.001 (2)	0.007 (2)	0.004 (3)
C25	0.058 (5)	0.037 (4)	0.028 (3)	−0.010 (3)	0.010 (3)	−0.004 (3)
C26	0.062 (5)	0.032 (3)	0.030 (3)	0.001 (3)	0.018 (3)	−0.002 (3)
C27	0.039 (4)	0.038 (4)	0.035 (3)	0.002 (3)	0.012 (3)	0.006 (3)
C28	0.057 (5)	0.041 (4)	0.030 (3)	−0.007 (3)	0.018 (3)	−0.002 (3)
C29	0.068 (5)	0.029 (3)	0.032 (3)	0.004 (3)	0.024 (3)	0.005 (3)
C30	0.047 (4)	0.047 (4)	0.036 (3)	0.005 (3)	0.014 (3)	0.002 (3)
C31	0.057 (5)	0.041 (4)	0.048 (4)	0.011 (4)	0.023 (3)	0.008 (3)
N1	0.036 (3)	0.031 (3)	0.033 (2)	0.005 (2)	0.008 (2)	0.001 (2)
N2	0.033 (3)	0.037 (3)	0.044 (3)	−0.001 (2)	0.006 (2)	−0.002 (2)
N3	0.040 (3)	0.029 (3)	0.051 (3)	0.001 (2)	0.004 (2)	−0.003 (2)
N4	0.035 (3)	0.043 (3)	0.041 (3)	0.003 (2)	0.004 (2)	0.006 (2)
N5	0.057 (4)	0.072 (5)	0.044 (3)	−0.015 (4)	0.003 (3)	−0.009 (3)
N6	0.090 (5)	0.039 (4)	0.044 (3)	0.004 (3)	0.033 (3)	0.004 (3)
O1	0.083 (4)	0.104 (4)	0.088 (4)	0.034 (4)	0.022 (3)	0.021 (3)
O2	0.095 (5)	0.085 (4)	0.115 (5)	0.014 (4)	0.002 (4)	−0.007 (4)
O3	0.066 (3)	0.071 (3)	0.065 (3)	0.002 (3)	0.004 (3)	0.026 (3)
O4	0.101 (4)	0.040 (3)	0.095 (4)	0.019 (3)	0.007 (3)	0.017 (3)
O5	0.052 (4)	0.100 (4)	0.111 (4)	−0.002 (3)	0.006 (3)	−0.031 (3)
O6	0.091 (4)	0.051 (3)	0.090 (4)	−0.024 (3)	−0.015 (3)	0.003 (3)
O7	0.088 (4)	0.042 (3)	0.057 (3)	−0.005 (2)	0.021 (3)	−0.002 (2)
O8	0.069 (4)	0.060 (3)	0.072 (3)	0.017 (3)	0.022 (3)	0.009 (2)
O9	0.042 (3)	0.084 (4)	0.066 (3)	0.006 (3)	0.012 (2)	−0.002 (3)
O10	0.107 (5)	0.039 (3)	0.067 (3)	−0.016 (3)	0.025 (3)	−0.001 (2)
O11	0.089 (4)	0.042 (3)	0.081 (3)	0.016 (3)	0.036 (3)	0.022 (2)

### Bis(1,10-phenanthroline-5,6-dione-κ2N,N')silver(I) 3,5-dinitrosalicylate (SAFGUD) Geometric parameters (Å, º)

**Table:**

Ag1—N1	2.404 (4)	C16—C24	1.392 (7)
Ag1—N2	2.348 (5)	C17—C18	1.476 (9)
Ag1—N3	2.335 (4)	C17—O3	1.220 (8)
Ag1—N4	2.376 (5)	C18—C19	1.498 (8)
C1—H1	0.93	C18—O4	1.227 (8)
C1—C2	1.376 (8)	C19—C20	1.386 (8)
C1—N1	1.334 (7)	C19—C23	1.400 (8)
C2—H2	0.93	C20—H20	0.93
C2—C3	1.373 (8)	C20—C21	1.359 (9)
C3—H3	0.93	C21—H21	0.93
C3—C4	1.372 (8)	C21—C22	1.373 (9)
C4—C5	1.475 (10)	C22—H22	0.93
C4—C12	1.402 (8)	C22—N4	1.341 (8)
C5—C6	1.349 (12)	C23—C24	1.478 (7)
C5—O1	1.298 (10)	C23—N4	1.349 (7)
C6—C7	1.493 (13)	C24—N3	1.344 (7)
C6—O2	1.283 (10)	C25—C26	1.430 (9)
C7—C8	1.399 (9)	C25—C30	1.384 (8)
C7—C11	1.385 (8)	C25—N5	1.450 (9)
C8—H8	0.93	C26—C27	1.418 (8)
C8—C9	1.357 (10)	C26—O7	1.293 (7)
C9—H9	0.93	C27—C28	1.373 (8)
C9—C10	1.353 (9)	C27—C31	1.496 (8)
C10—H10	0.93	C28—H28	0.93
C10—N2	1.347 (8)	C28—C29	1.407 (8)
C11—C12	1.481 (7)	C29—C30	1.380 (8)
C11—N2	1.326 (7)	C29—N6	1.458 (8)
C12—N1	1.344 (7)	C30—H30	0.93
C13—H13	0.93	C31—O8	1.286 (8)
C13—C14	1.381 (9)	C31—O9	1.223 (8)
C13—N3	1.339 (8)	N5—O5	1.230 (9)
C14—H14	0.93	N5—O6	1.218 (8)
C14—C15	1.373 (9)	N6—O10	1.216 (8)
C15—H15	0.93	N6—O11	1.257 (8)
C15—C16	1.396 (8)	O7—H7	1.346 (4)
C16—C17	1.473 (8)	O8—H7	1.155 (4)
			
H1—C1—C2	118.04	C19—C18—O4	120.9 (6)
H1—C1—N1	118.04	C18—C19—C20	121.6 (5)
C2—C1—N1	123.9 (5)	C18—C19—C23	119.3 (5)
C1—C2—H2	120.88	C20—C19—C23	119.1 (5)
C1—C2—C3	118.2 (5)	C19—C20—H20	119.84
H2—C2—C3	120.88	C19—C20—C21	120.3 (6)
C2—C3—H3	120.34	H20—C20—C21	119.84
C2—C3—C4	119.3 (5)	C20—C21—H21	121.42
H3—C3—C4	120.34	C20—C21—C22	117.2 (6)
C3—C4—C5	121.7 (6)	H21—C21—C22	121.42
C3—C4—C12	119.3 (5)	C21—C22—H22	117.46
C5—C4—C12	119.0 (5)	C21—C22—N4	125.1 (5)
C4—C5—C6	121.9 (8)	H22—C22—N4	117.46
C4—C5—O1	118.9 (7)	C19—C23—C24	121.2 (5)
C6—C5—O1	119.2 (8)	C19—C23—N4	120.8 (5)
C5—C6—C7	119.3 (7)	C24—C23—N4	118.0 (5)
C5—C6—O2	121.4 (9)	C16—C24—C23	120.2 (5)
C7—C6—O2	119.3 (7)	C16—C24—N3	122.5 (5)
C6—C7—C8	119.6 (6)	C23—C24—N3	117.2 (4)
C6—C7—C11	120.4 (6)	C26—C25—C30	121.2 (5)
C8—C7—C11	120.0 (6)	C26—C25—N5	122.7 (5)
C7—C8—H8	121.23	C30—C25—N5	116.1 (6)
C7—C8—C9	117.5 (6)	C25—C26—C27	117.1 (5)
H8—C8—C9	121.23	C25—C26—O7	122.2 (5)
C8—C9—H9	120.26	C27—C26—O7	120.5 (5)
C8—C9—C10	119.5 (6)	C26—C27—C28	122.1 (5)
H9—C9—C10	120.26	C26—C27—C31	120.1 (5)
C9—C10—H10	118.01	C28—C27—C31	117.8 (5)
C9—C10—N2	124.0 (6)	C27—C28—H28	121
H10—C10—N2	118.01	C27—C28—C29	118.0 (5)
C7—C11—C12	119.5 (5)	H28—C28—C29	121
C7—C11—N2	121.3 (5)	C28—C29—C30	122.6 (5)
C12—C11—N2	119.2 (5)	C28—C29—N6	118.3 (5)
C4—C12—C11	119.8 (5)	C30—C29—N6	119.1 (5)
C4—C12—N1	121.2 (5)	C25—C30—C29	118.7 (5)
C11—C12—N1	118.9 (5)	C25—C30—H30	120.65
H13—C13—C14	117.83	C29—C30—H30	120.65
H13—C13—N3	117.83	C27—C31—O8	116.2 (5)
C14—C13—N3	124.3 (5)	C27—C31—O9	120.8 (6)
C13—C14—H14	121.04	O8—C31—O9	122.9 (6)
C13—C14—C15	117.9 (6)	C1—N1—C12	117.9 (4)
H14—C14—C15	121.04	C10—N2—C11	117.6 (5)
C14—C15—H15	120.29	C13—N3—C24	117.3 (5)
C14—C15—C16	119.4 (5)	C22—N4—C23	117.5 (5)
H15—C15—C16	120.29	C25—N5—O5	118.4 (6)
C15—C16—C17	120.1 (5)	C25—N5—O6	120.0 (6)
C15—C16—C24	118.5 (5)	O5—N5—O6	121.6 (6)
C17—C16—C24	121.4 (5)	C29—N6—O10	118.3 (5)
C16—C17—C18	118.1 (5)	C29—N6—O11	117.2 (5)
C16—C17—O3	122.0 (6)	O10—N6—O11	124.5 (5)
C18—C17—O3	119.9 (6)	C26—O7—H7	99.4 (4)
C17—C18—C19	119.0 (5)	C31—O8—H7	103.7 (4)
C17—C18—O4	120.1 (6)	O7—H7—O8	159.6 (3)

### Bis(1,10-phenanthroline-5,6-dione-κ2N,N')silver(I) 3,5-dinitrosalicylate (SAFGUD) Hydrogen-bond geometry (Å, º)

**Table:**

*D*—H···*A*	*D*—H	H···*A*	*D*···*A*	*D*—H···*A*
C2—H2···O10^i^	0.93	2.49	3.180 (7)	131
C8—H8···O7^ii^	0.93	2.32	3.220 (7)	162
C9—H9···O6^ii^	0.93	2.49	3.243 (9)	138
C13—H13···O4^iii^	0.93	2.47	3.209 (7)	137
C22—H22···O8	0.93	2.36	3.251 (7)	160
O7—H7···C31	1.346 (4)	1.922 (7)	2.833 (8)	119.1 (3)
O7—H7···O8	1.346 (4)	1.155 (4)	2.462 (6)	159.6 (3)
O8—H7···C26	1.155 (4)	2.013 (6)	2.781 (7)	120.3 (3)
O8—H7···O7	1.155 (4)	1.346 (4)	2.462 (6)	159.6 (3)

Symmetry codes: (i) −*x*+1, *y*+1/2, −*z*+3/2; (ii) −*x*+1, *y*−1/2, −*z*+3/2; (iii) −*x*, *y*−1/2, −*z*+1/2.

### 3,5-Dimethylpyrazolium 3,5-dinitrosalicylate (SEDKET) Crystal data

**Table:**

C_5_H_9_N_2_^+^·C_7_H_3_N_2_O_7_^−^	*F*(000) = 336
*M**_r_* = 324.26	*D*_x_ = 1.547 Mg m^−^^3^
Monoclinic, *P*2_1_	Mo *K*α radiation, λ = 0.71073 Å
*a* = 8.1183 (7) Å	Cell parameters from 1025 reflections
*b* = 6.0636 (5) Å	θ = 2.5–22.6°
*c* = 14.1453 (11) Å	µ = 0.13 mm^−^^1^
β = 91.904 (1)°	*T* = 293 K
*V* = 695.93 (10) Å^3^	Block, colorless
*Z* = 2	0.40 × 0.27 × 0.11 mm

### 3,5-Dimethylpyrazolium 3,5-dinitrosalicylate (SEDKET) Data collection

**Table:**

Bruker SMART CCD diffractometer	2301 independent reflections
Radiation source: fine-focus sealed tube	1444 reflections with *I* > 3σ(*I*)
Graphite monochromator	*R*_int_ = 0.040
ω scans	θ_max_ = 25.0°, θ_min_ = 2.5°
Absorption correction: multi-scan (SADABS; Bruker, 2002)	*h* = −9→9
*T*_min_ = 0.959, *T*_max_ = 0.986	*k* = −7→7
3523 measured reflections	*l* = −16→12

### 3,5-Dimethylpyrazolium 3,5-dinitrosalicylate (SEDKET) Refinement

**Table:**

Refinement on *F*^2^	Hydrogen site location: difference Fourier map
Least-squares matrix: full	H atoms treated by a mixture of independent and constrained refinement
*R*[*F* > 3σ(*F*)] = 0.041	Weighting scheme based on measured s.u.'s *w* = 1/(σ^2^(*I*) + 0.0004*I*^2^)
*wR*(*F*) = 0.088	(Δ/σ)_max_ = 0.033
*S* = 1.16	Δρ_max_ = 0.11 e Å^−^^3^
2301 reflections	Δρ_min_ = −0.10 e Å^−^^3^
212 parameters	Extinction correction: B-C type 1 Lorentzian isotropic (Becker & Coppens, 1974)
0 restraints	Extinction coefficient: 3100 (400)
37 constraints	Absolute structure: 955 of Friedel pairs used in the refinement
Primary atom site location: structure-invariant direct methods	Absolute structure parameter: 0.5
Secondary atom site location: difference Fourier map	

### 3,5-Dimethylpyrazolium 3,5-dinitrosalicylate (SEDKET) Special details

**Table:**

Geometry. All e.s.d.'s (except the e.s.d. in the dihedral angle between two l.s. planes) are estimated using the full covariance matrix. The cell e.s.d.'s are taken into account individually in the estimation of e.s.d.'s in distances, angles and torsion angles; correlations between e.s.d.'s in cell parameters are only used when they are defined by crystal symmetry. An approximate (isotropic) treatment of cell e.s.d.'s is used for estimating e.s.d.'s involving l.s. planes.
Refinement. Refinement of *F*^2^ against ALL reflections. The weighted *R*-factor *wR* and goodness of fit *S* are based on *F*^2^, conventional *R*-factors *R* are based on *F*, with *F* set to zero for negative *F*^2^. The threshold expression of *F*^2^ > σ(*F*^2^) is used only for calculating *R*-factors(gt) *etc*. and is not relevant to the choice of reflections for refinement. *R*-factors based on *F*^2^ are statistically about twice as large as those based on *F*, and *R*- factors based on ALL data will be even larger.Number of fixed parameters 10.

### 3,5-Dimethylpyrazolium 3,5-dinitrosalicylate (SEDKET) Fractional atomic coordinates and isotropic or equivalent isotropic displacement parameters (Å^2^)

**Table:**

	*x*	*y*	*z*	*U*_iso_*/*U*_eq_	
N1	0.2262 (4)	0.7164 (5)	0.2338 (2)	0.0464 (11)	
N2	0.2203 (3)	0.6196 (5)	0.3198 (2)	0.0478 (11)	
C6	0.3519 (4)	0.7226 (6)	0.5525 (2)	0.0412 (12)	
N3	0.2179 (3)	1.3158 (5)	0.7705 (2)	0.0509 (12)	
N4	0.5552 (4)	0.7120 (5)	0.8913 (2)	0.0501 (12)	
O1	0.5417 (3)	0.5285 (4)	0.70239 (16)	0.0462 (9)	
O2	0.4266 (3)	0.5357 (4)	0.53811 (15)	0.0582 (10)	
O3	0.2657 (3)	0.8100 (4)	0.49006 (14)	0.0507 (8)	
O4	0.1415 (3)	1.4013 (4)	0.70419 (18)	0.0652 (10)	
O5	0.2270 (3)	1.3968 (4)	0.85025 (18)	0.0709 (11)	
O6	0.5263 (4)	0.7839 (5)	0.96960 (17)	0.0853 (12)	
O7	0.6549 (4)	0.5664 (5)	0.87980 (17)	0.0719 (12)	
C1	0.1300 (5)	0.6431 (8)	0.0685 (2)	0.0762 (19)	
H1a	0.132119	0.800298	0.060733	0.1144*	
H1b	0.027525	0.586015	0.043068	0.1144*	
H1c	0.219699	0.578396	0.035744	0.1144*	
C2	0.1466 (4)	0.5878 (6)	0.1713 (2)	0.0469 (13)	
C3	0.0892 (4)	0.4058 (7)	0.2187 (3)	0.0553 (14)	
H3	0.029651	0.288588	0.192446	0.0663*	
C4	0.1373 (4)	0.4313 (6)	0.3133 (2)	0.0481 (13)	
C5	0.1093 (5)	0.2896 (7)	0.3966 (3)	0.0669 (16)	
H5a	0.025142	0.183251	0.380914	0.1003*	
H5b	0.20964	0.214551	0.414477	0.1003*	
H5c	0.074882	0.379302	0.448238	0.1003*	
C12	0.4691 (4)	0.7102 (6)	0.7196 (2)	0.0372 (12)	
C7	0.3736 (3)	0.8233 (6)	0.6477 (2)	0.0348 (11)	
C8	0.2972 (4)	1.0181 (6)	0.6641 (2)	0.0380 (12)	
H8	0.239805	1.089516	0.615013	0.0456*	
C9	0.3044 (4)	1.1108 (6)	0.7534 (2)	0.0381 (11)	
C10	0.3902 (4)	1.0091 (6)	0.8274 (2)	0.0403 (12)	
H10	0.393751	1.071121	0.887603	0.0483*	
C11	0.4704 (4)	0.8133 (6)	0.8100 (2)	0.0380 (11)	
H2a	0.507 (5)	0.485 (9)	0.620 (3)	0.145 (19)*	
H1	0.289 (6)	0.878 (9)	0.225 (3)	0.130 (19)*	
H2	0.258 (4)	0.707 (6)	0.373 (2)	0.063 (12)*	

### 3,5-Dimethylpyrazolium 3,5-dinitrosalicylate (SEDKET) Atomic displacement parameters (Å^2^)

**Table:**

	*U*^11^	*U*^22^	*U*^33^	*U*^12^	*U*^13^	*U*^23^
N1	0.0502 (18)	0.049 (2)	0.0399 (18)	0.0006 (15)	−0.0027 (14)	−0.0034 (15)
N2	0.0497 (19)	0.051 (2)	0.0422 (18)	−0.0046 (17)	−0.0047 (14)	−0.0066 (17)
C6	0.0393 (19)	0.047 (2)	0.037 (2)	−0.0016 (18)	0.0053 (16)	−0.0027 (18)
N3	0.0500 (19)	0.043 (2)	0.060 (2)	−0.0003 (17)	0.0048 (16)	−0.0031 (18)
N4	0.065 (2)	0.046 (2)	0.0387 (19)	0.0015 (17)	−0.0037 (16)	0.0042 (16)
O1	0.0564 (15)	0.0414 (15)	0.0407 (14)	0.0091 (13)	−0.0027 (11)	−0.0072 (12)
O2	0.0704 (17)	0.0626 (18)	0.0414 (14)	0.0205 (15)	−0.0036 (12)	−0.0156 (14)
O3	0.0581 (14)	0.0600 (17)	0.0333 (13)	0.0050 (14)	−0.0083 (11)	0.0013 (12)
O4	0.0703 (17)	0.0521 (17)	0.0721 (18)	0.0185 (15)	−0.0141 (14)	0.0031 (15)
O5	0.092 (2)	0.0593 (19)	0.0617 (17)	0.0072 (16)	0.0048 (14)	−0.0187 (15)
O6	0.148 (3)	0.075 (2)	0.0326 (16)	0.035 (2)	−0.0069 (15)	−0.0032 (15)
O7	0.092 (2)	0.072 (2)	0.0510 (16)	0.0308 (19)	−0.0083 (14)	0.0090 (16)
C1	0.086 (3)	0.096 (4)	0.047 (2)	−0.014 (3)	−0.009 (2)	−0.007 (2)
C2	0.043 (2)	0.054 (3)	0.0434 (19)	0.005 (2)	−0.0046 (17)	−0.009 (2)
C3	0.049 (2)	0.057 (3)	0.060 (2)	−0.004 (2)	−0.0075 (18)	−0.022 (2)
C4	0.039 (2)	0.044 (2)	0.062 (2)	0.0027 (19)	0.0037 (17)	−0.003 (2)
C5	0.066 (3)	0.060 (3)	0.074 (3)	0.000 (2)	−0.002 (2)	0.012 (2)
C12	0.0351 (19)	0.041 (2)	0.036 (2)	−0.0079 (18)	0.0025 (15)	0.0010 (17)
C7	0.0342 (17)	0.039 (2)	0.0316 (17)	−0.0052 (17)	0.0004 (13)	0.0031 (16)
C8	0.041 (2)	0.038 (2)	0.0349 (19)	−0.0032 (18)	−0.0036 (15)	0.0039 (17)
C9	0.041 (2)	0.032 (2)	0.0416 (19)	−0.0045 (17)	0.0020 (15)	−0.0015 (17)
C10	0.049 (2)	0.039 (2)	0.0325 (19)	−0.0038 (18)	0.0020 (16)	−0.0017 (17)
C11	0.0419 (19)	0.041 (2)	0.0312 (17)	−0.0038 (19)	−0.0025 (14)	0.0049 (17)

### 3,5-Dimethylpyrazolium 3,5-dinitrosalicylate (SEDKET) Geometric parameters (Å, º)

**Table:**

N1—N2	1.353 (4)	H1a—H1b	1.5677
N1—C2	1.330 (5)	H1a—H1c	1.5677
N1—H1	1.11 (5)	H1b—H1c	1.5677
N2—C4	1.327 (5)	C2—C3	1.380 (5)
N2—H2	0.96 (3)	C3—H3	0.93
C6—O2	1.304 (4)	C3—C4	1.389 (5)
C6—O3	1.229 (4)	C4—C5	1.482 (5)
C6—C7	1.484 (4)	C5—H5a	0.96
N3—O4	1.223 (4)	C5—H5b	0.96
N3—O5	1.231 (4)	C5—H5c	0.96
N3—C9	1.452 (5)	H5a—H5b	1.5677
N4—O6	1.220 (4)	H5a—H5c	1.5677
N4—O7	1.213 (4)	H5b—H5c	1.5677
N4—C11	1.457 (4)	C12—C7	1.433 (4)
O1—C12	1.277 (4)	C12—C11	1.422 (4)
O1—H2a	1.22 (5)	C7—C8	1.358 (5)
O2—H2a	1.34 (5)	C8—H8	0.93
C1—H1a	0.96	C8—C9	1.382 (4)
C1—H1b	0.96	C9—C10	1.384 (4)
C1—H1c	0.96	C10—H10	0.93
C1—C2	1.494 (5)	C10—C11	1.380 (5)
			
N2—N1—C2	108.2 (3)	N2—C4—C3	106.8 (3)
N2—N1—H1	121 (2)	N2—C4—C5	122.3 (3)
C2—N1—H1	131 (2)	C3—C4—C5	130.9 (3)
N1—N2—C4	110.1 (3)	C4—C5—H5a	109.47
N1—N2—H2	117 (2)	C4—C5—H5b	109.47
C4—N2—H2	132 (2)	C4—C5—H5c	109.47
O2—C6—O3	121.3 (3)	H5a—C5—H5b	109.47
O2—C6—C7	117.2 (3)	H5a—C5—H5c	109.47
O3—C6—C7	121.5 (3)	H5b—C5—H5c	109.47
O4—N3—O5	123.2 (3)	O1—C12—C7	121.3 (3)
O4—N3—C9	118.1 (3)	O1—C12—C11	124.1 (3)
O5—N3—C9	118.7 (3)	C7—C12—C11	114.5 (3)
O6—N4—O7	122.1 (3)	C6—C7—C12	119.5 (3)
O6—N4—C11	117.8 (3)	C6—C7—C8	118.1 (3)
O7—N4—C11	120.1 (3)	C12—C7—C8	122.3 (3)
C12—O1—H2a	106 (2)	C7—C8—H8	119.87
C6—O2—H2a	106 (2)	C7—C8—C9	120.3 (3)
H1a—C1—H1b	109.47	H8—C8—C9	119.87
H1a—C1—H1c	109.47	N3—C9—C8	119.6 (3)
H1a—C1—C2	109.47	N3—C9—C10	119.3 (3)
H1b—C1—H1c	109.47	C8—C9—C10	121.0 (3)
H1b—C1—C2	109.47	C9—C10—H10	120.77
H1c—C1—C2	109.47	C9—C10—C11	118.5 (3)
N1—C2—C1	122.8 (3)	H10—C10—C11	120.77
N1—C2—C3	108.1 (3)	N4—C11—C12	120.9 (3)
C1—C2—C3	129.1 (3)	N4—C11—C10	115.7 (3)
C2—C3—H3	126.58	C12—C11—C10	123.3 (3)
C2—C3—C4	106.8 (3)	O1—H2a—O2	149 (5)
H3—C3—C4	126.58		

### 3,5-Dimethylpyrazolium 3,5-dinitrosalicylate (SEDKET) Hydrogen-bond geometry (Å, º)

**Table:**

*D*—H···*A*	*D*—H	H···*A*	*D*···*A*	*D*—H···*A*
C1—H1*a*···O7^i^	0.96	2.49	3.176 (5)	128
C5—H5*a*···O4^ii^	0.96	2.47	3.395 (5)	162
C10—H10···O6^iii^	0.93	2.47	3.369 (4)	164
O1—H2*a*···O2	1.22 (5)	1.34 (5)	2.476 (3)	149 (5)
O2—H2*a*···O1	1.34 (5)	1.22 (5)	2.476 (3)	149 (5)
N1—H1···O1^i^	1.11 (5)	1.92 (5)	2.799 (4)	133 (3)
N1—H1···O7^i^	1.11 (5)	1.94 (5)	2.850 (4)	137 (3)
N2—H2···O3	0.96 (3)	1.77 (3)	2.685 (4)	158 (3)

Symmetry codes: (i) −*x*+1, *y*+1/2, −*z*+1; (ii) −*x*, *y*−3/2, −*z*+1; (iii) −*x*+1, *y*+1/2, −*z*+2.

### 3-(1H-Imidazol-1-yl)propanaminium 2-carboxy-4,6-dinitrophenolate (TIYZIM) Crystal data

**Table:**

C_6_H_12_N_3_^+^·C_7_H_3_N_2_O_7_^−^	*Z* = 2
*M**_r_* = 353.30	*F*(000) = 368
Triclinic, *P*1	*D*_x_ = 1.525 Mg m^−^^3^
*a* = 7.0109 (4) Å	Cu *K*α radiation, λ = 1.54184 Å
*b* = 10.6617 (8) Å	Cell parameters from 2218 reflections
*c* = 10.7454 (7) Å	θ = 4.2–72.3°
α = 93.075 (6)°	µ = 1.09 mm^−^^1^
β = 95.863 (5)°	*T* = 173 K
γ = 104.944 (6)°	Irregular, yellow
*V* = 769.30 (9) Å^3^	0.22 × 0.14 × 0.12 mm

### 3-(1H-Imidazol-1-yl)propanaminium 2-carboxy-4,6-dinitrophenolate (TIYZIM) Data collection

**Table:**

Agilent Xcalibur (Eos, Gemini) diffractometer	2953 independent reflections
Graphite monochromator	2426 reflections with *I* > 3σ(*I*)
Detector resolution: 16.0416 pixels mm^-1^	*R*_int_ = 0.026
ω scans	θ_max_ = 72.5°, θ_min_ = 4.2°
Absorption correction: multi-scan (CrysAlis PRO and CrysAlis RED; Agilent, 2012)	*h* = −8→5
*T*_min_ = 0.925, *T*_max_ = 1.000	*k* = −12→13
4664 measured reflections	*l* = −13→13

### 3-(1H-Imidazol-1-yl)propanaminium 2-carboxy-4,6-dinitrophenolate (TIYZIM) Refinement

**Table:**

Refinement on *F*^2^	Primary atom site location: structure-invariant direct methods
Least-squares matrix: full	Hydrogen site location: difference Fourier map
*R*[*F* > 3σ(*F*)] = 0.041	H atoms treated by a mixture of independent and constrained refinement
*wR*(*F*) = 0.100	Weighting scheme based on measured s.u.'s *w* = 1/(σ^2^(*I*) + 0.0004*I*^2^)
*S* = 1.64	(Δ/σ)_max_ = 0.013
2953 reflections	Δρ_max_ = 0.21 e Å^−^^3^
229 parameters	Δρ_min_ = −0.18 e Å^−^^3^
0 restraints	Extinction correction: B-C type 1 Lorentzian isotropic (Becker & Coppens, 1974)
46 constraints	Extinction coefficient: 740 (130)

### 3-(1H-Imidazol-1-yl)propanaminium 2-carboxy-4,6-dinitrophenolate (TIYZIM) Special details

**Table:**

Geometry. All esds (except the esd in the dihedral angle between two l.s. planes) are estimated using the full covariance matrix. The cell esds are taken into account individually in the estimation of esds in distances, angles and torsion angles; correlations between esds in cell parameters are only used when they are defined by crystal symmetry. An approximate (isotropic) treatment of cell esds is used for estimating esds involving l.s. planes.
Refinement. Number of fixed parameters: 12

### 3-(1H-Imidazol-1-yl)propanaminium 2-carboxy-4,6-dinitrophenolate (TIYZIM) Fractional atomic coordinates and isotropic or equivalent isotropic displacement parameters (Å^2^)

**Table:**

	*x*	*y*	*z*	*U*_iso_*/*U*_eq_	
O1b	−0.19161 (17)	0.67539 (12)	0.52684 (11)	0.0294 (4)	
O2b	−0.38292 (17)	0.47166 (12)	0.40838 (12)	0.0313 (4)	
O3b	−0.25511 (17)	0.37712 (12)	0.26158 (12)	0.0312 (4)	
O4b	0.41258 (19)	0.58639 (13)	0.16853 (13)	0.0365 (5)	
O5b	0.59776 (18)	0.75622 (13)	0.28239 (14)	0.0382 (5)	
O6b	0.3446 (2)	0.93719 (14)	0.62658 (15)	0.0474 (5)	
O7b	0.02822 (19)	0.91655 (13)	0.61477 (13)	0.0410 (5)	
N1b	0.1720 (2)	0.88469 (14)	0.58118 (14)	0.0301 (5)	
N2b	0.4396 (2)	0.67347 (14)	0.25363 (14)	0.0281 (5)	
C1b	−0.0455 (2)	0.68023 (16)	0.46268 (15)	0.0225 (5)	
C2b	−0.0572 (2)	0.57873 (15)	0.36597 (15)	0.0217 (5)	
C3b	0.0987 (2)	0.57933 (16)	0.29810 (15)	0.0228 (5)	
H3b	0.086172	0.512562	0.233219	0.0273*	
C4b	0.2738 (2)	0.67709 (16)	0.32422 (15)	0.0238 (5)	
C5b	0.2967 (2)	0.77664 (16)	0.41630 (15)	0.0244 (5)	
H5b	0.418494	0.842618	0.433677	0.0292*	
C6b	0.1392 (2)	0.77850 (16)	0.48267 (15)	0.0244 (5)	
C7b	−0.2405 (2)	0.46767 (16)	0.33986 (15)	0.0245 (5)	
N1a	−0.2235 (2)	0.05127 (14)	−0.17301 (14)	0.0305 (5)	
N2a	−0.0146 (2)	0.22579 (13)	−0.06967 (13)	0.0239 (4)	
N3a	0.3467 (2)	0.20531 (13)	0.28160 (13)	0.0254 (4)	
C1a	−0.0395 (2)	0.12591 (16)	−0.15749 (16)	0.0272 (6)	
H1a	0.06324	0.111094	−0.202772	0.0327*	
C2a	−0.3211 (3)	0.10665 (17)	−0.08987 (17)	0.0314 (6)	
H2a	−0.457443	0.07432	−0.079116	0.0377*	
C3a	−0.1954 (3)	0.21356 (17)	−0.02572 (17)	0.0294 (6)	
H3a	−0.225845	0.269084	0.03706	0.0353*	
C4a	0.1719 (2)	0.32466 (17)	−0.02832 (15)	0.0268 (5)	
H4aa	0.242014	0.351332	−0.101965	0.0322*	
H4ab	0.142708	0.403798	0.008481	0.0322*	
C5a	0.3076 (2)	0.27759 (16)	0.06668 (16)	0.0271 (5)	
H5aa	0.324487	0.193134	0.033664	0.0326*	
H5ab	0.440667	0.340434	0.079114	0.0326*	
C6a	0.2253 (2)	0.26206 (16)	0.19085 (15)	0.0279 (6)	
H6aa	0.218227	0.347891	0.227027	0.0335*	
H6ab	0.08712	0.206021	0.17726	0.0335*	
H2b	−0.339134	0.554144	0.46148	0.075 (9)*	
H3aa	0.329781	0.119532	0.260701	0.045 (4)*	
H3ab	0.475827	0.248704	0.284554	0.045 (4)*	
H3ac	0.313145	0.21209	0.359189	0.045 (4)*	

### 3-(1H-Imidazol-1-yl)propanaminium 2-carboxy-4,6-dinitrophenolate (TIYZIM) Atomic displacement parameters (Å^2^)

**Table:**

	*U*^11^	*U*^22^	*U*^33^	*U*^12^	*U*^13^	*U*^23^
O1b	0.0268 (6)	0.0307 (7)	0.0277 (6)	0.0010 (5)	0.0098 (5)	−0.0050 (5)
O2b	0.0247 (6)	0.0313 (7)	0.0327 (7)	−0.0024 (5)	0.0088 (5)	−0.0060 (5)
O3b	0.0283 (6)	0.0285 (7)	0.0317 (7)	0.0002 (5)	0.0047 (5)	−0.0082 (5)
O4b	0.0371 (7)	0.0325 (7)	0.0413 (8)	0.0085 (6)	0.0167 (6)	−0.0042 (6)
O5b	0.0231 (6)	0.0380 (8)	0.0506 (9)	0.0010 (6)	0.0111 (6)	0.0014 (6)
O6b	0.0352 (7)	0.0408 (8)	0.0552 (10)	−0.0009 (6)	−0.0052 (7)	−0.0197 (7)
O7b	0.0398 (7)	0.0333 (7)	0.0470 (8)	0.0034 (6)	0.0162 (6)	−0.0121 (6)
N1b	0.0326 (8)	0.0241 (8)	0.0301 (8)	0.0012 (6)	0.0060 (6)	−0.0023 (6)
N2b	0.0260 (7)	0.0259 (7)	0.0343 (8)	0.0074 (6)	0.0092 (6)	0.0063 (6)
C1b	0.0234 (8)	0.0236 (8)	0.0201 (8)	0.0054 (6)	0.0034 (6)	0.0027 (6)
C2b	0.0218 (8)	0.0215 (8)	0.0207 (8)	0.0036 (6)	0.0019 (6)	0.0029 (6)
C3b	0.0265 (8)	0.0220 (8)	0.0208 (8)	0.0077 (7)	0.0043 (6)	0.0017 (6)
C4b	0.0220 (8)	0.0254 (8)	0.0260 (8)	0.0080 (7)	0.0064 (7)	0.0057 (7)
C5b	0.0220 (8)	0.0220 (8)	0.0269 (8)	0.0015 (6)	0.0023 (7)	0.0050 (7)
C6b	0.0276 (8)	0.0210 (8)	0.0230 (8)	0.0045 (7)	0.0018 (7)	−0.0001 (6)
C7b	0.0247 (8)	0.0261 (8)	0.0216 (8)	0.0051 (7)	0.0021 (6)	0.0007 (6)
N1a	0.0292 (8)	0.0252 (8)	0.0349 (8)	0.0050 (6)	0.0011 (6)	−0.0007 (6)
N2a	0.0247 (7)	0.0234 (7)	0.0229 (7)	0.0054 (6)	0.0034 (5)	0.0002 (6)
N3a	0.0268 (7)	0.0229 (7)	0.0245 (7)	0.0041 (6)	0.0021 (6)	−0.0026 (6)
C1a	0.0286 (9)	0.0260 (9)	0.0278 (9)	0.0087 (7)	0.0051 (7)	−0.0018 (7)
C2a	0.0269 (9)	0.0316 (10)	0.0352 (10)	0.0047 (7)	0.0073 (7)	0.0060 (8)
C3a	0.0301 (9)	0.0311 (9)	0.0289 (9)	0.0095 (7)	0.0092 (7)	0.0016 (7)
C4a	0.0277 (8)	0.0248 (8)	0.0248 (8)	0.0009 (7)	0.0052 (7)	0.0000 (7)
C5a	0.0237 (8)	0.0295 (9)	0.0260 (9)	0.0030 (7)	0.0055 (7)	−0.0021 (7)
C6a	0.0301 (9)	0.0306 (9)	0.0261 (9)	0.0125 (7)	0.0053 (7)	0.0016 (7)

### 3-(1H-Imidazol-1-yl)propanaminium 2-carboxy-4,6-dinitrophenolate (TIYZIM) Geometric parameters (Å, º)

**Table:**

O1b—C1b	1.284 (2)	N1a—C2a	1.376 (3)
O2b—C7b	1.308 (2)	N2a—C1a	1.348 (2)
O2b—H2b	0.9820 (12)	N2a—C3a	1.375 (2)
O3b—C7b	1.222 (2)	N2a—C4a	1.4622 (19)
O4b—N2b	1.232 (2)	N3a—C6a	1.483 (2)
O5b—N2b	1.2248 (17)	N3a—H3aa	0.9042 (14)
O6b—N1b	1.2313 (18)	N3a—H3ab	0.9009 (13)
O7b—N1b	1.225 (2)	N3a—H3ac	0.8932 (14)
N1b—C6b	1.464 (2)	C1a—H1a	0.95
N2b—C4b	1.458 (2)	C2a—H2a	0.95
C1b—C2b	1.440 (2)	C2a—C3a	1.351 (2)
C1b—C6b	1.428 (2)	C3a—H3a	0.95
C2b—C3b	1.373 (2)	C4a—H4aa	0.99
C2b—C7b	1.496 (2)	C4a—H4ab	0.99
C3b—H3b	0.95	C4a—C5a	1.517 (2)
C3b—C4b	1.382 (2)	C5a—H5aa	0.99
C4b—C5b	1.378 (2)	C5a—H5ab	0.99
C5b—H5b	0.95	C5a—C6a	1.507 (2)
C5b—C6b	1.378 (2)	C6a—H6aa	0.99
N1a—C1a	1.318 (2)	C6a—H6ab	0.99
			
C1b—O1b—H2b	99.82 (10)	C6a—N3a—H3ab	109.46 (13)
C7b—O2b—H2b	106.90 (11)	C6a—N3a—H3ac	111.76 (15)
O6b—N1b—O7b	123.30 (15)	H3aa—N3a—H3ab	110.21 (16)
O6b—N1b—C6b	117.72 (16)	H3aa—N3a—H3ac	106.71 (14)
O7b—N1b—C6b	118.97 (13)	H3ab—N3a—H3ac	107.27 (13)
O4b—N2b—O5b	123.59 (16)	N1a—C1a—N2a	111.91 (16)
O4b—N2b—C4b	117.89 (12)	N1a—C1a—H1a	124.04
O5b—N2b—C4b	118.52 (14)	N2a—C1a—H1a	124.04
O1b—C1b—C2b	120.19 (13)	N1a—C2a—H2a	124.86
O1b—C1b—C6b	124.76 (15)	N1a—C2a—C3a	110.28 (15)
C2b—C1b—C6b	115.00 (15)	H2a—C2a—C3a	124.86
C1b—C2b—C3b	121.54 (13)	N2a—C3a—C2a	106.10 (16)
C1b—C2b—C7b	119.88 (15)	N2a—C3a—H3a	126.95
C3b—C2b—C7b	118.56 (14)	C2a—C3a—H3a	126.95
C2b—C3b—H3b	120.01	N2a—C4a—H4aa	109.47
C2b—C3b—C4b	119.99 (15)	N2a—C4a—H4ab	109.47
H3b—C3b—C4b	120.01	N2a—C4a—C5a	112.62 (14)
N2b—C4b—C3b	119.03 (14)	H4aa—C4a—H4ab	106.12
N2b—C4b—C5b	119.24 (13)	H4aa—C4a—C5a	109.47
C3b—C4b—C5b	121.73 (16)	H4ab—C4a—C5a	109.47
C4b—C5b—H5b	120.69	C4a—C5a—H5aa	109.47
C4b—C5b—C6b	118.62 (13)	C4a—C5a—H5ab	109.47
H5b—C5b—C6b	120.69	C4a—C5a—C6a	111.50 (15)
N1b—C6b—C1b	120.26 (15)	H5aa—C5a—H5ab	107.36
N1b—C6b—C5b	116.59 (13)	H5aa—C5a—C6a	109.47
C1b—C6b—C5b	123.09 (15)	H5ab—C5a—C6a	109.47
O2b—C7b—O3b	121.86 (14)	N3a—C6a—C5a	112.35 (15)
O2b—C7b—C2b	115.78 (14)	N3a—C6a—H6aa	109.47
O3b—C7b—C2b	122.33 (16)	N3a—C6a—H6ab	109.47
C1a—N1a—C2a	105.01 (14)	C5a—C6a—H6aa	109.47
C1a—N2a—C3a	106.69 (13)	C5a—C6a—H6ab	109.47
C1a—N2a—C4a	125.73 (15)	H6aa—C6a—H6ab	106.43
C3a—N2a—C4a	127.56 (14)	O1b—H2b—O2b	156.29 (9)
C6a—N3a—H3aa	111.33 (12)		

### 3-(1H-Imidazol-1-yl)propanaminium 2-carboxy-4,6-dinitrophenolate (TIYZIM) Hydrogen-bond geometry (Å, º)

**Table:**

*D*—H···*A*	*D*—H	H···*A*	*D*···*A*	*D*—H···*A*
C4*a*—H4*aa*···O4*b*^i^	0.99	2.53	3.359 (2)	141
O2*b*—H2*b*···O1*b*	0.9820 (12)	1.5161 (11)	2.4473 (16)	156.29 (9)
O2*b*—H2*b*···C1*b*	0.9820 (12)	2.1471 (15)	2.7833 (18)	121.00 (8)
N3*a*—H3*aa*···N1*a*^ii^	0.9042 (14)	1.9318 (14)	2.797 (2)	159.6 (1)
N3*a*—H3*ab*···O2*b*^iii^	0.9009 (13)	2.5650 (12)	3.1297 (17)	121.35 (10)
N3*a*—H3*ab*···O3*b*^iii^	0.9009 (13)	2.0721 (11)	2.9537 (17)	165.79 (10)
N3*a*—H3*ac*···O1*b*^iv^	0.8932 (14)	2.0610 (13)	2.815 (2)	141.47 (11)
N3*a*—H3*ac*···O7*b*^iv^	0.8932 (14)	2.4844 (13)	2.9712 (19)	114.74 (8)

Symmetry codes: (i) −*x*+1, −*y*+1, −*z*; (ii) −*x*, −*y*, −*z*; (iii) *x*+1, *y*, *z*; (iv) −*x*, −*y*+1, −*z*+1.

### 4-{[(5-Methylisoxazol-3-yl)amino]sulfonyl}anilinium 2-hydroxy-3,5-dinitrobenzoate (TUJPEV) Crystal data

**Table:**

C_10_H_12_N_3_O_3_S^+^·C_7_H_3_N_2_O_7_^−^	*Z* = 2
*M**_r_* = 481.41	*F*(000) = 496
Triclinic, *P*1	*D*_x_ = 1.609 Mg m^−^^3^
Hall symbol: -P 1	Mo *K*α radiation, λ = 0.71073 Å
*a* = 8.5551 (1) Å	Cell parameters from 6718 reflections
*b* = 10.5000 (2) Å	θ = 1.8–32.6°
*c* = 12.7576 (3) Å	µ = 0.23 mm^−^^1^
α = 106.463 (1)°	*T* = 296 K
β = 100.913 (1)°	Prism, yellow
γ = 108.272 (1)°	0.20 × 0.20 × 0.16 mm
*V* = 993.72 (3) Å^3^	

### 4-{[(5-Methylisoxazol-3-yl)amino]sulfonyl}anilinium 2-hydroxy-3,5-dinitrobenzoate (TUJPEV) Data collection

**Table:**

Bruker Kappa APEXII CCD diffractometer	6717 independent reflections
Radiation source: fine-focus sealed tube	4398 reflections with *I* > 3σ(*I*)
Graphite monochromator	*R*_int_ = 0.030
ω and φ scan	θ_max_ = 32.6°, θ_min_ = 1.8°
Absorption correction: multi-scan (SADABS; Bruker, 2004)	*h* = −12→12
*T*_min_ = 0.955, *T*_max_ = 0.964	*k* = −15→15
24261 measured reflections	*l* = −19→16

### 4-{[(5-Methylisoxazol-3-yl)amino]sulfonyl}anilinium 2-hydroxy-3,5-dinitrobenzoate (TUJPEV) Refinement

**Table:**

Refinement on *F*^2^	Primary atom site location: structure-invariant direct methods
Least-squares matrix: full	Secondary atom site location: difference Fourier map
*R*[*F* > 3σ(*F*)] = 0.044	Hydrogen site location: difference Fourier map
*wR*(*F*) = 0.104	H atoms treated by a mixture of independent and constrained refinement
*S* = 1.95	Weighting scheme based on measured s.u.'s *w* = 1/(σ^2^(*I*) + 0.0004*I*^2^)
6717 reflections	(Δ/σ)_max_ = 0.013
301 parameters	Δρ_max_ = 0.31 e Å^−^^3^
0 restraints	Δρ_min_ = −0.35 e Å^−^^3^
48 constraints	

### 4-{[(5-Methylisoxazol-3-yl)amino]sulfonyl}anilinium 2-hydroxy-3,5-dinitrobenzoate (TUJPEV) Special details

**Table:**

Geometry. Bond distances, angles *etc*. have been calculated using the rounded fractional coordinates. All su's are estimated from the variances of the (full) variance-covariance matrix. The cell e.s.d.'s are taken into account in the estimation of distances, angles and torsion angles
Refinement. Refinement on *F*^2^ for ALL reflections except those flagged by the user for potential systematic errors. Weighted *R*-factors *wR* and all goodnesses of fit *S* are based on *F*^2^, conventional *R*-factors *R* are based on *F*, with *F* set to zero for negative *F*^2^. The observed criterion of *F*^2^ > σ(*F*^2^) is used only for calculating -*R*-factor-obs *etc*. and is not relevant to the choice of reflections for refinement. *R*-factors based on *F*^2^ are statistically about twice as large as those based on *F*, and *R*-factors based on ALL data will be even larger.Number of fixed parameters: 9

### 4-{[(5-Methylisoxazol-3-yl)amino]sulfonyl}anilinium 2-hydroxy-3,5-dinitrobenzoate (TUJPEV) Fractional atomic coordinates and isotropic or equivalent isotropic displacement parameters (Å^2^)

**Table:**

	*x*	*y*	*z*	*U*_iso_*/*U*_eq_	
S1	−0.02012 (4)	0.65156 (4)	0.38514 (3)	0.03475 (15)	
O1	−0.12136 (12)	0.56298 (10)	0.26952 (9)	0.0472 (5)	
O2	−0.09555 (13)	0.65440 (11)	0.47594 (9)	0.0468 (5)	
O3	0.47434 (14)	0.55510 (13)	0.32058 (11)	0.0626 (6)	
N1	0.29155 (15)	1.24477 (12)	0.38430 (11)	0.0434 (6)	
N2	0.13845 (14)	0.60248 (12)	0.42019 (10)	0.0376 (5)	
N3	0.39246 (16)	0.57705 (15)	0.40555 (12)	0.0536 (6)	
C1	0.07464 (15)	0.82919 (13)	0.38935 (12)	0.0322 (5)	
C2	0.21693 (19)	0.93183 (16)	0.47844 (13)	0.0511 (7)	
C3	0.28732 (19)	1.06857 (16)	0.47796 (14)	0.0514 (7)	
C4	0.21467 (16)	1.10142 (13)	0.38945 (12)	0.0344 (6)	
C5	0.07052 (18)	1.00124 (15)	0.30229 (13)	0.0444 (7)	
C6	0.00046 (17)	0.86380 (15)	0.30181 (13)	0.0421 (6)	
C7	0.24788 (17)	0.58299 (13)	0.35471 (13)	0.0355 (6)	
C8	0.2306 (2)	0.56568 (16)	0.23936 (14)	0.0473 (7)	
C9	0.3761 (2)	0.55015 (16)	0.22381 (16)	0.0511 (8)	
C10	0.4452 (3)	0.5287 (2)	0.12416 (18)	0.0766 (11)	
O4	0.51685 (14)	0.76882 (14)	0.75724 (10)	0.0657 (6)	
O5	0.25004 (13)	0.72390 (11)	0.65907 (9)	0.0495 (5)	
O6	0.03442 (11)	0.74382 (11)	0.75483 (8)	0.0431 (4)	
O7	−0.13539 (17)	0.8490 (2)	0.89544 (14)	0.0980 (10)	
O8	−0.12065 (16)	0.7660 (2)	1.03000 (12)	0.0975 (9)	
O9	0.46550 (16)	0.90412 (15)	1.25865 (10)	0.0717 (7)	
O10	0.66293 (15)	0.90569 (15)	1.17611 (11)	0.0749 (7)	
N4	−0.06158 (16)	0.80710 (17)	0.96062 (13)	0.0598 (7)	
N5	0.51466 (16)	0.88865 (13)	1.17407 (12)	0.0488 (6)	
C11	0.31789 (15)	0.78551 (13)	0.85956 (12)	0.0326 (5)	
C12	0.14714 (16)	0.77857 (13)	0.85292 (12)	0.0328 (5)	
C13	0.10840 (16)	0.80786 (15)	0.95755 (13)	0.0389 (6)	
C14	0.22578 (17)	0.84024 (15)	1.06095 (13)	0.0403 (6)	
C15	0.38931 (16)	0.84763 (14)	1.06263 (12)	0.0365 (6)	
C16	0.43735 (16)	0.82163 (13)	0.96389 (12)	0.0356 (6)	
C17	0.36856 (18)	0.75728 (15)	0.75210 (13)	0.0400 (6)	
H1a	0.188848	1.256572	0.342213	0.088 (4)*	
H1b	0.353568	1.297412	0.446823	0.088 (4)*	
H1c	0.348288	1.238627	0.338232	0.088 (4)*	
H2	0.265121	0.908789	0.538567	0.0613*	
H2a	0.186499	0.630039	0.50234	0.068 (5)*	
H3	0.383907	1.138439	0.537561	0.0617*	
H5	0.020222	1.025766	0.243815	0.0533*	
H6	−0.096798	0.794538	0.242417	0.0506*	
H8	0.139615	0.565054	0.185878	0.0568*	
H10a	0.551947	0.608597	0.142286	0.1149*	
H10b	0.362955	0.522551	0.058321	0.1149*	
H10c	0.46505	0.441064	0.1077	0.1149*	
H14	0.194965	0.856742	1.128171	0.0484*	
H16	0.549353	0.82833	0.967386	0.0428*	
H6a	0.115577	0.727699	0.69023	0.132 (9)*	

### 4-{[(5-Methylisoxazol-3-yl)amino]sulfonyl}anilinium 2-hydroxy-3,5-dinitrobenzoate (TUJPEV) Atomic displacement parameters (Å^2^)

**Table:**

	*U*^11^	*U*^22^	*U*^33^	*U*^12^	*U*^13^	*U*^23^
S1	0.03683 (17)	0.03321 (18)	0.0360 (2)	0.01370 (13)	0.01260 (14)	0.01423 (16)
O1	0.0462 (5)	0.0373 (5)	0.0422 (7)	0.0089 (4)	0.0009 (5)	0.0087 (5)
O2	0.0524 (6)	0.0510 (6)	0.0532 (7)	0.0246 (5)	0.0309 (5)	0.0276 (6)
O3	0.0518 (6)	0.0718 (8)	0.0660 (9)	0.0313 (6)	0.0248 (6)	0.0153 (7)
N1	0.0510 (7)	0.0374 (6)	0.0502 (9)	0.0178 (5)	0.0268 (6)	0.0202 (6)
N2	0.0476 (6)	0.0413 (6)	0.0327 (7)	0.0235 (5)	0.0154 (5)	0.0169 (6)
N3	0.0495 (7)	0.0628 (9)	0.0487 (9)	0.0283 (6)	0.0154 (6)	0.0136 (7)
C1	0.0350 (6)	0.0317 (7)	0.0316 (8)	0.0143 (5)	0.0112 (5)	0.0120 (6)
C2	0.0588 (9)	0.0433 (9)	0.0382 (9)	0.0107 (7)	−0.0053 (7)	0.0197 (8)
C3	0.0520 (9)	0.0380 (8)	0.0432 (10)	0.0029 (7)	−0.0033 (7)	0.0129 (8)
C4	0.0400 (7)	0.0320 (7)	0.0393 (9)	0.0172 (6)	0.0208 (6)	0.0153 (6)
C5	0.0478 (8)	0.0446 (8)	0.0438 (10)	0.0195 (7)	0.0064 (7)	0.0235 (8)
C6	0.0392 (7)	0.0397 (8)	0.0403 (9)	0.0118 (6)	0.0004 (6)	0.0159 (7)
C7	0.0414 (7)	0.0275 (7)	0.0374 (9)	0.0132 (5)	0.0131 (6)	0.0113 (6)
C8	0.0553 (9)	0.0506 (9)	0.0426 (10)	0.0238 (7)	0.0203 (7)	0.0194 (8)
C9	0.0605 (9)	0.0379 (8)	0.0574 (12)	0.0169 (7)	0.0325 (9)	0.0141 (8)
C10	0.0921 (14)	0.0731 (13)	0.0819 (15)	0.0351 (12)	0.0607 (13)	0.0275 (13)
O4	0.0483 (6)	0.0981 (10)	0.0513 (8)	0.0274 (6)	0.0254 (5)	0.0227 (7)
O5	0.0578 (6)	0.0613 (7)	0.0295 (6)	0.0241 (5)	0.0132 (5)	0.0156 (5)
O6	0.0404 (5)	0.0525 (6)	0.0322 (6)	0.0196 (4)	0.0028 (4)	0.0136 (5)
O7	0.0700 (8)	0.1711 (16)	0.0831 (11)	0.0810 (10)	0.0212 (8)	0.0529 (11)
O8	0.0537 (7)	0.1721 (16)	0.0566 (9)	0.0299 (9)	0.0287 (7)	0.0359 (10)
O9	0.0770 (8)	0.0950 (10)	0.0308 (7)	0.0267 (7)	0.0041 (6)	0.0211 (7)
O10	0.0480 (7)	0.0991 (10)	0.0639 (9)	0.0302 (6)	−0.0064 (6)	0.0241 (8)
N4	0.0396 (7)	0.0856 (11)	0.0419 (9)	0.0249 (7)	0.0082 (6)	0.0084 (8)
N5	0.0478 (7)	0.0458 (7)	0.0405 (9)	0.0147 (6)	−0.0044 (6)	0.0138 (7)
C11	0.0346 (6)	0.0281 (6)	0.0318 (8)	0.0107 (5)	0.0078 (5)	0.0096 (6)
C12	0.0351 (6)	0.0301 (7)	0.0291 (8)	0.0112 (5)	0.0039 (5)	0.0104 (6)
C13	0.0330 (6)	0.0444 (8)	0.0356 (9)	0.0154 (6)	0.0079 (6)	0.0108 (7)
C14	0.0424 (7)	0.0454 (8)	0.0301 (8)	0.0162 (6)	0.0100 (6)	0.0112 (7)
C15	0.0370 (7)	0.0341 (7)	0.0297 (8)	0.0107 (5)	−0.0009 (6)	0.0104 (6)
C16	0.0321 (6)	0.0324 (7)	0.0393 (9)	0.0124 (5)	0.0061 (6)	0.0120 (6)
C17	0.0411 (7)	0.0393 (8)	0.0385 (9)	0.0139 (6)	0.0130 (6)	0.0143 (7)

### 4-{[(5-Methylisoxazol-3-yl)amino]sulfonyl}anilinium 2-hydroxy-3,5-dinitrobenzoate (TUJPEV) Geometric parameters (Å, º)

**Table:**

S1—O1	1.4228 (9)	C9—C10	1.491 (3)
S1—O2	1.4264 (13)	C10—H10a	0.96
S1—N2	1.6264 (14)	C10—H10b	0.96
O3—N3	1.402 (2)	C10—H10c	0.96
O3—C9	1.333 (2)	O4—C17	1.223 (2)
N1—C4	1.468 (2)	O5—C17	1.2827 (18)
N1—H1a	1.0015 (14)	O5—H6a	1.2945 (12)
N1—H1b	0.7932 (11)	O6—C12	1.3010 (16)
N1—H1c	0.8315 (15)	O6—H6a	1.1837 (11)
N2—C7	1.391 (2)	O7—N4	1.211 (3)
N2—H2a	0.9703 (12)	O8—N4	1.214 (2)
N3—C7	1.312 (2)	O9—N5	1.218 (2)
C1—C2	1.3764 (15)	O10—N5	1.218 (2)
C1—C6	1.374 (2)	N4—C13	1.460 (2)
C2—C3	1.374 (2)	N5—C15	1.4638 (19)
C2—H2	0.93	C11—C12	1.425 (2)
C3—C4	1.367 (2)	C11—C16	1.3841 (19)
C3—H3	0.93	C11—C17	1.493 (2)
C4—C5	1.3668 (15)	C12—C13	1.409 (2)
C5—C6	1.376 (2)	C13—C14	1.376 (2)
C5—H5	0.93	C14—C15	1.372 (2)
C6—H6	0.93	C14—H14	0.93
C7—C8	1.403 (2)	C15—C16	1.379 (2)
C8—C9	1.348 (3)	C16—H16	0.93
C8—H8	0.93		
			
O1—S1—O2	120.50 (6)	C8—C9—C10	133.88 (19)
O1—S1—N2	108.78 (6)	C9—C10—H10a	109.47
O2—S1—N2	104.15 (7)	C9—C10—H10b	109.47
N3—O3—C9	108.94 (14)	C9—C10—H10c	109.47
C4—N1—H1a	102.59 (10)	H10a—C10—H10b	109.47
C4—N1—H1b	107.06 (15)	H10a—C10—H10c	109.47
C4—N1—H1c	107.83 (13)	H10b—C10—H10c	109.47
H1a—N1—H1b	124.65 (16)	C17—O5—H6a	105.28 (11)
H1a—N1—H1c	103.41 (14)	C12—O6—H6a	102.01 (10)
H1b—N1—H1c	110.21 (14)	O7—N4—O8	123.34 (17)
S1—N2—C7	124.43 (12)	O7—N4—C13	118.74 (17)
S1—N2—H2a	113.65 (11)	O8—N4—C13	117.91 (17)
C7—N2—H2a	116.39 (12)	O9—N5—O10	124.03 (14)
O3—N3—C7	104.86 (14)	O9—N5—C15	118.56 (14)
C2—C1—C6	120.38 (14)	O10—N5—C15	117.41 (15)
C1—C2—C3	119.57 (16)	C12—C11—C16	120.99 (14)
C1—C2—H2	120.22	C12—C11—C17	118.93 (12)
C3—C2—H2	120.22	C16—C11—C17	120.07 (13)
C2—C3—C4	119.68 (12)	O6—C12—C11	121.05 (14)
C2—C3—H3	120.16	O6—C12—C13	122.90 (13)
C4—C3—H3	120.16	C11—C12—C13	116.03 (12)
N1—C4—C3	120.80 (10)	N4—C13—C12	120.40 (13)
N1—C4—C5	118.09 (15)	N4—C13—C14	116.57 (15)
C3—C4—C5	121.09 (14)	C12—C13—C14	123.02 (14)
C4—C5—C6	119.44 (16)	C13—C14—C15	118.50 (15)
C4—C5—H5	120.28	C13—C14—H14	120.75
C6—C5—H5	120.28	C15—C14—H14	120.75
C1—C6—C5	119.80 (11)	N5—C15—C14	117.72 (14)
C1—C6—H6	120.1	N5—C15—C16	120.43 (13)
C5—C6—H6	120.1	C14—C15—C16	121.82 (13)
N2—C7—N3	117.05 (14)	C11—C16—C15	119.61 (13)
N2—C7—C8	131.01 (14)	C11—C16—H16	120.19
N3—C7—C8	111.93 (15)	C15—C16—H16	120.19
C7—C8—C9	104.20 (16)	O4—C17—O5	124.29 (16)
C7—C8—H8	127.9	O4—C17—C11	119.59 (13)
C9—C8—H8	127.9	O5—C17—C11	116.11 (13)
O3—C9—C8	110.05 (18)	O5—H6a—O6	156.58 (6)
O3—C9—C10	116.07 (17)		

### 4-{[(5-Methylisoxazol-3-yl)amino]sulfonyl}anilinium 2-hydroxy-3,5-dinitrobenzoate (TUJPEV) Hydrogen-bond geometry (Å, º)

**Table:**

*D*—H···*A*	*D*—H	H···*A*	*D*···*A*	*D*—H···*A*
N1—H1*a*···O6^i^	1.0015 (14)	2.0683 (10)	3.0655 (17)	173.55 (7)
N1—H1*b*···N3^ii^	0.7932 (11)	2.2920 (11)	3.0393 (15)	157.33 (11)
N1—H1*c*···O4^ii^	0.8315 (15)	1.8318 (14)	2.663 (2)	177.12 (7)
N2—H2*a*···O5	0.9703 (12)	1.8440 (10)	2.7852 (15)	162.64 (9)
O5—H6*a*···O6	1.2945 (12)	1.1837 (11)	2.4268 (16)	156.58 (6)
O5—H6*a*···C12	1.2945 (12)	1.9326 (15)	2.7490 (19)	115.40 (6)
O6—H6*a*···O5	1.1837 (11)	1.2945 (12)	2.4268 (16)	156.58 (6)
O6—H6*a*···C17	1.1837 (11)	2.0485 (15)	2.8249 (19)	119.42 (7)

Symmetry codes: (i) −*x*, −*y*+2, −*z*+1; (ii) −*x*+1, −*y*+2, −*z*+1.

### 2-Isopropyl-6-methyl-4-oxo-3,4-dihydropyrimidin-1-ium 2-carboxy-4,6-dinitrophenolate monohydrate (VABZIJ) Crystal data

**Table:**

C_8_H_13_N_2_O^+^·C_7_H_3_N_2_O_7_^−^·H_2_O	*Z* = 2
*M**_r_* = 398.33	*F*(000) = 416
Triclinic, *P*1	*D*_x_ = 1.497 Mg m^−^^3^
Hall symbol: -P 1	Mo *K*α radiation, λ = 0.71073 Å
*a* = 6.6691 (3) Å	Cell parameters from 6994 reflections
*b* = 11.3831 (4) Å	θ = 2.4–31.6°
*c* = 12.2900 (5) Å	µ = 0.13 mm^−^^1^
α = 89.727 (2)°	*T* = 100 K
β = 76.771 (2)°	Block, yellow
γ = 76.930 (2)°	0.52 × 0.13 × 0.10 mm
*V* = 883.62 (6) Å^3^	

### 2-Isopropyl-6-methyl-4-oxo-3,4-dihydropyrimidin-1-ium 2-carboxy-4,6-dinitrophenolate monohydrate (VABZIJ) Data collection

**Table:**

Bruker SMART APEXII CCD area-detector diffractometer	4061 independent reflections
Radiation source: fine-focus sealed tube	3042 reflections with *I* > 3σ(*I*)
Graphite monochromator	*R*_int_ = 0.030
φ and ω scans	θ_max_ = 27.5°, θ_min_ = 1.7°
Absorption correction: multi-scan (SADABS; Bruker, 2009)	*h* = −8→8
*T*_min_ = 0.937, *T*_max_ = 0.987	*k* = −14→12
17014 measured reflections	*l* = −15→15

### 2-Isopropyl-6-methyl-4-oxo-3,4-dihydropyrimidin-1-ium 2-carboxy-4,6-dinitrophenolate monohydrate (VABZIJ) Refinement

**Table:**

Refinement on *F*^2^	Primary atom site location: structure-invariant direct methods
*R*[*F* > 3σ(*F*)] = 0.038	Secondary atom site location: difference Fourier map
*wR*(*F*) = 0.086	Hydrogen site location: difference Fourier map
*S* = 1.77	H atoms treated by a mixture of independent and constrained refinement
4061 reflections	Weighting scheme based on measured s.u.'s *w* = 1/(σ^2^(*I*) + 0.0004*I*^2^)
258 parameters	(Δ/σ)_max_ = 0.014
0 restraints	Δρ_max_ = 0.46 e Å^−^^3^
52 constraints	Δρ_min_ = −0.23 e Å^−^^3^

### 2-Isopropyl-6-methyl-4-oxo-3,4-dihydropyrimidin-1-ium 2-carboxy-4,6-dinitrophenolate monohydrate (VABZIJ) Special details

**Table:**

Experimental. The crystal was placed in the cold stream of an Oxford Cryosystems Cobra open-flow nitrogen cryostat (Cosier & Glazer, 1986) operating at 100.0 (1) K.
Geometry. All s.u.'s (except the s.u. in the dihedral angle between two l.s. planes) are estimated using the full covariance matrix. The cell s.u.'s are taken into account individually in the estimation of s.u.'s in distances, angles and torsion angles; correlations between s.u.'s in cell parameters are only used when they are defined by crystal symmetry. An approximate (isotropic) treatment of cell s.u.'s is used for estimating s.u.'s involving l.s. planes.
Refinement. Refinement of F^2^ against ALL reflections. The weighted R-factor wR and goodness of fit S are based on F^2^, conventional R-factors R are based on F, with F set to zero for negative F^2^. The threshold expression of F^2^ > 2σ(F^2^) is used only for calculating R-factors(gt) etc. and is not relevant to the choice of reflections for refinement. R-factors based on F^2^ are statistically about twice as large as those based on F, and R- factors based on ALL data will be even larger. Number of fixed parameters: 15

### 2-Isopropyl-6-methyl-4-oxo-3,4-dihydropyrimidin-1-ium 2-carboxy-4,6-dinitrophenolate monohydrate (VABZIJ) Fractional atomic coordinates and isotropic or equivalent isotropic displacement parameters (Å^2^)

**Table:**

	*x*	*y*	*z*	*U*_iso_*/*U*_eq_	
O1	0.30546 (16)	−0.17099 (9)	0.14963 (8)	0.0232 (4)	
O2	0.56653 (17)	−0.35718 (9)	0.01361 (9)	0.0316 (4)	
O3	0.48656 (18)	−0.36691 (9)	−0.14697 (9)	0.0311 (4)	
O4	0.36254 (16)	−0.00313 (9)	−0.33889 (8)	0.0235 (4)	
O5	0.23753 (16)	0.16995 (9)	−0.24573 (8)	0.0255 (4)	
O6	0.07250 (15)	0.19153 (8)	0.16330 (8)	0.0204 (4)	
O7	0.14533 (16)	0.02234 (9)	0.25238 (8)	0.0215 (4)	
O8	0.31889 (16)	0.38510 (9)	0.41956 (8)	0.0221 (4)	
N1	0.30174 (18)	0.05930 (11)	−0.25094 (10)	0.0185 (4)	
N2	0.48606 (19)	−0.31064 (11)	−0.06125 (10)	0.0213 (4)	
N3	0.11898 (17)	0.64252 (10)	0.66449 (9)	0.0147 (4)	
N4	0.23841 (17)	0.58259 (10)	0.47936 (9)	0.0144 (4)	
C1	0.3103 (2)	−0.11910 (12)	0.05547 (11)	0.0151 (5)	
C2	0.3895 (2)	−0.18160 (12)	−0.05145 (11)	0.0154 (5)	
C3	0.3838 (2)	−0.12419 (13)	−0.15034 (12)	0.0160 (5)	
H3a	0.432743	−0.167918	−0.218686	0.0192*	
C4	0.3044 (2)	−0.00126 (13)	−0.14615 (11)	0.0150 (5)	
C5	0.2281 (2)	0.06626 (13)	−0.04539 (11)	0.0148 (5)	
H5a	0.176964	0.149404	−0.044656	0.0177*	
C6	0.2290 (2)	0.00871 (12)	0.05379 (11)	0.0140 (5)	
C7	0.1429 (2)	0.08121 (13)	0.16123 (11)	0.0159 (5)	
C8	0.2598 (2)	0.45829 (12)	0.49910 (12)	0.0158 (5)	
C9	0.2070 (2)	0.43367 (13)	0.61582 (11)	0.0160 (5)	
H9a	0.221191	0.354014	0.636387	0.0192*	
C10	0.1376 (2)	0.52320 (12)	0.69587 (11)	0.0157 (5)	
C11	0.1694 (2)	0.67095 (12)	0.55875 (11)	0.0141 (5)	
C12	0.1445 (2)	0.80006 (12)	0.52928 (11)	0.0153 (5)	
H12a	0.110348	0.85057	0.598371	0.0184*	
C13	−0.0374 (2)	0.83589 (13)	0.47019 (13)	0.0224 (5)	
H13a	−0.054927	0.919318	0.452627	0.0335*	
H13b	−0.165759	0.824046	0.518462	0.0335*	
H13c	−0.005734	0.786763	0.402409	0.0335*	
C14	0.3509 (2)	0.82298 (12)	0.45745 (12)	0.0195 (5)	
H14a	0.461527	0.800548	0.496897	0.0292*	
H14b	0.331515	0.907081	0.442205	0.0292*	
H14c	0.389104	0.775688	0.38823	0.0292*	
C15	0.0772 (2)	0.50600 (13)	0.81821 (12)	0.0221 (5)	
H15a	−0.068894	0.545223	0.847126	0.0332*	
H15b	0.163895	0.540389	0.855439	0.0332*	
H15c	0.097793	0.421295	0.830953	0.0332*	
O1w	0.32852 (17)	0.60955 (9)	0.25340 (8)	0.0272 (4)	
H1n3	0.061415	0.706878	0.722609	0.037 (5)*	
H1n4	0.270467	0.602234	0.406704	0.034 (5)*	
H2w1	0.339729	0.675124	0.210779	0.053 (6)*	
H1w1	0.381611	0.539911	0.20932	0.055 (6)*	
H7	0.211364	−0.063754	0.220331	0.078 (7)*	

### 2-Isopropyl-6-methyl-4-oxo-3,4-dihydropyrimidin-1-ium 2-carboxy-4,6-dinitrophenolate monohydrate (VABZIJ) Atomic displacement parameters (Å^2^)

**Table:**

	*U*^11^	*U*^22^	*U*^33^	*U*^12^	*U*^13^	*U*^23^
O1	0.0305 (6)	0.0178 (6)	0.0185 (6)	−0.0038 (5)	−0.0018 (5)	0.0031 (4)
O2	0.0431 (7)	0.0219 (6)	0.0232 (6)	0.0045 (5)	−0.0063 (5)	0.0044 (5)
O3	0.0489 (7)	0.0181 (6)	0.0229 (6)	−0.0036 (5)	−0.0053 (5)	−0.0086 (5)
O4	0.0291 (6)	0.0274 (6)	0.0138 (6)	−0.0083 (5)	−0.0030 (5)	−0.0009 (5)
O5	0.0347 (6)	0.0178 (6)	0.0235 (6)	−0.0045 (5)	−0.0072 (5)	0.0058 (5)
O6	0.0244 (6)	0.0142 (6)	0.0187 (6)	−0.0013 (4)	−0.0004 (4)	−0.0029 (4)
O7	0.0284 (6)	0.0175 (6)	0.0140 (5)	−0.0009 (5)	0.0003 (4)	−0.0003 (4)
O8	0.0321 (6)	0.0137 (5)	0.0176 (6)	−0.0026 (5)	−0.0027 (5)	−0.0025 (4)
N1	0.0182 (6)	0.0202 (7)	0.0186 (7)	−0.0071 (6)	−0.0047 (5)	0.0034 (6)
N2	0.0240 (7)	0.0161 (7)	0.0198 (7)	−0.0035 (6)	0.0019 (6)	0.0001 (6)
N3	0.0168 (6)	0.0123 (6)	0.0136 (6)	−0.0024 (5)	−0.0017 (5)	−0.0006 (5)
N4	0.0172 (6)	0.0117 (6)	0.0129 (6)	−0.0026 (5)	−0.0014 (5)	0.0011 (5)
C1	0.0133 (7)	0.0177 (8)	0.0148 (7)	−0.0064 (6)	−0.0014 (6)	0.0022 (6)
C2	0.0158 (7)	0.0118 (7)	0.0176 (8)	−0.0030 (6)	−0.0017 (6)	−0.0008 (6)
C3	0.0159 (7)	0.0184 (8)	0.0140 (8)	−0.0070 (6)	−0.0012 (6)	−0.0029 (6)
C4	0.0146 (7)	0.0196 (8)	0.0125 (7)	−0.0073 (6)	−0.0030 (6)	0.0033 (6)
C5	0.0121 (7)	0.0137 (7)	0.0188 (8)	−0.0046 (6)	−0.0026 (6)	0.0007 (6)
C6	0.0113 (7)	0.0154 (7)	0.0154 (7)	−0.0048 (6)	−0.0015 (6)	−0.0005 (6)
C7	0.0130 (7)	0.0171 (8)	0.0170 (8)	−0.0048 (6)	−0.0008 (6)	0.0000 (6)
C8	0.0145 (7)	0.0132 (7)	0.0193 (8)	−0.0022 (6)	−0.0036 (6)	0.0001 (6)
C9	0.0177 (7)	0.0110 (7)	0.0187 (8)	−0.0031 (6)	−0.0036 (6)	0.0032 (6)
C10	0.0137 (7)	0.0154 (8)	0.0182 (8)	−0.0033 (6)	−0.0041 (6)	0.0029 (6)
C11	0.0106 (7)	0.0152 (7)	0.0161 (8)	−0.0028 (6)	−0.0026 (6)	−0.0012 (6)
C12	0.0178 (7)	0.0114 (7)	0.0155 (7)	−0.0034 (6)	−0.0014 (6)	−0.0007 (6)
C13	0.0218 (8)	0.0140 (8)	0.0321 (9)	−0.0033 (6)	−0.0089 (7)	0.0052 (7)
C14	0.0210 (8)	0.0141 (8)	0.0218 (8)	−0.0048 (6)	−0.0012 (6)	0.0004 (6)
C15	0.0269 (8)	0.0194 (8)	0.0188 (8)	−0.0047 (7)	−0.0034 (6)	0.0019 (6)
O1w	0.0471 (7)	0.0134 (6)	0.0163 (6)	−0.0043 (5)	−0.0005 (5)	0.0003 (5)

### 2-Isopropyl-6-methyl-4-oxo-3,4-dihydropyrimidin-1-ium 2-carboxy-4,6-dinitrophenolate monohydrate (VABZIJ) Geometric parameters (Å, º)

**Table:**

O1—C1	1.2939 (17)	C4—C5	1.3881 (18)
O1—H7	1.4329 (9)	C5—H5a	0.93
O2—N2	1.2279 (17)	C5—C6	1.3816 (19)
O3—N2	1.2346 (17)	C6—C7	1.4844 (18)
O4—N1	1.2304 (15)	C8—C9	1.439 (2)
O5—N1	1.2321 (15)	C9—H9a	0.93
O6—C7	1.2355 (16)	C9—C10	1.3449 (19)
O7—C7	1.3040 (17)	C10—C15	1.4889 (19)
O7—H7	1.0191 (9)	C11—C12	1.4936 (19)
O8—C8	1.2198 (17)	C12—H12a	0.98
N1—C4	1.4597 (18)	C12—C13	1.531 (2)
N2—C2	1.4576 (17)	C12—C14	1.5318 (19)
N3—C10	1.3963 (18)	C13—H13a	0.96
N3—C11	1.3236 (17)	C13—H13b	0.96
N3—H1n3	0.9729 (11)	C13—H13c	0.96
N4—C8	1.4152 (18)	C14—H14a	0.96
N4—C11	1.3303 (17)	C14—H14b	0.96
N4—H1n4	0.9085 (11)	C14—H14c	0.96
C1—C2	1.4259 (18)	C15—H15a	0.96
C1—C6	1.4349 (19)	C15—H15b	0.96
C2—C3	1.381 (2)	C15—H15c	0.96
C3—H3a	0.93	O1w—H2w1	0.9169 (10)
C3—C4	1.3763 (19)	O1w—H1w1	0.9146 (10)
			
C1—O1—H7	96.54 (8)	N4—C8—C9	113.60 (12)
C7—O7—H7	101.27 (9)	C8—C9—H9a	119.24
O4—N1—O5	124.04 (12)	C8—C9—C10	121.51 (13)
O4—N1—C4	118.12 (11)	H9a—C9—C10	119.24
O5—N1—C4	117.84 (11)	N3—C10—C9	118.95 (12)
O2—N2—O3	123.42 (12)	N3—C10—C15	116.00 (11)
O2—N2—C2	119.16 (12)	C9—C10—C15	125.06 (13)
O3—N2—C2	117.39 (12)	N3—C11—N4	118.76 (12)
C10—N3—C11	122.45 (11)	N3—C11—C12	120.45 (12)
C10—N3—H1n3	118.37 (11)	N4—C11—C12	120.78 (12)
C11—N3—H1n3	119.14 (11)	C11—C12—H12a	108.71
C8—N4—C11	124.69 (12)	C11—C12—C13	109.61 (13)
C8—N4—H1n4	116.55 (11)	C11—C12—C14	111.42 (10)
C11—N4—H1n4	118.72 (12)	H12a—C12—C13	108.78
O1—C1—C2	124.06 (12)	H12a—C12—C14	106.87
O1—C1—C6	120.40 (11)	C13—C12—C14	111.36 (12)
C2—C1—C6	115.53 (12)	C12—C13—H13a	109.47
N2—C2—C1	120.85 (12)	C12—C13—H13b	109.47
N2—C2—C3	116.56 (11)	C12—C13—H13c	109.47
C1—C2—C3	122.58 (12)	H13a—C13—H13b	109.47
C2—C3—H3a	120.52	H13a—C13—H13c	109.47
C2—C3—C4	118.97 (12)	H13b—C13—H13c	109.47
H3a—C3—C4	120.52	C12—C14—H14a	109.47
N1—C4—C3	118.77 (12)	C12—C14—H14b	109.47
N1—C4—C5	119.38 (12)	C12—C14—H14c	109.47
C3—C4—C5	121.85 (13)	H14a—C14—H14b	109.47
C4—C5—H5a	120.33	H14a—C14—H14c	109.47
C4—C5—C6	119.34 (12)	H14b—C14—H14c	109.47
H5a—C5—C6	120.33	C10—C15—H15a	109.47
C1—C6—C5	121.71 (12)	C10—C15—H15b	109.47
C1—C6—C7	119.30 (12)	C10—C15—H15c	109.47
C5—C6—C7	118.99 (12)	H15a—C15—H15b	109.47
O6—C7—O7	122.20 (12)	H15a—C15—H15c	109.47
O6—C7—C6	121.26 (12)	H15b—C15—H15c	109.47
O7—C7—C6	116.54 (12)	H2w1—O1w—H1w1	110.00 (10)
O8—C8—N4	119.14 (12)	O1—H7—O7	165.94 (7)
O8—C8—C9	127.26 (13)		

### 2-Isopropyl-6-methyl-4-oxo-3,4-dihydropyrimidin-1-ium 2-carboxy-4,6-dinitrophenolate monohydrate (VABZIJ) Hydrogen-bond geometry (Å, º)

**Table:**

*D*—H···*A*	*D*—H	H···*A*	*D*···*A*	*D*—H···*A*
C12—H12*a*···O7^i^	0.98	2.42	3.3068 (15)	151
N3—H1*n*3···O6^i^	0.9729 (11)	1.7539 (9)	2.7182 (14)	170.48 (8)
N4—H1*n*4···O1*w*	0.9085 (11)	1.8401 (10)	2.7348 (15)	167.76 (8)
O1*w*—H2*w*1···O1^ii^	0.9169 (10)	1.8895 (10)	2.7886 (14)	166.21 (6)
O1*w*—H1*w*1···O3^iii^	0.9146 (10)	2.0399 (10)	2.9352 (14)	165.84 (7)
O7—H7···O1	1.0191 (9)	1.4329 (9)	2.4340 (13)	165.94 (7)

Symmetry codes: (i) −*x*, −*y*+1, −*z*+1; (ii) *x*, *y*+1, *z*; (iii) −*x*+1, −*y*, −*z*.

### 1-Aza-8-azoniabicyclo[5.4.0]undec-7-ene 4-aminobenzoate (WADXOR) Crystal data

**Table:**

C_9_H_17_N_2_^+^·C_7_H_3_N_2_O_7_^−^	*F*(000) = 800
*M**_r_* = 380.35	*D*_x_ = 1.489 Mg m^−^^3^
Monoclinic, *P*2_1_/*n*	Mo *K*α radiation, λ = 0.71073 Å
Hall symbol: -P 2yabc	Cell parameters from 1891 reflections
*a* = 6.1537 (3) Å	θ = 3.5–26.6°
*b* = 19.1541 (14) Å	µ = 0.12 mm^−^^1^
*c* = 14.5527 (11) Å	*T* = 200 K
β = 98.343 (6)°	Needle, yellow
*V* = 1697.2 (2) Å^3^	0.30 × 0.13 × 0.10 mm
*Z* = 4	

### 1-Aza-8-azoniabicyclo[5.4.0]undec-7-ene 4-aminobenzoate (WADXOR) Data collection

**Table:**

Oxford Diffraction Gemini-S CCD-detector diffractometer	3339 independent reflections
Graphite monochromator	1976 reflections with *I* > 3σ(*I*)
Detector resolution: 16.067 pixels mm^-1^	*R*_int_ = 0.034
ω scans	θ_max_ = 26.0°, θ_min_ = 3.4°
Absorption correction: multi-scan (CrysAlis PRO; Agilent, 2014)	*h* = −7→7
*T*_min_ = 0.920, *T*_max_ = 0.990	*k* = −23→23
7800 measured reflections	*l* = −17→17

### 1-Aza-8-azoniabicyclo[5.4.0]undec-7-ene 4-aminobenzoate (WADXOR) Refinement

**Table:**

Refinement on *F*^2^	Secondary atom site location: difference Fourier map
Least-squares matrix: full	Hydrogen site location: difference Fourier map
*R*[*F*^2^ > 2σ(*F*^2^)] = 0.046	H atoms treated by a mixture of independent and constrained refinement
*wR*(*F*^2^) = 0.095	Weighting scheme based on measured s.u.'s *w* = 1/(σ^2^(*I*) + 0.0004*I*^2^)
*S* = 1.33	(Δ/σ)_max_ = 0.005
3339 reflections	Δρ_max_ = 0.36 e Å^−^^3^
268 parameters	Δρ_min_ = −0.24 e Å^−^^3^
2 restraints	Extinction correction: B-C type 1 Lorentzian isotropic (Becker & Coppens, 1974)
98 constraints	Extinction coefficient: 2200 (700)
Primary atom site location: structure-invariant direct methods	

### 1-Aza-8-azoniabicyclo[5.4.0]undec-7-ene 4-aminobenzoate (WADXOR) Special details

**Table:**

Geometry. Bond distances, angles etc. have been calculated using the rounded fractional coordinates. All su's are estimated from the variances of the (full) variance-covariance matrix. The cell esds are taken into account in the estimation of distances, angles and torsion angles
Refinement. Refinement of F^2^ against ALL reflections. The weighted R-factor wR and goodness of fit S are based on F^2^, conventional R-factors R are based on F, with F set to zero for negative F^2^. The threshold expression of F^2^ > 2sigma(F^2^) is used only for calculating R-factors(gt) etc. and is not relevant to the choice of reflections for refinement. R-factors based on F^2^ are statistically about twice as large as those based on F, and R- factors based on ALL data will be even larger.Number of fixed parameters : 10

### 1-Aza-8-azoniabicyclo[5.4.0]undec-7-ene 4-aminobenzoate (WADXOR) Fractional atomic coordinates and isotropic or equivalent isotropic displacement parameters (Å^2^)

**Table:**

	*x*	*y*	*z*	*U*_iso_*/*U*_eq_	Occ. (<1)
O2b	0.8418 (3)	0.56169 (11)	0.78950 (14)	0.0440 (8)	0.727 (4)
H61b	0.852107	0.560819	0.76707	0.0359*	0.273 (4)
O21b	0.7735 (8)	0.6569 (3)	0.4912 (4)	0.048 (2)	0.273 (4)
H6b	0.805712	0.644967	0.507207	0.0328*	0.727 (4)
O11b	0.5083 (2)	0.68884 (8)	0.59278 (11)	0.0444 (6)	
O12b	0.5447 (2)	0.64534 (8)	0.73586 (12)	0.0524 (7)	
O31b	1.1108 (3)	0.45706 (10)	0.81717 (14)	0.0829 (9)	
O32b	1.4073 (3)	0.47245 (10)	0.75770 (13)	0.0775 (8)	
O51b	1.3288 (3)	0.55869 (9)	0.44602 (12)	0.0603 (7)	
O52b	1.0707 (3)	0.63212 (10)	0.39870 (12)	0.0680 (8)	
N3b	1.2111 (3)	0.48284 (10)	0.76049 (14)	0.0472 (8)	
N5b	1.1654 (3)	0.59180 (10)	0.45701 (14)	0.0418 (7)	
C1b	0.8002 (3)	0.60956 (10)	0.63903 (15)	0.0264 (7)	
C2b	0.9062 (3)	0.56604 (11)	0.70954 (15)	0.0299 (7)	
C3b	1.0952 (3)	0.53063 (10)	0.69140 (15)	0.0309 (7)	
C4b	1.1814 (3)	0.53946 (10)	0.61042 (15)	0.0309 (7)	
C5b	1.0736 (3)	0.58234 (10)	0.54290 (14)	0.0271 (7)	
C6b	0.8810 (3)	0.61681 (10)	0.55538 (15)	0.0273 (7)	
C11b	0.6030 (3)	0.65080 (11)	0.65583 (18)	0.0351 (8)	
N1a	−0.1521 (2)	0.82026 (8)	0.63826 (12)	0.0293 (6)	
N8a	0.1721 (2)	0.76019 (9)	0.67283 (12)	0.0373 (7)	
C2a	−0.3261 (3)	0.85089 (11)	0.57022 (15)	0.0370 (8)	
C3a	−0.2602 (3)	0.91805 (11)	0.52679 (15)	0.0401 (8)	
C4a	−0.1188 (3)	0.90791 (11)	0.45061 (16)	0.0417 (8)	
C5a	0.0933 (3)	0.86805 (11)	0.48032 (14)	0.0401 (8)	
C6a	0.0611 (3)	0.79367 (11)	0.51423 (14)	0.0344 (8)	
C7a	0.0226 (3)	0.79087 (10)	0.61288 (14)	0.0268 (7)	
C9a	0.1394 (8)	0.7480 (3)	0.7694 (4)	0.0377 (17)	0.687 (4)
C10a	0.0234 (5)	0.8112 (2)	0.8006 (2)	0.0388 (12)	0.687 (4)
C11a	−0.1865 (3)	0.82334 (13)	0.73606 (16)	0.0375 (8)	
C13a	0.192 (2)	0.7733 (6)	0.7752 (10)	0.0377 (17)	0.313 (4)
C12a	−0.0453 (12)	0.7700 (5)	0.7962 (6)	0.0388 (12)	0.313 (4)
H4b	1.313123	0.516393	0.601144	0.0371*	
H8a	0.304017	0.743029	0.652706	0.073 (8)*	
H10a	0.119515	0.852593	0.800333	0.0466*	0.686
H21a	−0.458393	0.859249	0.600103	0.0444*	
H22a	−0.373911	0.816372	0.520773	0.0444*	
H31a	−0.393523	0.944805	0.502041	0.0481*	
H32a	−0.182575	0.948658	0.575716	0.0481*	
H41a	−0.084564	0.95395	0.425285	0.0501*	
H42a	−0.20539	0.883982	0.397103	0.0501*	
H51a	0.180506	0.866759	0.428286	0.0482*	
H52a	0.186838	0.894538	0.52943	0.0482*	
H61a	0.191356	0.765112	0.506561	0.0413*	
H62a	−0.063947	0.77162	0.474177	0.0413*	
H91a	0.046176	0.706149	0.772504	0.0452*	0.687 (4)
H92a	0.283833	0.74265	0.808712	0.0452*	0.687 (4)
H11a	−0.009873	0.803439	0.864351	0.0466*	0.686
H12a	−0.318 (2)	0.7965 (9)	0.7425 (13)	0.045*	
H13a	−0.230 (3)	0.8711 (6)	0.7485 (13)	0.045*	
H14a	−0.105082	0.7226	0.782436	0.0466*	0.313 (4)
H15a	−0.047311	0.780768	0.862598	0.0466*	0.313 (4)
H16a	0.27893	0.735519	0.809131	0.0452*	0.313 (4)
H17a	0.251697	0.820686	0.789237	0.0452*	0.313 (4)
H21b	0.621837	0.678052	0.538628	0.082 (11)*	0.273 (4)
H2b	0.702896	0.598291	0.774475	0.082 (11)*	0.727 (4)

### 1-Aza-8-azoniabicyclo[5.4.0]undec-7-ene 4-aminobenzoate (WADXOR) Atomic displacement parameters (Å^2^)

**Table:**

	*U*^11^	*U*^22^	*U*^33^	*U*^12^	*U*^13^	*U*^23^
O2b	0.0481 (12)	0.0570 (16)	0.0296 (15)	0.0087 (11)	0.0152 (10)	0.0091 (11)
O21b	0.045 (3)	0.053 (4)	0.046 (4)	0.006 (3)	0.006 (3)	0.023 (3)
O11b	0.0369 (8)	0.0391 (10)	0.0583 (12)	0.0136 (7)	0.0102 (8)	0.0073 (9)
O12b	0.0488 (9)	0.0643 (12)	0.0488 (12)	0.0091 (8)	0.0232 (8)	−0.0030 (9)
O31b	0.0796 (13)	0.0853 (16)	0.0753 (16)	−0.0256 (11)	−0.0175 (11)	0.0533 (13)
O32b	0.0728 (12)	0.0903 (16)	0.0642 (14)	0.0483 (11)	−0.0074 (10)	0.0050 (11)
O51b	0.0542 (10)	0.0650 (12)	0.0694 (13)	0.0077 (9)	0.0350 (9)	−0.0110 (10)
O52b	0.0926 (13)	0.0722 (14)	0.0455 (12)	0.0171 (11)	0.0314 (10)	0.0241 (10)
N3b	0.0575 (13)	0.0347 (13)	0.0432 (15)	0.0005 (11)	−0.0137 (11)	−0.0004 (10)
N5b	0.0476 (11)	0.0400 (13)	0.0414 (13)	−0.0046 (10)	0.0193 (10)	−0.0067 (10)
C1b	0.0265 (10)	0.0220 (11)	0.0303 (13)	−0.0029 (9)	0.0024 (9)	−0.0023 (10)
C2b	0.0337 (11)	0.0267 (12)	0.0294 (14)	−0.0061 (10)	0.0050 (10)	−0.0023 (10)
C3b	0.0361 (11)	0.0233 (12)	0.0304 (14)	0.0002 (9)	−0.0053 (10)	0.0025 (10)
C4b	0.0257 (10)	0.0240 (12)	0.0413 (15)	−0.0013 (9)	−0.0005 (10)	−0.0053 (11)
C5b	0.0295 (10)	0.0254 (12)	0.0274 (13)	−0.0052 (9)	0.0073 (9)	−0.0017 (10)
C6b	0.0298 (10)	0.0218 (12)	0.0290 (13)	−0.0028 (9)	−0.0003 (9)	0.0007 (10)
C11b	0.0297 (11)	0.0296 (13)	0.0464 (16)	−0.0042 (10)	0.0070 (11)	−0.0061 (12)
N1a	0.0249 (8)	0.0327 (11)	0.0305 (11)	0.0026 (8)	0.0041 (8)	0.0000 (9)
N8a	0.0332 (9)	0.0493 (12)	0.0291 (12)	0.0137 (9)	0.0037 (8)	0.0056 (9)
C2a	0.0243 (10)	0.0400 (14)	0.0451 (15)	0.0063 (10)	−0.0002 (10)	−0.0020 (12)
C3a	0.0374 (12)	0.0331 (14)	0.0465 (16)	0.0071 (10)	−0.0044 (11)	0.0000 (11)
C4a	0.0450 (12)	0.0397 (15)	0.0370 (15)	−0.0028 (11)	−0.0060 (11)	0.0087 (11)
C5a	0.0368 (11)	0.0526 (16)	0.0319 (14)	−0.0008 (11)	0.0079 (10)	0.0091 (12)
C6a	0.0355 (11)	0.0411 (15)	0.0262 (13)	0.0079 (10)	0.0032 (9)	−0.0057 (11)
C7a	0.0283 (10)	0.0224 (12)	0.0291 (13)	−0.0012 (9)	0.0025 (9)	−0.0030 (10)
C9a	0.040 (3)	0.041 (4)	0.0304 (18)	−0.001 (2)	−0.001 (2)	0.004 (3)
C10a	0.049 (2)	0.042 (2)	0.0270 (17)	−0.0043 (18)	0.0083 (15)	−0.0029 (19)
C11a	0.0367 (12)	0.0426 (15)	0.0364 (15)	0.0003 (11)	0.0161 (11)	−0.0056 (12)
C13a	0.040 (3)	0.041 (4)	0.0304 (18)	−0.001 (2)	−0.001 (2)	0.004 (3)
C12a	0.049 (2)	0.042 (2)	0.0270 (17)	−0.0043 (18)	0.0083 (15)	−0.0029 (19)

### 1-Aza-8-azoniabicyclo[5.4.0]undec-7-ene 4-aminobenzoate (WADXOR) Geometric parameters (Å, º)

**Table:**

O2b—C2b	1.285 (3)	C2a—H22a	0.99
O2b—H2b	1.103 (2)	C3a—C4a	1.518 (3)
H61b—C2b	0.95	C3a—H31a	0.99
O21b—C6b	1.311 (6)	C3a—H32a	0.99
O21b—H21b	1.303 (6)	C4a—C5a	1.520 (3)
H6b—C6b	0.95	C4a—H41a	0.99
O11b—C11b	1.248 (3)	C4a—H42a	0.99
O11b—H21b	1.1454 (16)	C5a—C6a	1.530 (3)
O12b—C11b	1.272 (3)	C5a—H51a	0.99
O12b—H2b	1.3846 (15)	C5a—H52a	0.99
O31b—N3b	1.205 (3)	C6a—C7a	1.490 (3)
O32b—N3b	1.230 (3)	C6a—H61a	0.99
O51b—N5b	1.219 (3)	C6a—H62a	0.99
O52b—N5b	1.230 (3)	C9a—C10a	1.508 (6)
N3b—C3b	1.466 (3)	C9a—C13a	0.581 (13)
N5b—C5b	1.456 (3)	C9a—C12a	1.323 (10)
C1b—C2b	1.407 (3)	C9a—H91a	0.99
C1b—C6b	1.387 (3)	C9a—H92a	0.99
C1b—C11b	1.497 (3)	C9a—H16a	0.9922
C2b—C3b	1.404 (3)	C10a—C11a	1.501 (4)
C3b—C4b	1.371 (3)	C10a—C13a	1.358 (14)
C4b—C5b	1.375 (3)	C10a—H10a	0.99
C4b—H4b	0.95	C10a—H11a	0.99
C5b—C6b	1.392 (3)	C10a—H15a	1.207
N1a—C2a	1.471 (2)	C11a—C12a	1.530 (8)
N1a—C7a	1.313 (2)	C11a—H12a	0.977 (15)
N1a—C11a	1.470 (3)	C11a—H13a	0.978 (13)
N8a—C7a	1.311 (2)	C13a—C12a	1.534 (16)
N8a—C9a	1.467 (6)	C13a—H92a	0.9083
N8a—C13a	1.498 (15)	C13a—H16a	0.99
N8a—H8a	0.9604 (17)	C13a—H17a	0.99
C2a—C3a	1.514 (3)	C12a—H14a	0.99
C2a—H21a	0.99	C12a—H15a	0.99
			
H6b—O21b—H21b	102.88	N8a—C9a—C13a	81.6 (16)
C11b—O12b—H2b	98.61 (14)	N8a—C9a—C12a	118.3 (5)
O31b—N3b—O32b	124.0 (2)	N8a—C9a—H91a	109.47
O31b—N3b—C3b	118.6 (2)	N8a—C9a—H92a	109.47
O32b—N3b—C3b	117.4 (2)	N8a—C9a—H14a	114.78
O51b—N5b—O52b	123.6 (2)	N8a—C9a—H16a	111.65
O51b—N5b—C5b	118.57 (18)	C10a—C9a—H91a	109.47
O52b—N5b—C5b	117.83 (18)	C10a—C9a—H92a	109.47
C2b—C1b—C6b	120.82 (17)	C13a—C9a—C12a	99.9 (15)
C2b—C1b—C11b	119.6 (2)	C13a—C9a—H91a	168.74
C6b—C1b—C11b	119.51 (18)	C13a—C9a—H14a	137.56
O2b—C2b—C1b	121.84 (19)	C12a—C9a—H92a	126.91
O2b—C2b—C3b	120.77 (19)	C12a—C9a—H16a	127.82
H61b—C2b—C1b	121.37	H91a—C9a—H92a	111.59
H61b—C2b—C3b	121.37	H14a—C9a—H16a	126.91
C1b—C2b—C3b	117.3 (2)	H14a—C9a—H17a	129.78
N3b—C3b—C2b	120.4 (2)	H16a—C9a—H17a	77.55
N3b—C3b—C4b	117.12 (18)	C9a—C10a—C11a	109.8 (3)
C2b—C3b—C4b	122.44 (18)	C11a—C10a—C13a	122.2 (6)
C3b—C4b—C5b	118.75 (18)	C11a—C10a—H10a	109.47
C3b—C4b—H4b	120.62	C11a—C10a—H11a	109.47
C5b—C4b—H4b	120.62	C11a—C10a—H17a	132.02
N5b—C5b—C4b	118.74 (17)	C13a—C10a—H11a	116.43
N5b—C5b—C6b	119.80 (17)	C13a—C10a—H15a	108.54
C4b—C5b—C6b	121.5 (2)	C12a—C10a—H17a	124.07
O21b—C6b—C1b	118.4 (3)	H10a—C10a—H11a	109.16
O21b—C6b—C5b	122.4 (3)	H10a—C10a—H15a	132.06
H6b—C6b—C1b	120.41	H11a—C10a—H17a	117.48
H6b—C6b—C5b	120.41	H15a—C10a—H17a	127.34
C1b—C6b—C5b	119.17 (18)	N1a—C11a—C10a	111.6 (2)
O11b—C11b—O12b	123.9 (2)	N1a—C11a—C12a	112.2 (4)
O11b—C11b—C1b	119.4 (2)	N1a—C11a—H12a	108.1 (11)
O12b—C11b—C1b	116.70 (19)	N1a—C11a—H13a	107.3 (12)
C2a—N1a—C7a	121.83 (17)	C10a—C11a—H12a	120.7 (10)
C2a—N1a—C11a	116.29 (16)	C10a—C11a—H13a	105.2 (10)
C7a—N1a—C11a	121.87 (16)	C12a—C11a—H13a	131.8 (11)
C7a—N8a—C9a	121.9 (2)	H12a—C11a—H13a	102.9 (15)
C7a—N8a—C13a	122.3 (5)	N8a—C13a—C9a	75.8 (15)
C7a—N8a—H8a	119.53 (19)	N8a—C13a—C10a	114.0 (9)
C9a—N8a—C13a	22.6 (5)	N8a—C13a—C12a	104.5 (8)
C9a—N8a—H8a	118.5 (2)	N8a—C13a—H10a	113.12
C13a—N8a—H8a	114.3 (5)	N8a—C13a—H92a	112.32
N1a—C2a—C3a	114.01 (15)	N8a—C13a—H16a	109.47
N1a—C2a—H21a	109.47	N8a—C13a—H17a	109.47
N1a—C2a—H22a	109.47	C9a—C13a—H10a	130.08
C3a—C2a—H21a	109.47	C9a—C13a—H17a	167.66
C3a—C2a—H22a	109.47	C10a—C13a—H92a	129.79
H21a—C2a—H22a	104.52	C10a—C13a—H16a	129.35
C2a—C3a—C4a	114.39 (18)	C12a—C13a—H92a	113.84
C2a—C3a—H31a	109.47	C12a—C13a—H16a	109.47
C2a—C3a—H32a	109.47	C12a—C13a—H17a	109.47
C4a—C3a—H31a	109.47	H10a—C13a—H91a	126.53
C4a—C3a—H32a	109.47	H10a—C13a—H92a	130.35
H31a—C3a—H32a	104.05	H10a—C13a—H16a	135.32
C3a—C4a—C5a	114.58 (18)	H92a—C13a—H17a	107.21
C3a—C4a—H41a	109.47	H16a—C13a—H17a	114.05
C3a—C4a—H42a	109.47	C9a—C12a—C11a	119.1 (6)
C5a—C4a—H41a	109.47	C9a—C12a—H11a	111.16
C5a—C4a—H42a	109.47	C9a—C12a—H15a	119.13
H41a—C4a—H42a	103.83	C10a—C12a—H91a	125.59
C4a—C5a—C6a	114.44 (16)	C10a—C12a—H14a	169.66
C4a—C5a—H51a	109.47	C11a—C12a—C13a	109.6 (7)
C4a—C5a—H52a	109.47	C11a—C12a—H91a	130.75
C6a—C5a—H51a	109.47	C11a—C12a—H14a	109.47
C6a—C5a—H52a	109.47	C11a—C12a—H15a	109.47
H51a—C5a—H52a	104	C13a—C12a—H14a	109.47
C5a—C6a—C7a	112.99 (17)	C13a—C12a—H15a	109.47
C5a—C6a—H61a	109.47	H91a—C12a—H11a	130.35
C5a—C6a—H62a	109.47	H91a—C12a—H15a	118.71
C7a—C6a—H61a	109.47	H11a—C12a—H14a	133.54
C7a—C6a—H62a	109.47	H14a—C12a—H15a	109.31
H61a—C6a—H62a	105.71	O21b—H21b—O11b	167.3 (3)
N1a—C7a—N8a	121.93 (19)	H6b—H21b—O11b	152.98
N1a—C7a—C6a	120.45 (16)	O2b—H2b—O12b	166.81 (13)
N8a—C7a—C6a	117.59 (17)	H61b—H2b—O12b	150.18
N8a—C9a—C10a	107.3 (3)		

### 1-Aza-8-azoniabicyclo[5.4.0]undec-7-ene 4-aminobenzoate (WADXOR) Hydrogen-bond geometry (Å, º)

**Table:**

*D*—H···*A*	*D*—H	H···*A*	*D*···*A*	*D*—H···*A*
N8*a*—H8*a*···O11*b*	0.9604 (17)	1.9329 (15)	2.864 (2)	162.83 (11)
C10*a*—H10*a*···O32*b*^i^	0.99	2.44	3.248 (4)	138
C2*a*—H21*a*···O31*b*^ii^	0.99	2.48	3.275 (3)	137
C6*a*—H62*a*···O21*b*^iii^	0.99	2.44	3.152 (6)	128
C10*a*—H11*a*···O21*b*^iv^	0.99	2.47	3.033 (7)	116
O21*b*—H21*b*···O11*b*	1.303 (6)	1.1454 (16)	2.433 (6)	167.3 (3)
O11*b*—H21*b*···O21*b*	1.1454 (16)	1.303 (6)	2.433 (6)	167.3 (3)
O12*b*—H2*b*···O2*b*	1.3846 (15)	1.103 (2)	2.471 (2)	166.81 (13)

Symmetry codes: (i) −*x*+3/2, *y*+1/2, −*z*+3/2; (ii) −*x*+1/2, *y*+1/2, −*z*+3/2; (iii) *x*−1, *y*, *z*; (iv) *x*−1/2, −*y*+3/2, *z*+1/2.

### 4-(Diphenylmethyl)-1-(3-phenylprop-2-en-1-yl)piperazin-1-ium 2-carboxy-4,6-dinitrophenolate (YAXPOE) Crystal data

**Table:**

C_26_H_29_N_2_^+^·C_7_H_3_N_2_O_7_^−^	*F*(000) = 1256
*M**_r_* = 596.63	*D*_x_ = 1.341 Mg m^−^^3^
Monoclinic, *P*2_1_/*c*	Melting point: 383 K
Hall symbol: -P 2ybc	Mo *K*α radiation, λ = 0.71073 Å
*a* = 14.5648 (3) Å	Cell parameters from 9909 reflections
*b* = 12.9374 (3) Å	θ = 2.3–28.3°
*c* = 16.1619 (3) Å	µ = 0.10 mm^−^^1^
β = 103.900 (1)°	*T* = 200 K
*V* = 2956.22 (11) Å^3^	Block, yellow
*Z* = 4	0.51 × 0.26 × 0.17 mm

### 4-(Diphenylmethyl)-1-(3-phenylprop-2-en-1-yl)piperazin-1-ium 2-carboxy-4,6-dinitrophenolate (YAXPOE) Data collection

**Table:**

Bruker APEXII CCD diffractometer	7344 independent reflections
Radiation source: fine-focus sealed tube	5724 reflections with *I* > 3σ(*I*)
Graphite monochromator	*R*_int_ = 0.015
φ and ω scans	θ_max_ = 28.3°, θ_min_ = 2.0°
Absorption correction: multi-scan (SADABS; Bruker, 2008)	*h* = −19→19
*T*_min_ = 0.932, *T*_max_ = 1.000	*k* = −17→17
29552 measured reflections	*l* = −21→15

### 4-(Diphenylmethyl)-1-(3-phenylprop-2-en-1-yl)piperazin-1-ium 2-carboxy-4,6-dinitrophenolate (YAXPOE) Refinement

**Table:**

Refinement on *F*^2^	Primary atom site location: structure-invariant direct methods
Least-squares matrix: full	Secondary atom site location: difference Fourier map
*R*[*F* > 3σ(*F*)] = 0.054	Hydrogen site location: difference Fourier map
*wR*(*F*) = 0.190	H atoms treated by a mixture of independent and constrained refinement
*S* = 1.80	Weighting scheme based on measured s.u.'s *w* = 1/(σ^2^(*I*) + 0.0063999998*I*^2^)
7344 reflections	(Δ/σ)_max_ = 0.044
399 parameters	Δρ_max_ = 0.63 e Å^−^^3^
0 restraints	Δρ_min_ = −0.28 e Å^−^^3^
120 constraints	

### 4-(Diphenylmethyl)-1-(3-phenylprop-2-en-1-yl)piperazin-1-ium 2-carboxy-4,6-dinitrophenolate (YAXPOE) Special details

**Table:**

Refinement. Number of fixed parameters 6

### 4-(Diphenylmethyl)-1-(3-phenylprop-2-en-1-yl)piperazin-1-ium 2-carboxy-4,6-dinitrophenolate (YAXPOE) Fractional atomic coordinates and isotropic or equivalent isotropic displacement parameters (Å^2^)

**Table:**

	*x*	*y*	*z*	*U*_iso_*/*U*_eq_	
N1	0.25627 (8)	0.71181 (9)	0.31343 (7)	0.0246 (3)	
H71	0.30128	0.669474	0.349979	0.035 (4)*	
N2	0.10023 (8)	0.56683 (9)	0.26575 (7)	0.0233 (3)	
C1	0.29865 (11)	0.95151 (12)	0.41661 (10)	0.0334 (5)	
H1a	0.245898	0.980932	0.377422	0.0401*	
C2	0.33463 (11)	0.86695 (12)	0.39170 (10)	0.0322 (5)	
H2	0.386765	0.835478	0.430202	0.0386*	
C3	0.29839 (12)	0.81754 (12)	0.30656 (10)	0.0333 (5)	
H3a	0.350521	0.811391	0.27725	0.04*	
H3b	0.250048	0.862741	0.270514	0.04*	
C4	0.17112 (10)	0.71678 (11)	0.34935 (9)	0.0281 (4)	
H4a	0.123241	0.762704	0.313566	0.0337*	
H4b	0.189039	0.745897	0.40767	0.0337*	
C5	0.12943 (10)	0.61031 (11)	0.35226 (9)	0.0271 (4)	
H5a	0.07387	0.614805	0.377337	0.0326*	
H5b	0.177161	0.564522	0.388386	0.0326*	
C6	0.18571 (10)	0.55563 (11)	0.23345 (9)	0.0262 (4)	
H6a	0.231171	0.509606	0.271816	0.0315*	
H6b	0.168931	0.5233	0.176272	0.0315*	
C7	0.23149 (10)	0.65944 (12)	0.22789 (9)	0.0282 (4)	
H7a	0.289537	0.649813	0.207068	0.0338*	
H7b	0.187639	0.703827	0.186493	0.0338*	
C8	0.05326 (9)	0.46528 (10)	0.26756 (8)	0.0230 (4)	
H8	0.099282	0.417266	0.304542	0.0276*	
C11	0.33145 (11)	1.00535 (12)	0.49876 (10)	0.0326 (5)	
C12	0.41578 (13)	0.97941 (14)	0.55685 (10)	0.0391 (5)	
H12	0.454927	0.926517	0.543205	0.047*	
C13	0.44278 (15)	1.03053 (17)	0.63457 (11)	0.0502 (6)	
H13	0.499799	1.011658	0.674094	0.0603*	
C14	0.38752 (17)	1.10810 (17)	0.65451 (12)	0.0554 (7)	
H14	0.40688	1.143158	0.707495	0.0665*	
C15	0.30389 (15)	1.13552 (16)	0.59798 (13)	0.0516 (7)	
H15	0.265849	1.18931	0.61197	0.0619*	
C16	0.27570 (13)	1.08400 (13)	0.52053 (11)	0.0397 (5)	
H16	0.217882	1.102436	0.481949	0.0476*	
C21	−0.03166 (9)	0.47504 (10)	0.30647 (8)	0.0240 (4)	
C22	−0.04272 (11)	0.40385 (12)	0.36763 (10)	0.0315 (5)	
H22	0.003276	0.351198	0.385315	0.0378*	
C23	−0.12077 (12)	0.40899 (13)	0.40333 (11)	0.0388 (5)	
H23	−0.127745	0.359826	0.445056	0.0466*	
C24	−0.18805 (12)	0.48544 (13)	0.37818 (11)	0.0385 (5)	
H24	−0.241677	0.488365	0.401911	0.0461*	
C25	−0.17687 (11)	0.55719 (12)	0.31859 (11)	0.0351 (5)	
H25	−0.222711	0.610174	0.301738	0.0421*	
C26	−0.09910 (10)	0.55304 (11)	0.28271 (9)	0.0286 (4)	
H26	−0.091899	0.603396	0.241958	0.0344*	
C31	0.02520 (10)	0.41880 (11)	0.17810 (9)	0.0248 (4)	
C32	0.06690 (11)	0.32720 (12)	0.16027 (10)	0.0331 (5)	
H32	0.112496	0.2938	0.204043	0.0397*	
C33	0.04254 (13)	0.28416 (13)	0.07918 (11)	0.0392 (5)	
H33	0.071842	0.221893	0.067798	0.047*	
C34	−0.02351 (12)	0.33108 (13)	0.01557 (10)	0.0379 (5)	
H34	−0.039638	0.301886	−0.039979	0.0455*	
C35	−0.06672 (12)	0.42142 (14)	0.03267 (10)	0.0385 (5)	
H35	−0.113709	0.453238	−0.01092	0.0461*	
C36	−0.04162 (11)	0.46550 (12)	0.11316 (10)	0.0321 (5)	
H36	−0.070483	0.528311	0.123929	0.0386*	
O1	0.63327 (8)	0.37352 (10)	0.53505 (8)	0.0392 (4)	
O2	0.58197 (10)	0.44006 (14)	0.67133 (9)	0.0614 (6)	
O3	0.43608 (10)	0.48129 (12)	0.65684 (9)	0.0549 (5)	
O4	0.19464 (9)	0.32143 (13)	0.44743 (10)	0.0569 (5)	
O5	0.22764 (11)	0.24248 (13)	0.34091 (10)	0.0631 (6)	
O6	0.54745 (10)	0.23529 (12)	0.30214 (8)	0.0518 (5)	
O7	0.66627 (9)	0.28940 (12)	0.40564 (9)	0.0507 (5)	
N3	0.49861 (10)	0.43835 (11)	0.63197 (9)	0.0379 (4)	
N4	0.25133 (10)	0.29121 (12)	0.40756 (10)	0.0428 (5)	
C9	0.57563 (13)	0.27360 (13)	0.37128 (12)	0.0398 (6)	
C41	0.54458 (10)	0.35627 (11)	0.50735 (10)	0.0298 (4)	
C42	0.50987 (11)	0.30564 (12)	0.42622 (10)	0.0314 (5)	
C43	0.41587 (11)	0.28489 (12)	0.39522 (10)	0.0329 (5)	
H43	0.395087	0.249901	0.342348	0.0394*	
C44	0.35106 (10)	0.31453 (12)	0.44039 (10)	0.0313 (4)	
C45	0.37850 (10)	0.36477 (12)	0.51759 (10)	0.0305 (4)	
H45	0.332901	0.38543	0.547596	0.0366*	
C46	0.47336 (11)	0.38456 (11)	0.55054 (9)	0.0290 (4)	
H7	0.676841	0.321683	0.458063	0.030 (4)*	

### 4-(Diphenylmethyl)-1-(3-phenylprop-2-en-1-yl)piperazin-1-ium 2-carboxy-4,6-dinitrophenolate (YAXPOE) Atomic displacement parameters (Å^2^)

**Table:**

	*U*^11^	*U*^22^	*U*^33^	*U*^12^	*U*^13^	*U*^23^
N1	0.0221 (6)	0.0282 (6)	0.0236 (5)	−0.0039 (4)	0.0055 (4)	−0.0028 (4)
N2	0.0209 (5)	0.0268 (6)	0.0227 (5)	−0.0035 (4)	0.0063 (4)	−0.0032 (4)
C1	0.0300 (8)	0.0358 (8)	0.0314 (7)	−0.0037 (6)	0.0017 (6)	−0.0003 (6)
C2	0.0301 (7)	0.0308 (7)	0.0334 (7)	−0.0076 (6)	0.0033 (6)	0.0004 (6)
C3	0.0391 (8)	0.0304 (7)	0.0318 (7)	−0.0113 (6)	0.0111 (6)	−0.0022 (6)
C4	0.0216 (6)	0.0331 (7)	0.0302 (7)	−0.0026 (5)	0.0075 (5)	−0.0076 (5)
C5	0.0248 (7)	0.0337 (7)	0.0241 (6)	−0.0051 (5)	0.0082 (5)	−0.0053 (5)
C6	0.0232 (6)	0.0304 (7)	0.0265 (6)	−0.0032 (5)	0.0088 (5)	−0.0047 (5)
C7	0.0285 (7)	0.0344 (7)	0.0223 (6)	−0.0060 (6)	0.0073 (5)	−0.0045 (5)
C8	0.0197 (6)	0.0247 (6)	0.0245 (6)	0.0003 (5)	0.0054 (5)	0.0010 (5)
C11	0.0357 (8)	0.0306 (7)	0.0307 (7)	−0.0080 (6)	0.0064 (6)	0.0006 (6)
C12	0.0398 (9)	0.0415 (9)	0.0337 (8)	−0.0067 (7)	0.0042 (7)	0.0007 (7)
C13	0.0523 (11)	0.0604 (12)	0.0319 (8)	−0.0142 (9)	−0.0018 (8)	0.0005 (8)
C14	0.0691 (14)	0.0629 (13)	0.0354 (9)	−0.0230 (10)	0.0149 (9)	−0.0159 (8)
C15	0.0584 (12)	0.0455 (10)	0.0562 (11)	−0.0106 (9)	0.0242 (10)	−0.0151 (8)
C16	0.0392 (9)	0.0350 (8)	0.0444 (9)	−0.0049 (7)	0.0090 (7)	−0.0039 (7)
C21	0.0213 (6)	0.0250 (6)	0.0255 (6)	−0.0020 (5)	0.0050 (5)	−0.0024 (5)
C22	0.0311 (7)	0.0329 (8)	0.0325 (7)	0.0030 (6)	0.0114 (6)	0.0046 (6)
C23	0.0426 (9)	0.0390 (9)	0.0413 (9)	0.0005 (7)	0.0228 (7)	0.0058 (7)
C24	0.0344 (8)	0.0391 (8)	0.0493 (9)	−0.0028 (6)	0.0245 (7)	−0.0078 (7)
C25	0.0278 (7)	0.0336 (8)	0.0447 (9)	0.0044 (6)	0.0104 (6)	−0.0058 (6)
C26	0.0269 (7)	0.0271 (7)	0.0323 (7)	0.0015 (5)	0.0079 (6)	0.0002 (5)
C31	0.0226 (6)	0.0253 (6)	0.0282 (6)	−0.0049 (5)	0.0093 (5)	−0.0016 (5)
C32	0.0327 (8)	0.0296 (7)	0.0367 (8)	0.0017 (6)	0.0076 (6)	−0.0021 (6)
C33	0.0437 (9)	0.0299 (8)	0.0463 (9)	−0.0014 (7)	0.0154 (8)	−0.0097 (7)
C34	0.0452 (9)	0.0381 (9)	0.0313 (8)	−0.0104 (7)	0.0109 (7)	−0.0090 (6)
C35	0.0387 (9)	0.0451 (9)	0.0283 (7)	−0.0015 (7)	0.0017 (7)	−0.0024 (7)
C36	0.0316 (8)	0.0337 (8)	0.0310 (7)	0.0020 (6)	0.0073 (6)	−0.0020 (6)
O1	0.0242 (6)	0.0466 (7)	0.0449 (6)	−0.0022 (5)	0.0046 (5)	0.0065 (5)
O2	0.0364 (7)	0.0954 (12)	0.0457 (8)	0.0021 (7)	−0.0029 (6)	−0.0243 (7)
O3	0.0459 (8)	0.0698 (9)	0.0453 (7)	0.0153 (7)	0.0038 (6)	−0.0171 (6)
O4	0.0239 (6)	0.0732 (10)	0.0713 (9)	0.0049 (6)	0.0067 (6)	−0.0008 (8)
O5	0.0465 (8)	0.0774 (11)	0.0531 (8)	−0.0103 (7)	−0.0120 (7)	−0.0150 (7)
O6	0.0595 (9)	0.0612 (9)	0.0400 (7)	0.0066 (7)	0.0226 (6)	−0.0026 (6)
O7	0.0391 (7)	0.0619 (9)	0.0567 (8)	0.0007 (6)	0.0223 (6)	−0.0035 (6)
N3	0.0355 (7)	0.0405 (8)	0.0347 (7)	0.0038 (6)	0.0023 (6)	−0.0023 (6)
N4	0.0303 (7)	0.0466 (8)	0.0442 (8)	−0.0012 (6)	−0.0056 (6)	0.0040 (6)
C9	0.0393 (9)	0.0352 (8)	0.0491 (10)	0.0046 (7)	0.0190 (8)	0.0095 (7)
C41	0.0232 (7)	0.0277 (7)	0.0368 (7)	0.0028 (5)	0.0038 (6)	0.0099 (6)
C42	0.0330 (8)	0.0293 (7)	0.0345 (7)	0.0049 (6)	0.0133 (6)	0.0074 (6)
C43	0.0370 (8)	0.0322 (8)	0.0284 (7)	0.0011 (6)	0.0059 (6)	0.0030 (6)
C44	0.0238 (7)	0.0334 (8)	0.0333 (7)	0.0012 (5)	0.0000 (6)	0.0037 (6)
C45	0.0255 (7)	0.0325 (7)	0.0333 (7)	0.0060 (6)	0.0067 (6)	0.0030 (6)
C46	0.0282 (7)	0.0282 (7)	0.0282 (7)	0.0018 (5)	0.0017 (6)	0.0016 (5)

### 4-(Diphenylmethyl)-1-(3-phenylprop-2-en-1-yl)piperazin-1-ium 2-carboxy-4,6-dinitrophenolate (YAXPOE) Geometric parameters (Å, º)

**Table:**

N1—H71	0.9448 (11)	C16—H16	0.95
N1—C3	1.5141 (19)	C21—C22	1.388 (2)
N1—C4	1.492 (2)	C21—C26	1.3962 (19)
N1—C7	1.5036 (18)	C22—H22	0.95
N2—C5	1.4718 (17)	C22—C23	1.395 (3)
N2—C6	1.4682 (19)	C23—H23	0.95
N2—C8	1.4848 (17)	C23—C24	1.383 (2)
C1—H1a	0.95	C24—H24	0.95
C1—C2	1.317 (2)	C24—C25	1.375 (3)
C1—C11	1.474 (2)	C25—H25	0.95
C2—H2	0.95	C25—C26	1.392 (2)
C2—C3	1.494 (2)	C26—H26	0.95
C3—H3a	0.99	C31—C32	1.393 (2)
C3—H3b	0.99	C31—C36	1.3867 (19)
H3a—H3b	1.5869	C32—H32	0.95
C4—H4a	0.99	C32—C33	1.389 (2)
C4—H4b	0.99	C33—H33	0.95
C4—C5	1.511 (2)	C33—C34	1.370 (2)
H4a—H4b	1.6058	C34—H34	0.95
C5—H5a	0.99	C34—C35	1.386 (3)
C5—H5b	0.99	C35—H35	0.95
H5a—H5b	1.6091	C35—C36	1.387 (2)
C6—H6a	0.99	C36—H36	0.95
C6—H6b	0.99	O1—C41	1.2810 (17)
C6—C7	1.512 (2)	O2—N3	1.2281 (18)
H6a—H6b	1.6016	O3—N3	1.215 (2)
C7—H7a	0.99	O4—N4	1.227 (2)
C7—H7b	0.99	O5—N4	1.224 (2)
H7a—H7b	1.6014	O6—C9	1.201 (2)
C8—H8	1	O7—C9	1.319 (2)
C8—C21	1.522 (2)	O7—H7	0.9238 (15)
C8—C31	1.5282 (19)	N3—C46	1.456 (2)
C11—C12	1.396 (2)	N4—C44	1.453 (2)
C11—C16	1.399 (3)	C9—C42	1.512 (3)
C12—H12	0.95	C41—C42	1.444 (2)
C12—C13	1.390 (2)	C41—C46	1.430 (2)
C13—H13	0.95	C42—C43	1.367 (2)
C13—C14	1.372 (3)	C43—H43	0.95
C14—H14	0.95	C43—C44	1.379 (2)
C14—C15	1.382 (3)	C44—C45	1.378 (2)
C15—H15	0.95	C45—H45	0.95
C15—C16	1.390 (3)	C45—C46	1.380 (2)
			
H71—N1—C3	109.70 (10)	C15—C16—H16	119.61
H71—N1—C4	107.37 (11)	C8—C21—C22	118.95 (12)
H71—N1—C7	106.90 (11)	C8—C21—C26	122.30 (13)
C3—N1—C4	112.27 (12)	C22—C21—C26	118.75 (14)
C3—N1—C7	110.59 (11)	C21—C22—H22	119.72
C4—N1—C7	109.82 (10)	C21—C22—C23	120.56 (14)
C5—N2—C6	107.42 (10)	H22—C22—C23	119.72
C5—N2—C8	110.41 (11)	C22—C23—H23	119.9
C6—N2—C8	110.71 (11)	C22—C23—C24	120.20 (16)
H1a—C1—C2	116.52	H23—C23—C24	119.9
H1a—C1—C11	116.52	C23—C24—H24	120.2
C2—C1—C11	126.96 (14)	C23—C24—C25	119.60 (17)
C1—C2—H2	118.03	H24—C24—C25	120.2
C1—C2—C3	123.95 (13)	C24—C25—H25	119.64
H2—C2—C3	118.03	C24—C25—C26	120.72 (15)
N1—C3—C2	112.26 (13)	H25—C25—C26	119.64
N1—C3—H3a	109.47	C21—C26—C25	120.16 (14)
N1—C3—H3b	109.47	C21—C26—H26	119.92
C2—C3—H3a	109.47	C25—C26—H26	119.92
C2—C3—H3b	109.47	C8—C31—C32	119.98 (11)
H3a—C3—H3b	106.54	C8—C31—C36	121.61 (13)
N1—C4—H4a	109.47	C32—C31—C36	118.41 (13)
N1—C4—H4b	109.47	C31—C32—H32	119.64
N1—C4—C5	110.53 (12)	C31—C32—C33	120.73 (13)
H4a—C4—H4b	108.39	H32—C32—C33	119.64
H4a—C4—C5	109.47	C32—C33—H33	119.85
H4b—C4—C5	109.47	C32—C33—C34	120.30 (16)
N2—C5—C4	110.21 (12)	H33—C33—C34	119.85
N2—C5—H5a	109.47	C33—C34—H34	120.18
N2—C5—H5b	109.47	C33—C34—C35	119.65 (15)
C4—C5—H5a	109.47	H34—C34—C35	120.18
C4—C5—H5b	109.47	C34—C35—H35	119.87
H5a—C5—H5b	108.72	C34—C35—C36	120.25 (14)
N2—C6—H6a	109.47	H35—C35—C36	119.88
N2—C6—H6b	109.47	C31—C36—C35	120.65 (15)
N2—C6—C7	110.93 (12)	C31—C36—H36	119.67
H6a—C6—H6b	107.97	C35—C36—H36	119.68
H6a—C6—C7	109.47	C41—O1—H7	101.64 (10)
H6b—C6—C7	109.47	C9—O7—H7	112.68 (16)
N1—C7—C6	110.95 (12)	O2—N3—O3	123.16 (15)
N1—C7—H7a	109.47	O2—N3—C46	118.72 (15)
N1—C7—H7b	109.47	O3—N3—C46	118.10 (13)
C6—C7—H7a	109.47	O4—N4—O5	122.91 (15)
C6—C7—H7b	109.47	O4—N4—C44	118.79 (14)
H7a—C7—H7b	107.95	O5—N4—C44	118.29 (16)
N2—C8—H8	108.39	O6—C9—O7	122.64 (19)
N2—C8—C21	111.04 (11)	O6—C9—C42	122.44 (16)
N2—C8—C31	110.45 (11)	O7—C9—C42	114.91 (15)
H8—C8—C21	107.41	O1—C41—C42	120.00 (15)
H8—C8—C31	108.03	O1—C41—C46	125.00 (14)
C21—C8—C31	111.38 (10)	C42—C41—C46	115.00 (13)
C1—C11—C12	122.33 (15)	C9—C42—C41	121.64 (14)
C1—C11—C16	119.24 (13)	C9—C42—C43	116.80 (14)
C12—C11—C16	118.42 (15)	C41—C42—C43	121.55 (16)
C11—C12—H12	119.81	C42—C43—H43	119.87
C11—C12—C13	120.37 (17)	C42—C43—C44	120.27 (14)
H12—C12—C13	119.81	H43—C43—C44	119.87
C12—C13—H13	119.82	N4—C44—C43	120.01 (14)
C12—C13—C14	120.37 (17)	N4—C44—C45	118.40 (15)
H13—C13—C14	119.82	C43—C44—C45	121.59 (13)
C13—C14—H14	119.8	C44—C45—H45	120.57
C13—C14—C15	120.40 (18)	C44—C45—C46	118.86 (15)
H14—C14—C15	119.8	H45—C45—C46	120.57
C14—C15—H15	120.17	N3—C46—C41	120.55 (13)
C14—C15—C16	119.7 (2)	N3—C46—C45	116.73 (14)
H15—C15—C16	120.17	C41—C46—C45	122.71 (13)
C11—C16—C15	120.77 (16)	O1—H7—O7	148.96 (9)
C11—C16—H16	119.61		

### 4-(Diphenylmethyl)-1-(3-phenylprop-2-en-1-yl)piperazin-1-ium 2-carboxy-4,6-dinitrophenolate (YAXPOE) Hydrogen-bond geometry (Å, º)

**Table:**

*D*—H···*A*	*D*—H	H···*A*	*D*···*A*	*D*—H···*A*
N1—H71···O1^i^	0.9448 (11)	1.9544 (11)	2.8126 (15)	150.01 (8)
N1—H71···O2^i^	0.9448 (11)	2.3020 (16)	3.032 (2)	133.62 (8)
C3—H3*a*···O6^ii^	0.99	2.40	3.340 (2)	159
C5—H5*a*···C21	0.99	2.47	2.8777 (19)	104
C6—H6*b*···C31	0.99	2.50	2.8974 (19)	104
O7—H7···O1	0.9238 (15)	1.6675 (13)	2.505 (2)	148.96 (9)
O7—H7···C41	0.9238 (15)	2.2985 (16)	2.827 (2)	115.91 (9)

Symmetry codes: (i) −*x*+1, −*y*+1, −*z*+1; (ii) −*x*+1, *y*+1/2, −*z*+1/2.
