# Supplementary material for: A resonance-assisted intra­molecular hydrogen bond in compounds containing 2-hy­droxy-3,5-di­nitro­benzoic acid and its various deprotonated forms: redetermination of several related structures
Source: Acta Crystallogr E Crystallogr Commun. 2018 Aug 24;74(Pt 9):1344–57. doi: 10.1107/S2056989018011544 (PMC6127720; doi:10.1107/S2056989018011544)
Supplement: Supplementary file 21 [file e-74-01344-sup21.pdf]

Deposited material S1

The sections of the difference electron density maps in the regions of the hydrogen-bonded atoms in the title structures. (The structures are indicated by the refcodes

DUJZAK, the atom H3b

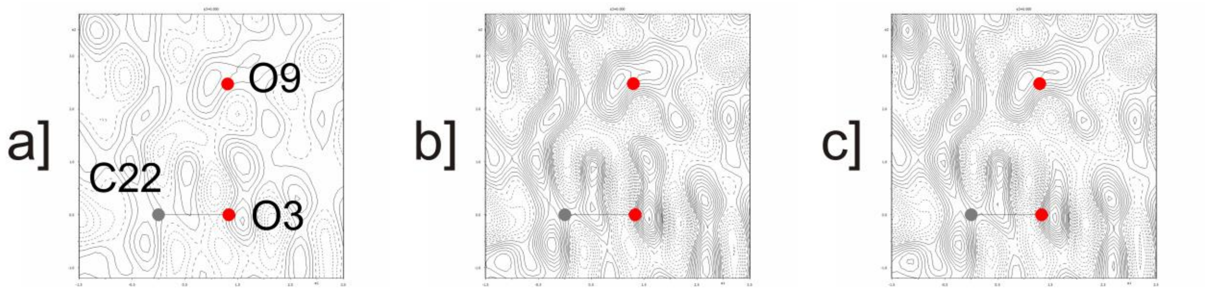

DUJZAK, the atom H1aa

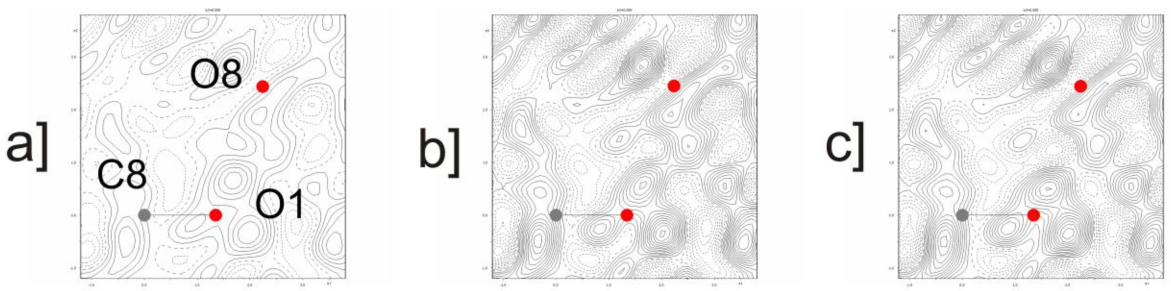

DUJZAK, the atom H2aa

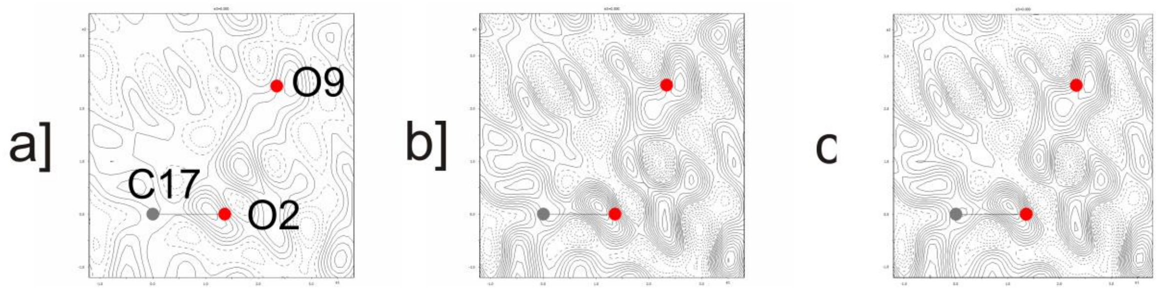

a] The difference electron-density maps without the bridging hydrogen H3b as well as the hydrogens H1aa and H2aa. b], c] The residual electron-density maps with the electron density belonging to the respective hydrogens. In Figs. b], the hydrogens were refined with the fixed positional parameters and while their displacement parameters were refined freely. In Figs. c], the positional parameters of the bridging hydrogens were refined freely while their displacement parameters were refined applying the constraint $U_{iso}(H)=1.5\ U_{eq}(O_{carrier})$. For a] and b], c], the contours are in 0.05 and 0.02 e$Å^{-3}$ increments, respectively; positive, zero and negative electron densities are indicated by continuous, dotted and dashed lines, respectively. (*JANA*2006; Petříček *et al.*, 2014).

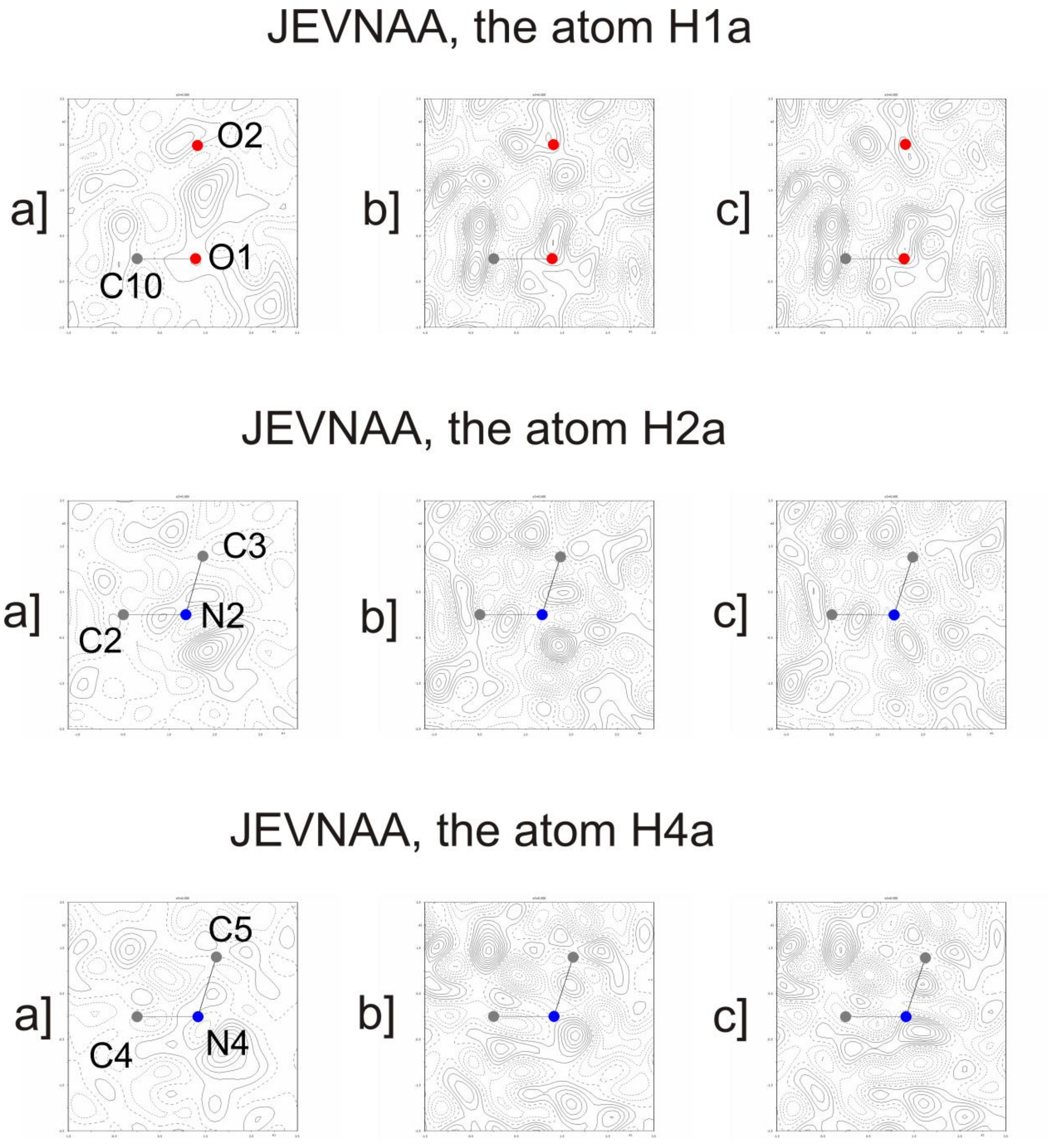

a] The difference electron-density maps without the the bridging hydrogen H1a and the secondary amine hydrogens H2a and H4a. b], c] The residual electron-density maps with the electron density belonging to the respective hydrogens. In Figs. b], the hydrogens were refined with the fixed positional parameters while their displacement parameters were refined freely. In Figs. c], the positional parameters of the bridging hydrogens were refined freely while their displacement parameters were refined applying the constraints U_{iso}(H)=1.5 U_{eq}($O_{carrier}$) or U_{iso}(H)=1.2 U_{eq}($N_{carrier}$). For a] and b], c], the contours are in 0.05 and 0.02 e$Å^{-3}$ increments, respectively; positive, zero and negative electron densities are indicated by continuous, dotted and dashed lines, respectively. (*JANA*2006; Petříček *et al*., 2014).

LUDFUL, the atom H3a

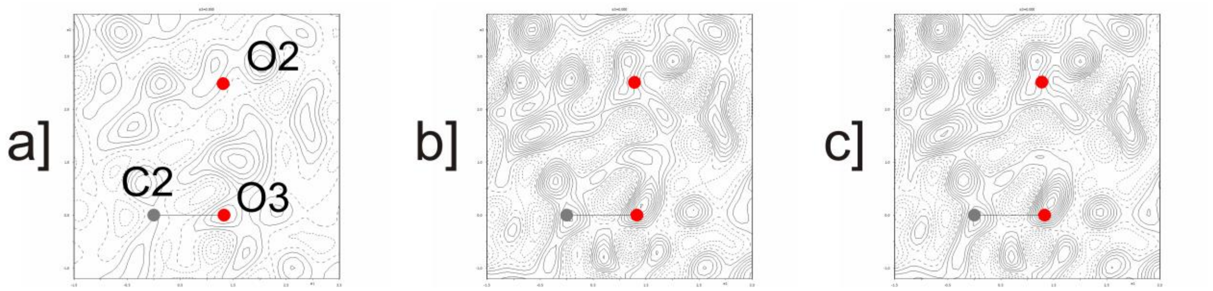

LUDFUL, the atom H1a

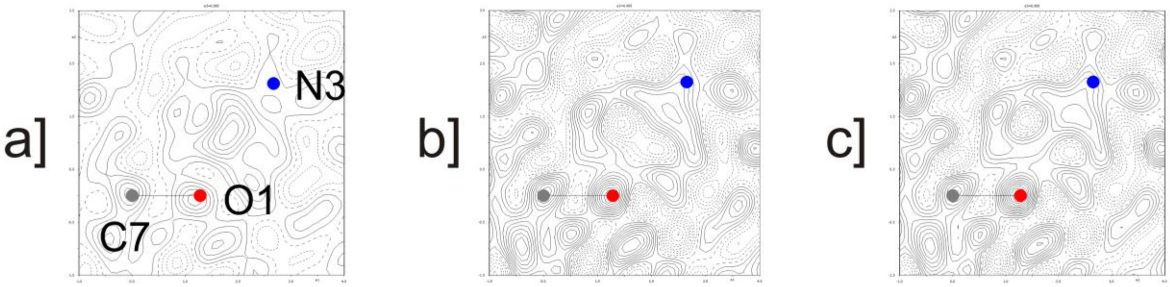

a] The difference electron-density maps without the bridging hydrogen H3a and the hydrogen H1a. b], c] The residual electron-density maps with the electron density belonging to the respective hydrogens. In Figs. b], the bridging hydrogens were refined with the fixed positional parameters while their displacement parameters were refined. In Figs. c], the positional parameters of the bridging hydrogens were refined freely while their displacement parameters were refined applying the constraints U_{iso}(H3a)=1.5 U_{eq}(O3) and U_{iso}(H1a)=1.5 U_{eq}(O1). For a] and b], c], the contours are in 0.05 and 0.02 e$Å^{-3}$ increments, respectively; positive, zero and negative electron densities are indicated by continuous, dotted and dashed lines, respectively. (*JANA*2006; Petříček *et al.*, 2014).

NUQVEB, the atoms H1O1-H1O7

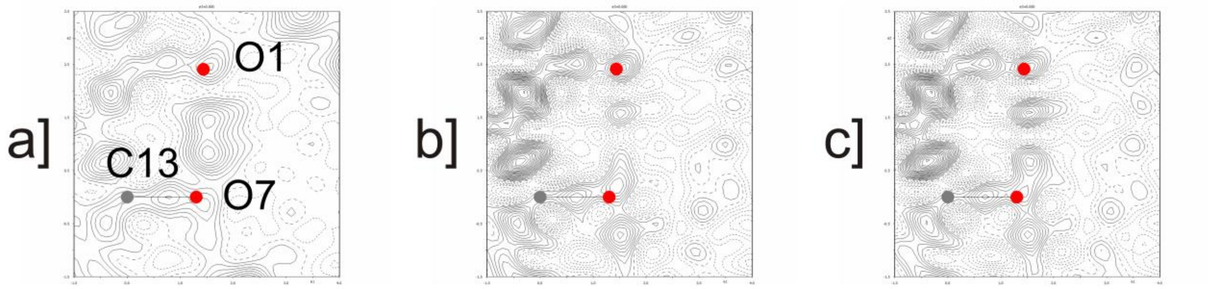

NUQVEB, the atom H1

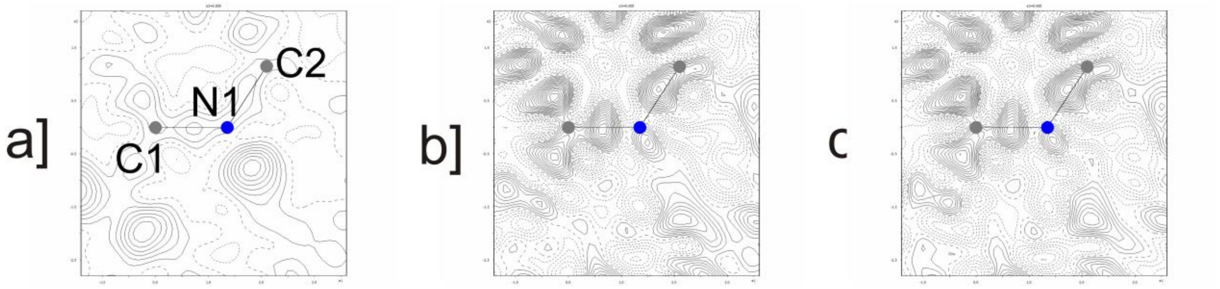

NUQVEB, the atoms H2a and H2b

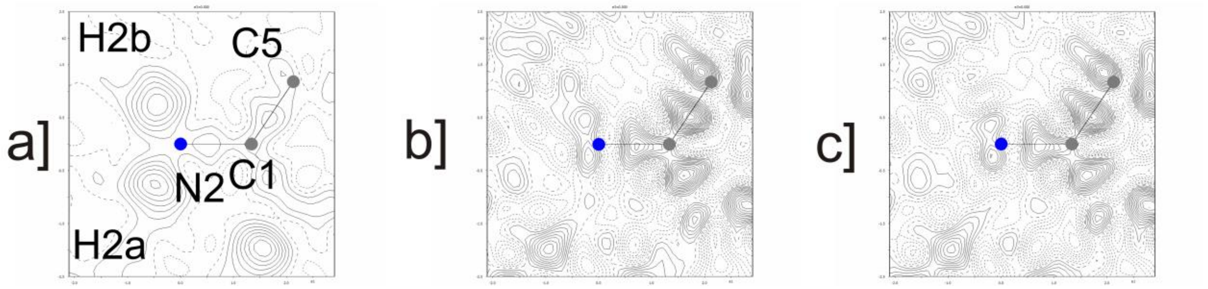

a] The difference electron-density maps without the bridging hydrogens H1O1, H1O7, the secondary amine hydrogen H1 and the primary amine hydrogens H2a and H2b. b], c] The residual electron-density maps with the electron density belonging to the respective hydrogens. In Figs. b], the hydrogens were refined with the fixed positional parameters while their displacement parameters were refined freely. In Fig. c], the positional parameters of the hydrogens were refined freely while their respective displacement parameters were constrained: $U_{iso}(H)=1.5\ U_{eq}(O_{carrier})$ or $U_{iso}(H)=1.2\ U_{eq}(N_{carrier})$. For the atoms H1O1, H1O7, the contours are in 0.05 and 0.02 e$Å^{-3}$ increments in Figures a] and b], c], respectively; for the atoms H1 and H2a, H2b , the contours are in 0.10 and 0.02 e$Å^{-3}$ increments in Figures a] and b], c], respectively. Positive, zero and negative electron densities are indicated by continuous, dotted and dashed lines, respectively. (*JANA*2006; Petříček *et al.*, 2014).

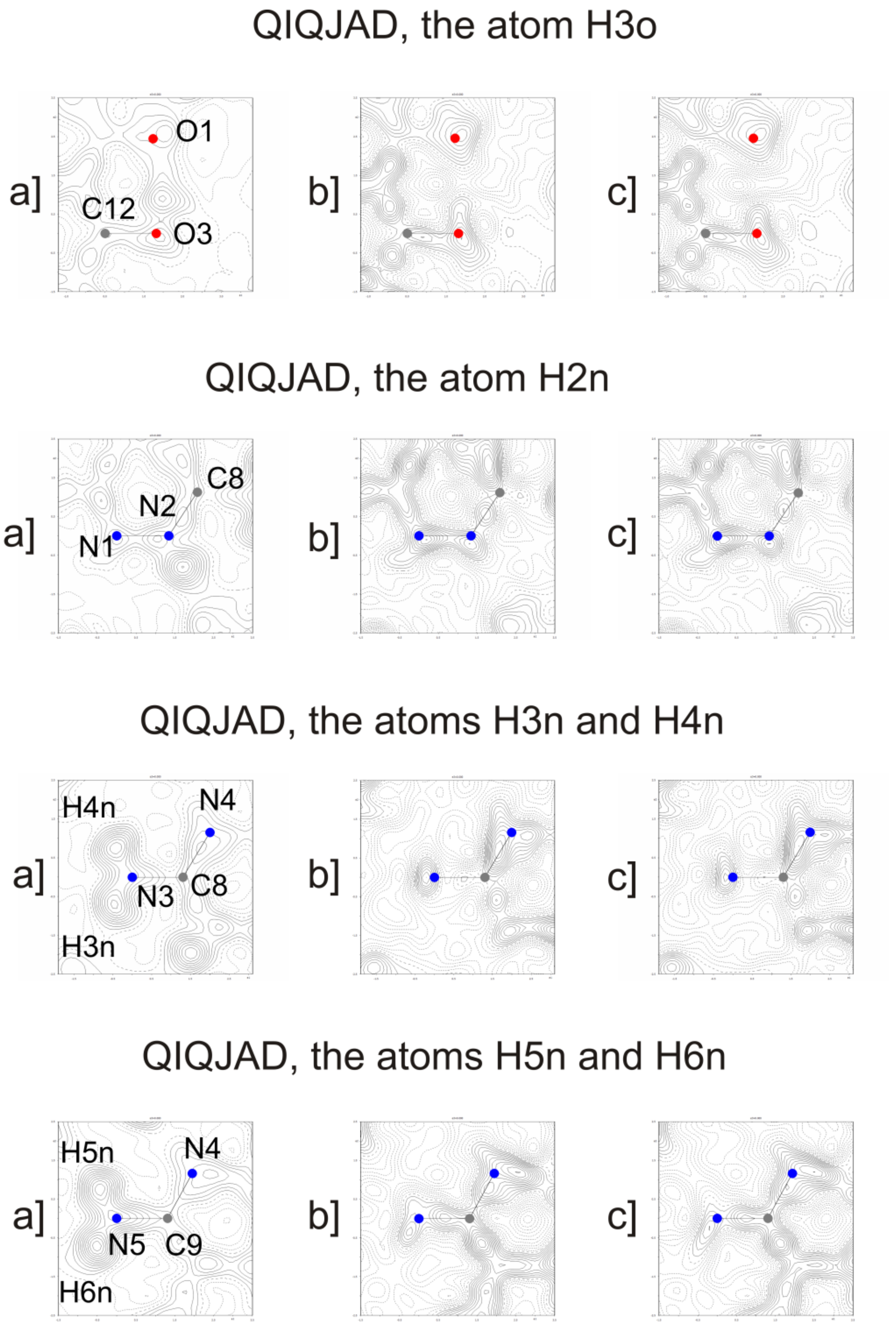

a] The difference electron-density maps without the bridging hydrogen H3o, the secondary amine hydrogen H2n and the primary hydrogens H3n, H4n, H5n and H6n. b], c] The residual electron-density maps with the electron density belonging to the respective hydrogens. In Figs. b], the hydrogens were refined with the fixed positional parameters while their displacement parameters were refined freely. In Figs. c], the positional parameters of the bridging hydrogens were refined freely while their displacement parameters were refined applying the constraints U_{iso}(H)=1.5 U_{eq}($O_{carrier}$) or U_{iso}(H)=1.2 U_{eq}($N_{carrier}$). For Figures a] and b], c], the contours are in 0.05 and 0.02 e$Å^{-3}$ increments, respectively. Positive, zero and negative electron densities are indicated by continuous, dotted and dashed lines, respectively. (*JANA*2006; Petříček et al., 2014).

SAFGUD, the atom H7

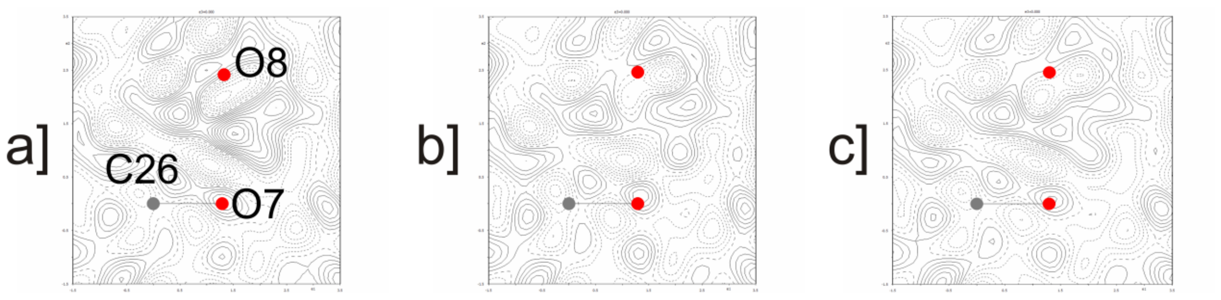

a] The difference electron-density map without the bridging hydrogen H7. The residual electron-density map with the electron density belonging to the respective bridging hydrogen. In Fig. b], the bridging hydrogen was refined with the fixed positional parameters while its displacement parameter was refined freely. In Fig. c], the positional parameters of the bridging hydrogen were refined freely while its displacement parameter was constrained as U_{iso}(H7)=1.5 U_{eq}(O7). For a] and b], c], the contours are in 0.05 increments; positive, zero and negative electron densities are indicated by continuous, dotted and dashed lines, respectively. (*JANA*2006; Petříček *et al.*, 2014).

SEDKET, the atom H2a

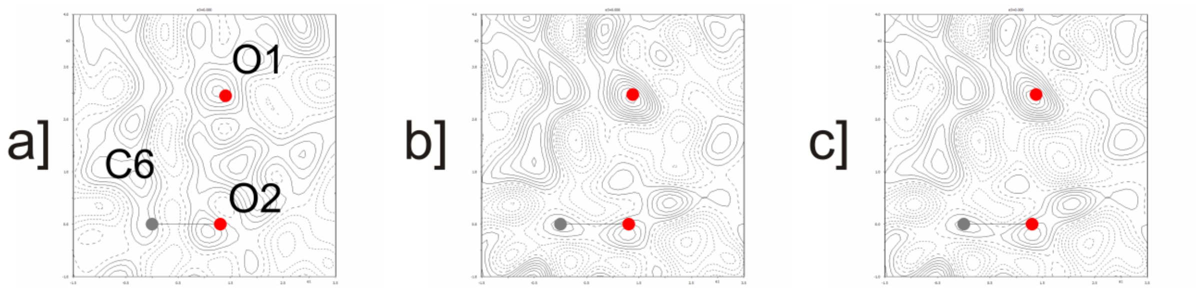

SEDKET, the atoms H1 and H2

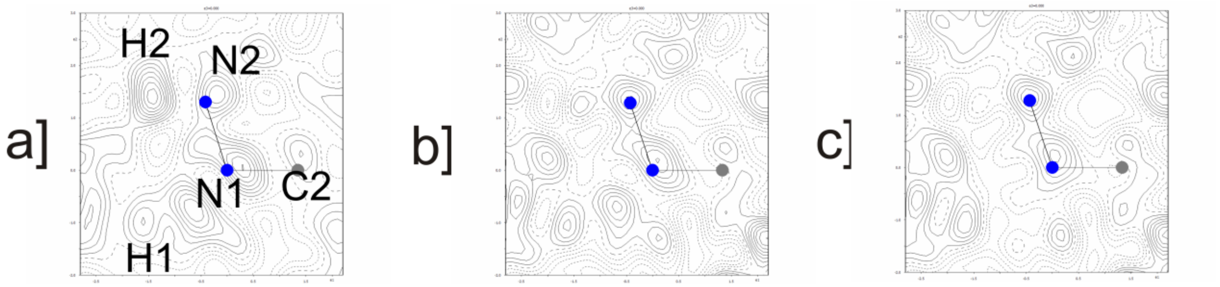

a] The difference electron-density maps without the bridging hydrogen (H2a) and the secondary amine hydrogens H1 and H2. b], c] The residual electron-density maps with the respective hydrogens included into the model. In Figs. b], the positional parameters of the hydrogens were fixed while their displacement parameters were refined freely. In Figs. c], the positional parameters of the hydrogens were refined freely while their displacement parameters were constrained: U_{iso}(H)=1.5 U_{eq}($O_{carrier}$) or U_{iso}(H)=1.2 U_{eq}($N_{carrier}$). For Figures a] and b], c] the contours are in 0.02 and 0.01 e$Å^{-3}$ increments, respectively. Positive, zero and negative electron densities are indicated by continuous, dotted and dashed lines, respectively. (*JANA*2006; Petříček *et al.*, 2014).

TIYZIM, the atom H2b

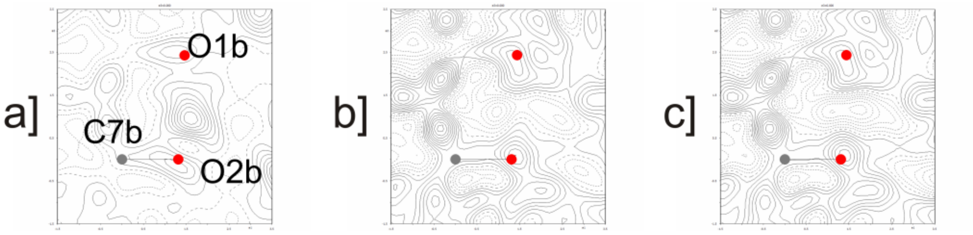

TIYZIM, the atom H3aa

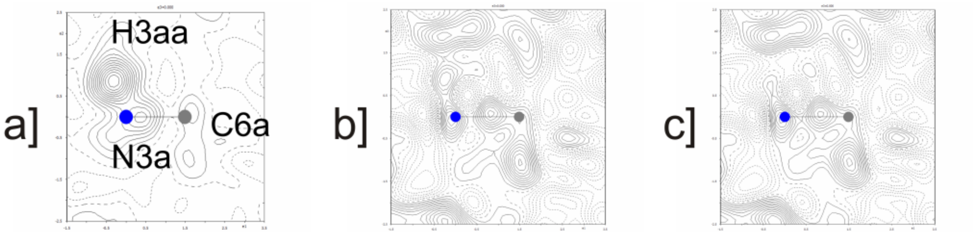

TIYZIM, the atom H3ab

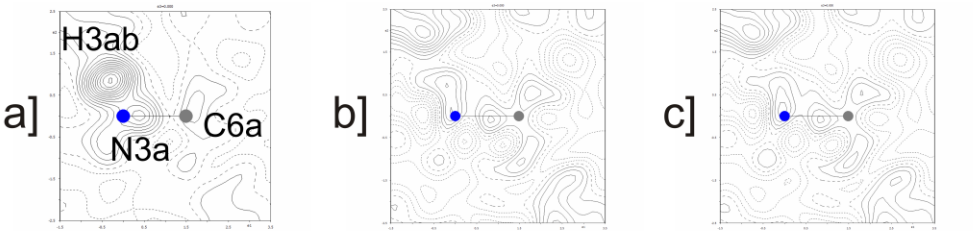

TIYZIM, the atom H3ac

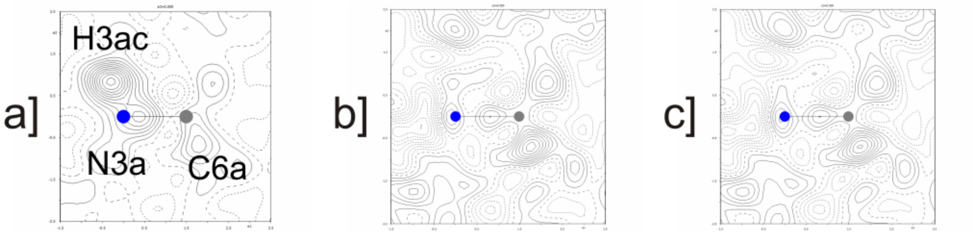

a] The difference electron-density maps without the bridging hydrogen H2b and the ammonium hydrogens (H3aa, H3ab and H3ac). b], c] The residual electron-density maps with the respective hydrogens included into the model. In Figs. b], the positional parameters of the hydrogens were fixed while their displacement parameters were refined freely. c] In Figs. c, the positional parameters of the hydrogens were refined freely while their displacement parameters were constrained: U_{iso}(H)=1.5 U_{eq}($O_{carrier}$) or U_{iso}(H)=1.2 U_{eq}($N_{carrier}$). For Figs. a], the contours are in 0.05 eÅ^{-3} increments. For Figs. b] and c] the contours are in 0.02 increments for the atoms H2b, H3ab as well as for H3ac and in 0.01 eÅ^{-3} increments for the atoms H3aa. Positive, zero and negative electron densities are indicated by continuous, dotted and dashed lines, respectively. (*JANA*2006; Petříček *et al.*, 2014).

TUJPEV, the atom H6a

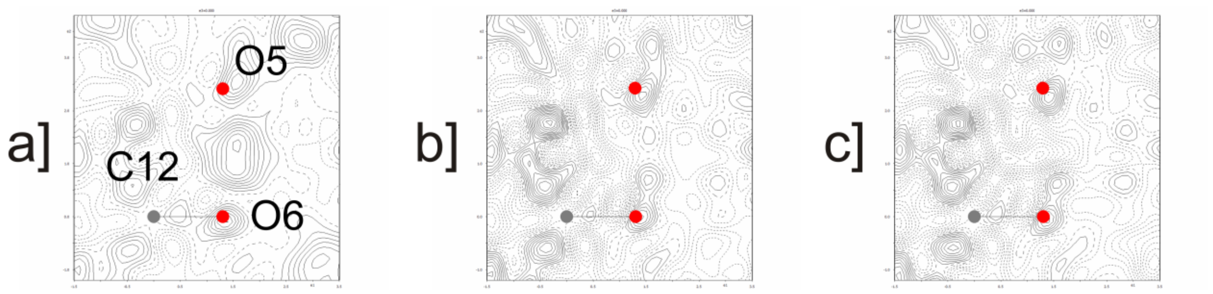

TUJPEV, the atom H2a

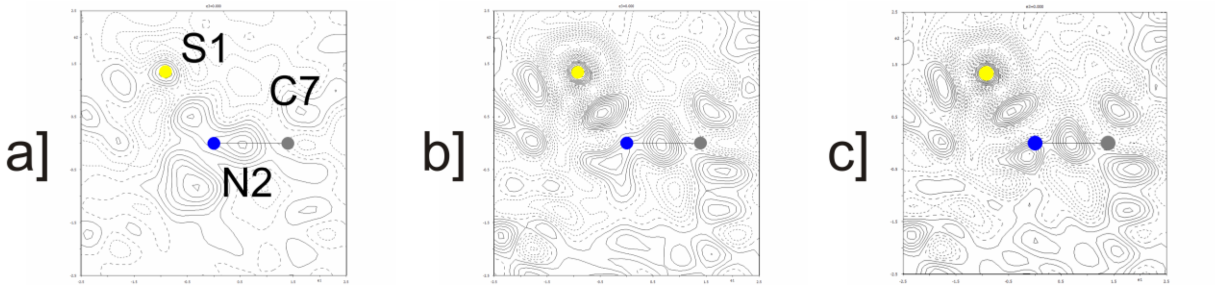

a] The difference electron-density maps without the electron density belonging to the bridging hydrogen H6a and the secondary amine hydrogen H2a. b], c] The residual electron-density maps with the respective hydrogens included into the model. In Figs. b], the positional parameters of the hydrogens were fixed while their displacement parameters were refined freely. c] The positional parameters of the hydrogens were refined freely while their displacement parameters were constrained: $U_{iso}(H)=1.5\ U_{eq}(O_{carrier})$ or $U_{iso}(H)=1.2\ U_{eq}(N_{carrier})$. For Figures a], the contours are in 0.04 and 0.05 eÅ^{-3} increments for the regions with the atoms H6a and H2a, respectively. For the Figures b], c], the contours are in 0.02 eÅ^{-3} increments; positive, zero and negative electron densities are indicated by continuous, dotted and dashed lines, respectively. (*JANA*2006; Petříček *et al.*, 2014).

TUJPEV, the atom H1a

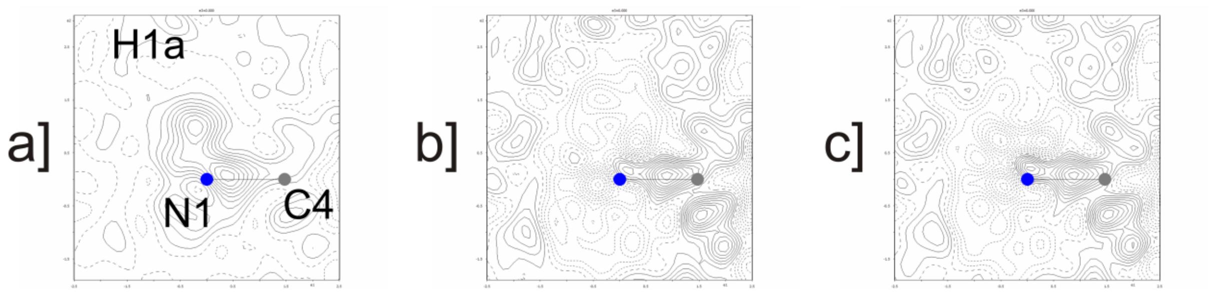

TUJPEV, the atom H1b

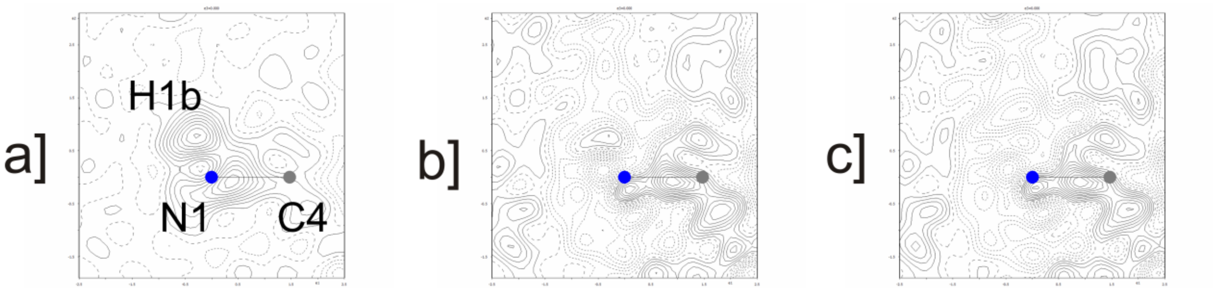

TUJPEV, the atom H1c

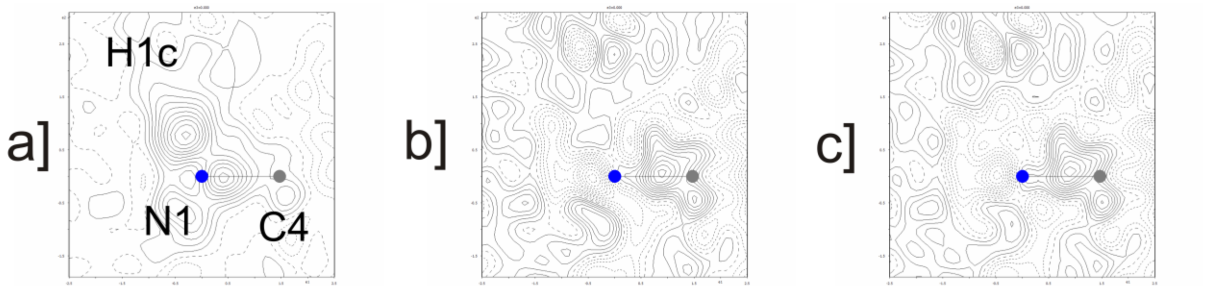

a] The difference electron-density maps without the electron density belonging to the ammonium hydrogens H1a, H1b and H1c. b], c] The residual electron-density maps with the respective hydrogens included into the model. In Figs. b], the positional parameters of the hydrogens were fixed while their displacement parameters were refined freely. c] The positional parameters of the hydrogens were refined freely while their displacement parameters were constrained: $U_{iso}(H)=1.2\ U_{eq}(N_{carrier})$. In Figures a] and b], c], the contours are in 0.05 and 0.02 e$Å^{-3}$ increments; positive, zero and negative electron densities are indicated by continuous, dotted and dashed lines, respectively. (*JANA*2006; Petříček *et al.*, 2014).

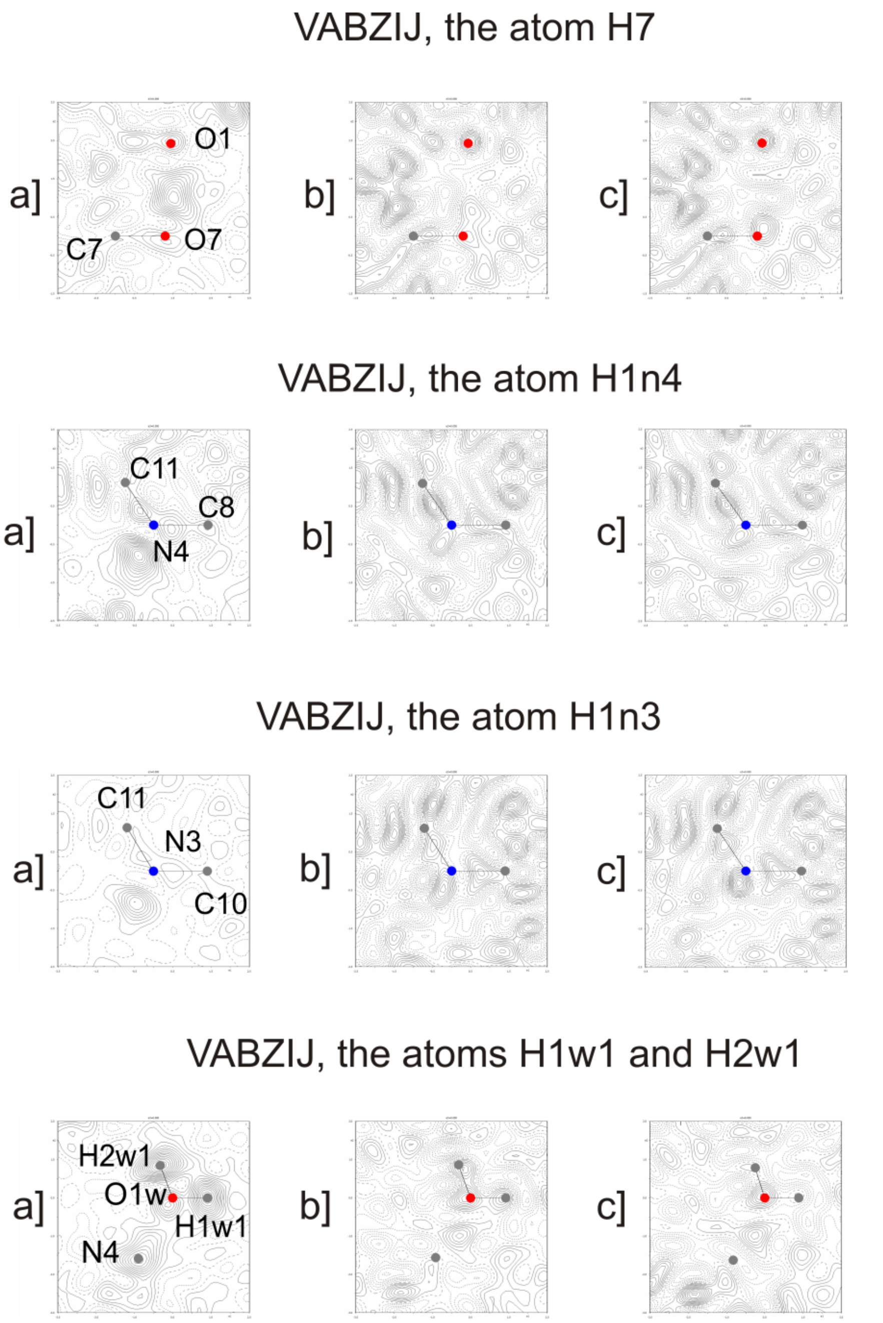

a] The difference electron-density maps without the electron density belonging to the bridging hydrogen H7, the secondary amine hydrogens H1n3, H1n4 and the water hydrogens H1w1 and H2w1. b], c] The residual electron-density maps with the respective hydrogens included into the model. In Figs. b], the positional parameters of the hydrogens were fixed while the displacement parameters were refined freely. In Figs. c], the positional parameters of the hydrogens were refined freely while their displacement parameters were constrained: $U_{iso}(H)=1.5\ U_{eq}(O_{carrier})$ or $U_{iso}(H)=1.2\ U_{eq}(N_{carrier})$. In Figure a], the contours are in 0.05 eÅ^{-3} increments for the atoms H7, H1n4, H1w1 and H2w1 and in 0.1 eÅ^{-3} increments for the atoms H1n3. In Figs. b], c], the contours are in 0.02 eÅ^{-3} increments; positive, zero and negative electron densities are indicated by continuous, dotted and dashed lines, respectively. (JANA2006; Petříček et al., 2014). (*JANA*2006; Petříček *et al.*, 2014).

WADXOR, the atom H21b

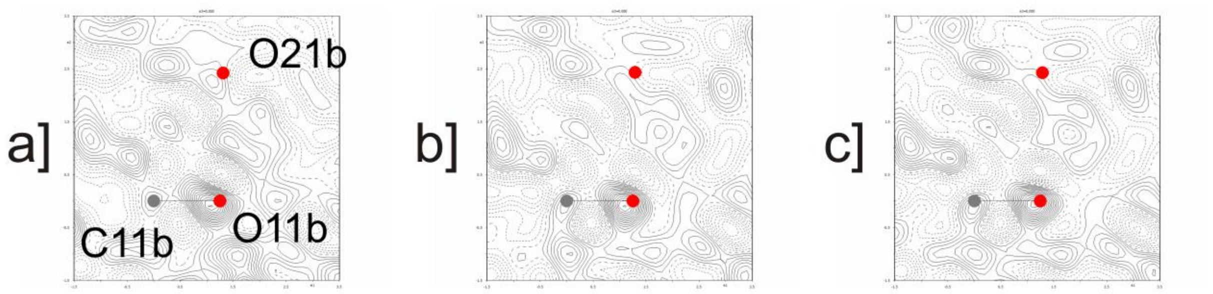

WADXOR, the atom H2b

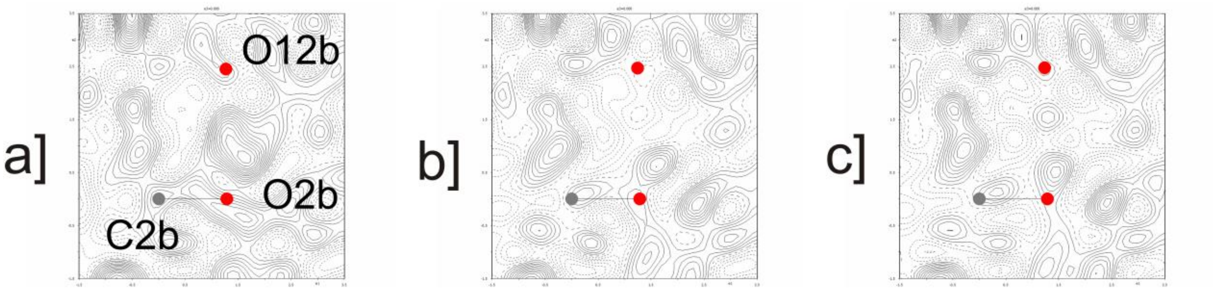

WADXOR, the atom H8a

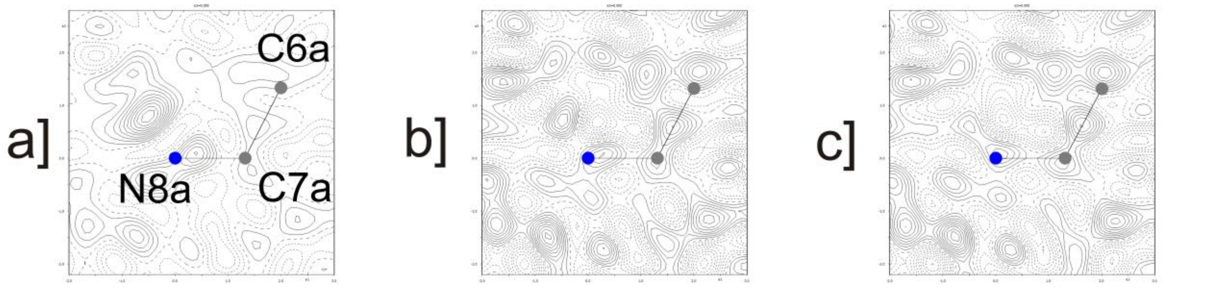

a] The difference electron-density maps without the electron density belonging to the bridging hydrogens H21b and H2b and to the secondary amine hydrogen H8a. b], c] The residual electron density maps with the respective hydrogens included into the model. In Figs. b], the positional parameters of the hydrogens were fixed while their displacement parameters were refined freely. In Figs. c], the positional parameters of the hydrogens were refined freely while their displacement parameters were constrained: U_{iso}(H)=1.5 U_{eq}($O_{carrier}$) or U_{iso}(H)=1.2 U_{eq}($N_{carrier}$). In Figures a], the contours are in 0.02 e$Å^{-3}$ increments for the atoms H21b and H2b while the contours are in 0.05 e$Å^{-3}$ increments for the atom H8a. In Figs. b] and c], the contours are in 0.02 e$Å^{-3}$ increments; positive, zero and negative electron densities are indicated by continuous, dotted and dashed lines, respectively. (*JANA*2006; Petříček *et al*., 2014).

YAXPOE, the atom H7

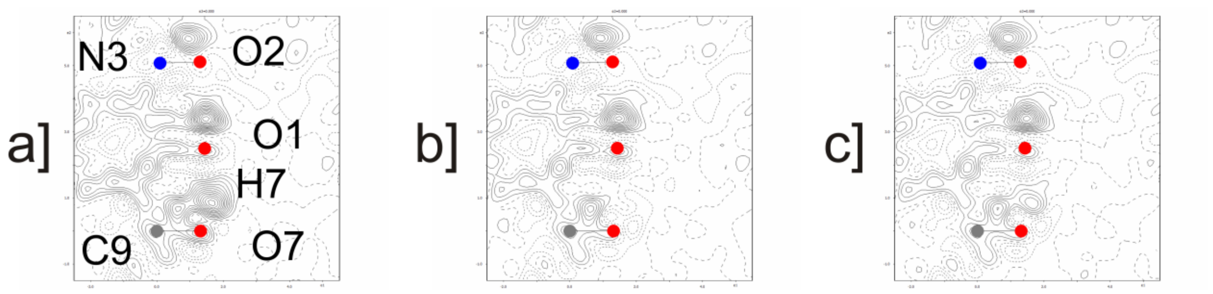

YAXPOE, the atom H71

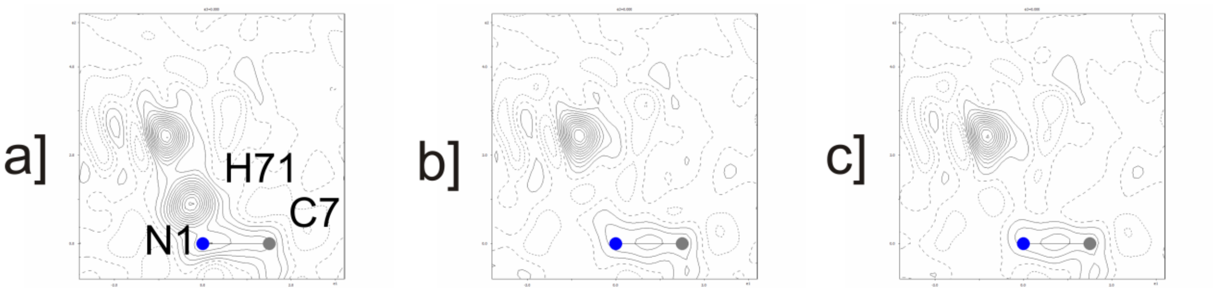

a] The difference electron-density maps without the electron density belonging to the bridging hydrogen H7 and the amine hydrogen H71. b], c] The residual electron-density maps with the respective hydrogens are included into the model. In Figs. b], the positional parameters of the hydrogens were fixed while their displacement parameters were refined. In Figs. c], the positional parameters of the hydrogens were refined freely while their displacement parameters were constrained: U_{iso}(H)=1.5 U_{eq}($O_{carrier}$) or U_{iso}(H)=1.2 U_{eq}($N_{carrier}$). In Figures a], b] and c], the contours are in 0.05 e$Å^{-3}$ increments; positive, zero and negative electron densities are indicated by continuous, dotted and dashed lines, respectively. Note: There are present peaks of the electron density which are difficult to interpret. (JANA2006; Petříček *et al.*, 2014).
